# Structural Basis of Metallo-β-lactamase Inhibition by *N*-Sulfamoylpyrrole-2-carboxylates

**DOI:** 10.1021/acsinfecdis.1c00104

**Published:** 2021-05-18

**Authors:** Alistair J. M. Farley, Yuri Ermolovich, Karina Calvopiña, Patrick Rabe, Tharindi Panduwawala, Jürgen Brem, Fredrik Björkling, Christopher J. Schofield

**Affiliations:** † Department of Chemistry, Chemistry Research Laboratory and the Ineos Institute for Antimicrobial Research, University of Oxford, 12 Mansfield Road, Oxford OX1 3TA, United Kingdom; ‡ Department of Drug Design and Pharmacology, Faculty of Health and Medical Sciences, University of Copenhagen, DK-2100 Copenhagen, Denmark

**Keywords:** antimicrobial resistance, sulfonamide, metallo-β-lactamase, taniborbactam, NDM-1

## Abstract

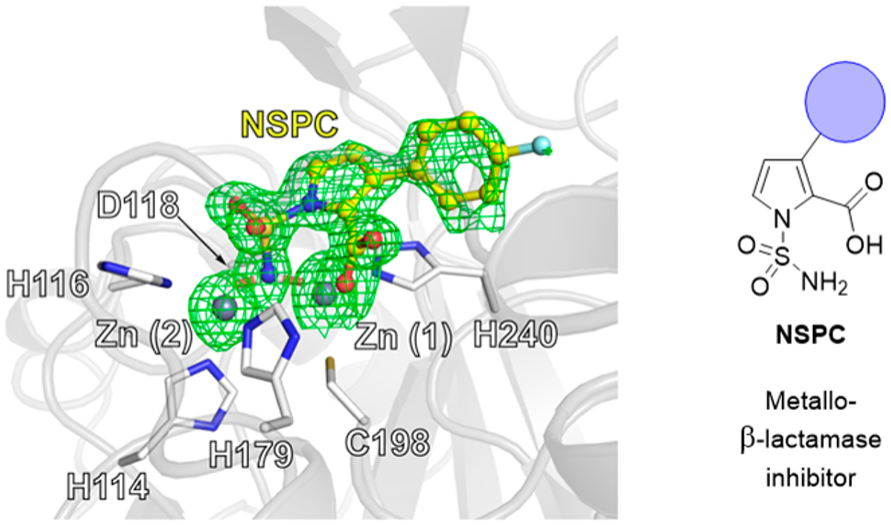

Metallo-β-lactamases (MBLs) can efficiently catalyze the hydrolysis of all classes of β-lactam antibiotics except monobactams. While serine-β-lactamase (SBL) inhibitors (e.g., clavulanic acid, avibactam) are established for clinical use, no such MBL inhibitors are available. We report on the synthesis and mechanism of inhibition of *N*-sulfamoylpyrrole-2-carboxylates (NSPCs) which are potent inhibitors of clinically relevant B1 subclass MBLs, including NDM-1. Crystallography reveals that the *N*-sulfamoyl NH_2_ group displaces the dizinc bridging hydroxide/water of the B1 MBLs. Comparison of crystal structures of an NSPC and taniborbactam (VRNX-5133), presently in Phase III clinical trials, shows similar binding modes for the NSPC and the cyclic boronate ring systems. The presence of an NSPC restores meropenem efficacy in clinically derived *E. coli* and *K. pneumoniae bla*NDM-1. The results support the potential of NSPCs and related compounds as efficient MBL inhibitors, though further optimization is required for their clinical development.

The β-lactams are one of the most important antibacterial classes;^1^ however, their efficacy is increasingly being eroded by resistance, most importantly by β-lactamases.^2^ Even carbapenems, often used as “last resort” antibiotics, are often no longer effective due to production of carbapenemases by Enterobacteriaceae strains.^3^ Ambler class A, C, and D β-lactamases are nucleophilic serine enzymes (serine-β-lactamases, SBLs), whereas class B are metallo-β-lactamases (MBLs).^4^ Combinations of a β-lactam antibiotic and a β-lactam containing SBL inhibitor have long been used as a treatment option for bacterial infections; however, no MBL combinations are clinically approved.

Presently, only a few subclasses of SBLs,^5,6^ e.g. Class A KPCs and Class D OXAs, are reported to efficiently hydrolyze carbapenems, and these can be countered by clinically available SBL inhibitors, e.g. avibactam.^7,8^ Class B MBLs, however, can hydrolyze all carbapenems and β-lactam containing SBL inhibitors.^9−11^ MBL inhibition is challenging in part because of the need to obtain activity against a range of relevant enzymes, which vary in their active site details.^12,13^ Subclass B1 and B3 MBLs are dizinc ion enzymes, whereas B2 MBLs employ one zinc ion. The B1 MBLs are the most important from a clinical perspective and include the IMP (imipenemase), NDM (New Delhi MBL), and VIM (Verona integron-encoded MBL) MBL subfamilies.^14^

Reported MBL inhibitors include bicyclic boronates, thiols, and succinate derivatives (Figure 1).^15,16^ Recently, substituted pyrroles and related compounds have been described as MBL inhibitors in the patent and scientific literature.^17−20^ Wachino et al. have reported that substituted pyrroles and furans bearing α-carboxylic acid and *N*-sulfamoyl functional groups are effective MBL inhibitors, in particular of the B1 subclass.^21^*N*-1 Sulfamoylpyrrole-2-carboxylates have also been reported as B1 MBL inhibitors,^22^ though no structures of them in complex with MBLs are reported. Structurally related sulfonamide-based inhibitors of metalloenzymes are used therapeutically, e.g. as carbonic anhydrase inhibitors with broad clinical utility.^23−25^ The *N*-sulfamoylpyrrole compounds are of mechanistic interest because the (initial) binding mode of some classes of potent β-lactamase inhibitors can mimic that of substrates (e.g., clavulanic acid) or tetrahedral intermediates (boronates).^26^ We envisaged that the approximately tetrahedral geometry about the sulfamoyl sulfur^27,28^ may mimic the tetrahedral intermediate formed during β-lactam hydrolysis, and the Lewis basicity of the oxygen and nitrogen atoms may enable effective coordination to the zinc ion. Here, we report on the mechanism of action and B1 MBL potencies of *N*-sulfamoyl-substituted pyrrole-2-carboxylic acids (NSPCs).

**Figure 1:**
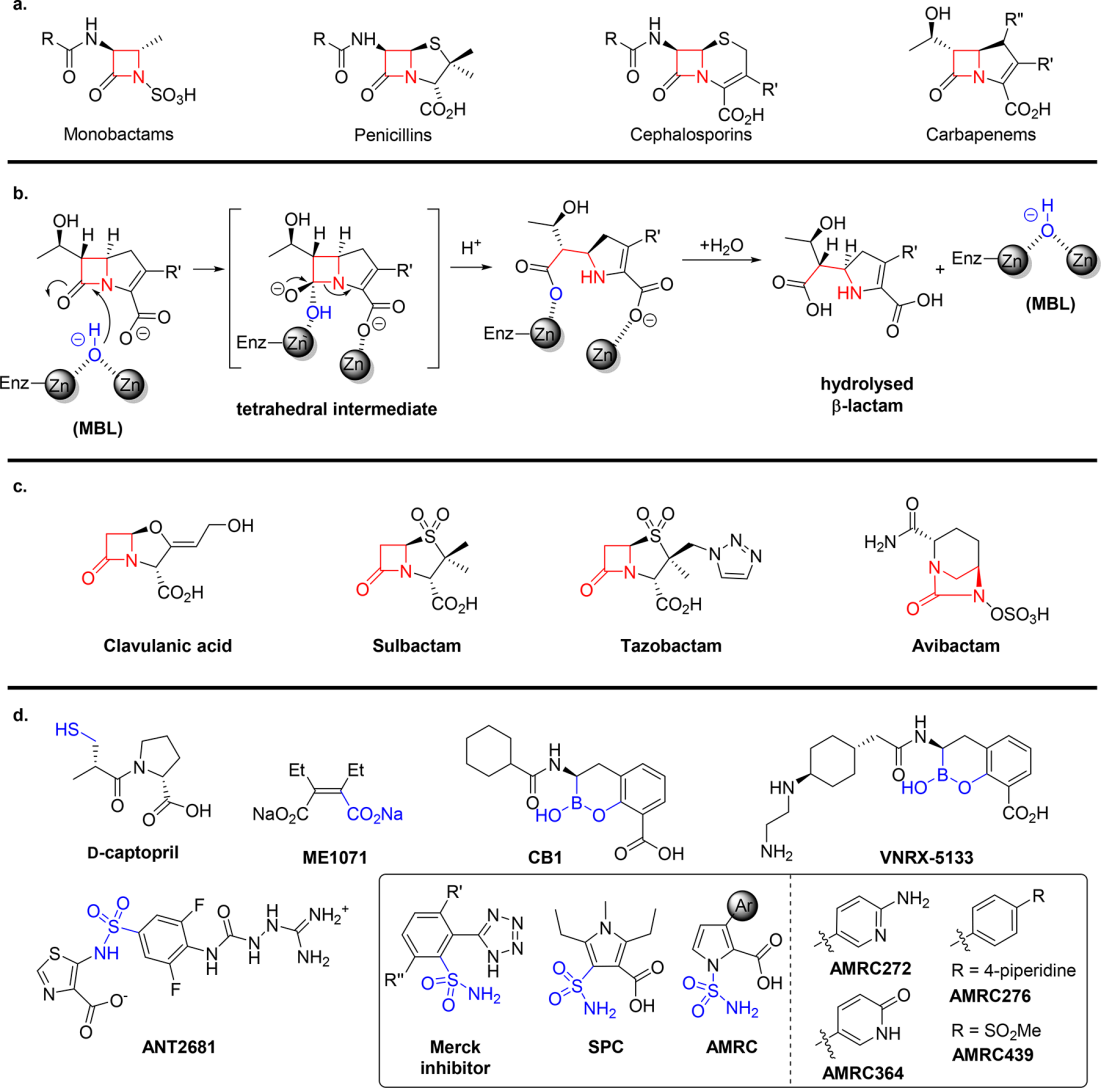
(a) Classes of β-lactam antibiotics. (b) Outline of the MBL hydrolysis mechanism. Ligands around the zinc ions are not shown for clarity. (c) Representative SBL inhibitors. (d) Representative MBL inhibitors^29−31^ with sulfonamide ANT2681^19^ and related sulfonamide and sulfamoyl inhibitors.^21,22,32^ Zinc-chelating functional groups are highlighted in blue.

## Results and Discussion

**Scheme 1:**
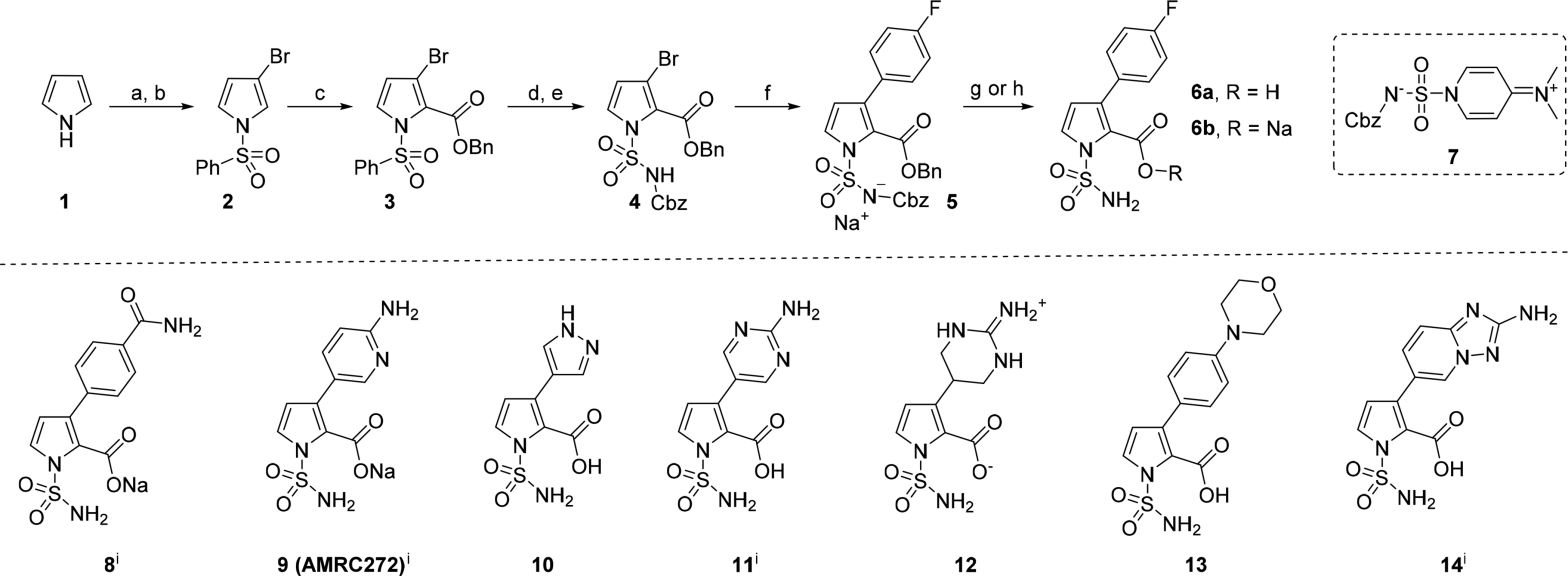
Synthesis of **6a** and NSPCs **8-14** (a) NaH, then PhSO_2_Cl, DMF, 0 °C, 2 h; (b) Br_2_, AcOH, reflux, 1 h, 54% yield over two steps; (c) *i-*Pr_2_NLi, then CbzCl, THF, −78 to 0 °C; (d) 1 M TBAF, THF, rt, 2 h, 63% over two steps; (e) NaH, then **7**, THF, 0 °C to reflux, 4 h, 69%; (f) 4-FC_6_H_4_B(OH)_2_, 5% Pd(dppf)Cl_2_, Na_2_CO_3_, dioxane: H_2_O 2:1, MW, 100 °C, 3 h, 70%; (g) 1 M aq HCl, then 10% Pd/C, H_2_ atmosphere, MeOH, rt, overnight, 69%; (h) 10% Pd/C, H_2_ atmosphere, MeOH, rt, overnight, 93%; (i) structure previously disclosed.

We targeted the synthesis of NSPC **6a**, which has a *para*-fluorophenyl substitution at its C3 position, because this substituent has been identified as being preferred in a related series of published pyrrole inhibitors (Scheme 1).^33,34^ We aimed to employ mild hydrogenolytic deprotection to prepare the NSPCs because of potential competitive decarboxylation of pyrrole-2-carboxylic acids^35,36^ and *N*1-sulfonyl group cleavage under acidic or basic conditions.^35−37^ The efficient synthesis of **6a** was readily achieved in seven steps from pyrrole (**1**) (12% overall yield, Scheme 1). Initial *N*-sulfonylation of **1** with PhSO_2_Cl was followed by regioselective electrophilic C3-bromination using Br_2_. Subsequent directed *ortho*-metalation and electrophilic trapping with benzylchloroformate (CbzCl) gave C2-substituted benzyl ester **3**. The *N*-Cbz protected sulfamoyl group was installed in good yield by tetrabutylammonium fluoride (TBAF)-mediated *N*-sulfonyl deprotection, followed by deprotonation of the pyrrole NH with sodium hydride and then electrophilic trapping with zwitterionic sulfamoylating reagent **7**.^38^ Subsequent Pd-catalyzed Suzuki–Miyaura cross-coupling with 4-fluorophenylboronic acid afforded the C3-aryl derivative **5** as a sodium sulfonylazanide salt which, upon hydrogenation, gave sodium carboxylate **6b** (93%). The free acid **6a** was obtained from **5** by acidification with aqueous HCl, followed by global deprotection with Pd/C/H_2_ and purification by reverse phase HPLC in 69% yield.

With a robust synthesis of **6** in hand, we synthesized seven other NSPC derivatives varying the C3-pyrrole substituent, with bromopyrrole **4** providing a convenient vector for late-stage diversification with aryl and heteroaryl groups via Suzuki–Miyaura coupling, followed by hydrogenation (**8**–**14**, see Supporting Information). Unexpectedly, under the hydrogenation conditions used for the preparation of aminopyrimidine **11**, near equimolar quantities of zwitterionic cyclic guanidine **12** were also formed due to over-reduction;^39^ both products were separated by preparative HPLC. For the preparation of **14**, it was necessary to first install the pinacol boronate ester at the C3 position of pyrrole **4** by Pd-catalyzed Miyaura borylation; the intermediate boronate ester then underwent coupling with the commercial heteroaryl bromide.

The NSPCs were screened against four of the currently most clinically relevant B1 MBLs, i.e. VIM-1, VIM-2, NDM-1, and IMP-1 (Table 1), using an assay employing the “fluorogenic” cephalosporin FC5.^40^ The NSPCs inhibit all 4 MBLs, with potencies in the submicromolar range (pIC_50_ s 6.5–8.5), though manifesting different inhibition profiles. Notably, the cyclic guanidine **12** is a highly potent VIM-1 inhibitor (pIC_50_ 8.5), with a similar potency to the bicyclic boronate taniborbactam (formerly VNRX-5133), which is in Phase III clinical trials.^41^ Interestingly, the cyclic guanidine **12** showed higher activity toward VIM-1 than the unsaturated aminopyrimidine **11**; however, **12** is a less potent inhibitor of NDM-1. Most of the NSPCs showed submicromolar activity (IC_50_) for inhibition of NDM-1 and VIM-2, and **6a**, **6b**, **10**, and **13** showed nanomolar potency against NDM-1. Furthermore, some NSPCs are ∼150- to >1500-fold more potent (pIC_50_ values 7.3–9.2) than the bicyclic boronates CB2 or taniborbactam (pIC_50_ 6.0 and 5.6, respectively) against IMP-1. Within the compound set tested by us, no stand-out compound potently inhibiting all four MBLs was identified, revealing the scope for further optimization of the NSPC scaffold.

**Table 1:** Activity of *N*-Sulfamoyl Pyrrole Carboxylate Derivatives against Clinically Relevant MBLs

	pIC_50_			
	VIM-1	NDM-1	VIM-2	IMP-1
CB2	7.1^42^	7.5^43^	8.5^43^	6.0^43^
taniborbactam	8.1^41^	8.0^41^	8.3^41^	5.6^41^
**6a**	6.9	8.1	7.7	9.2
**6b**	6.9	8.2	7.5	8.9
**8**	7.1	7.9	6.8	8.6
**9**	6.5	7.9	6.7	9
**10**	7.1	8.1	7.8	8.9
**11**	7.4	7.9	7.3	8.2
**12**	8.5	6.5	7.9	7.3
**13**	6.6	8.8	6.8	>9.2
**14**	7.4	7.9	8.2	8.3
AMRC272^a^^,^^c^		6.1^22^	4.5^22^	6.4^22^
AMRC276^a^^,^^c^		6.8^22^	4.2^22^	7.4^22^
AMRC364^a^^,^^c^		5.6^22^	4.4^22^	5.7^22^
AMRC439^a^^,^^c^		6.4^22^	4.3^22^	6.3^22^
SPC^b^		6.0^21^	7.7^21^	6.6^21^

a Determined using 100 μM nitrocefin.^22^

b Determined by measuring hydrolysis of imipenem.^21^

c Structure in Figure 1d. Assay details are given in the Supporting Information. Enzyme concentration: 100 pM (VIM-1), 20 pM (NDM-1), 20 pM (IMP-1), and 500 pM (VIM-2); the concentration of FC5 was 5 μM. Note: inhibition data are reported as pIC_50_ values (pIC_50_ = −log_10_IC_50_) and repeated in quadruplicate.

Our pIC_50_ values for **9** obtained from the fluorescence-based assay are higher than the previously reported pIC_50_ values for AMRC272 when using a nitrocefin-based assay.^22^ The discrepancy likely reflects different enzymatic assay conditions, protein constructs, and enzyme purification procedures; however, it should be noted that both assays show a clear preference for inhibition of IMP-1 over VIM-2.^22^

### Crystallography

**Figure 2:**
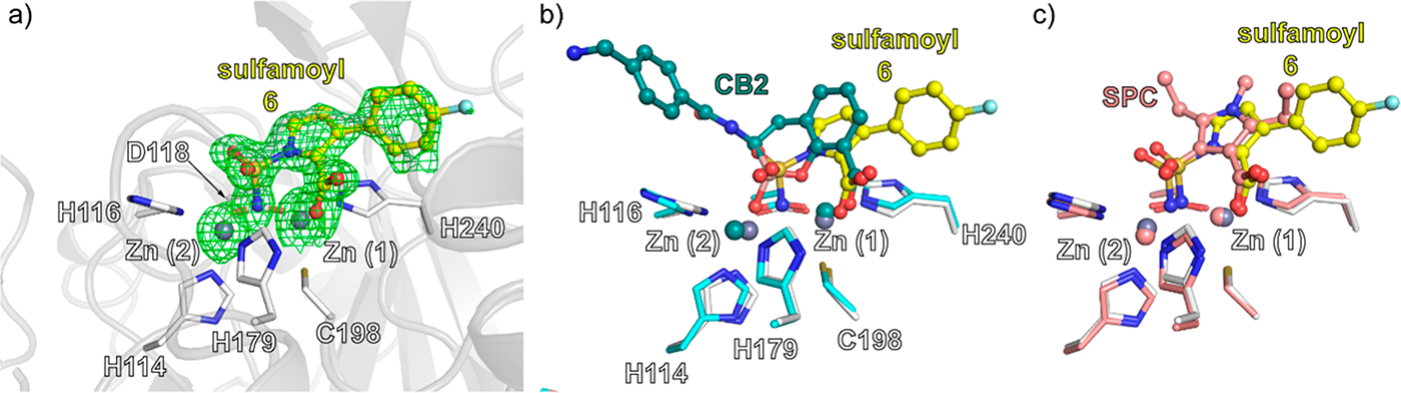
VIM-1 active site binding mode of sulfamoyl inhibitor **6** and comparison with that of a bicyclic boronate and SPC. (a) Polder omit map^48^ of VIM-1:Zn_2_:**6** (PDB: 7AYJ, 1.21 Å resolution, 3.0 σ contour level) showing the NSPC sulfamoyl NH_2_ group replaces the dizinc bridging water. (b) Superimposition of VIM-1:Zn_2_:CB2 (PDB: 7AYJ, yellow) and VIM-2:Zn_2_:CB2 (PDB: 5FQC, teal)^43^ structures reveals related binding modes. Note, whereas binding of CB2 to VIM-2 has an impact on the Zn–Zn distance compared to unligated VIM-2, the effects of binding of **6** on this distance are negligible (see Supporting Information Figure S1e–g); note also that the aryl side chains of CB2 and **6** project in different directions. (c) Superimposition of VIM-1:Zn_2_:SPC (PDB: 7AYJ, yellow) and Vim-2:Zn_2_:NSPC (PDB: 6KZN, salmon)^21^ structures reveal the same binding mode.

We investigated the NSPC ligand–enzyme interaction by crystallography and obtained a structure for VIM-1 complexed with **6** (space group: *P*12_1_ 1, 1.21 Å resolution, Figure 2). The structure was solved by molecular replacement (PDB: 5N5G),^44^ with iterative fitting of **6** at the active site. The two zinc ions (coordinated by H114, H116, D118 (Zn1), H179, C198, and H240(Zn2)) were refined with occupancies of 0.75 for both Zn1 and Zn2. The reduced occupancy for Zn1 is due to partial oxidation of Cys198 to a 3-sulfino alanine residue (Csd198), which was modeled and refined in a ratio of Cys (75%) to Csd (25%), as shown in previous work on VIM-1 (PDB 5FQA).^45^

Uncomplexed VIM-1:Zn_2_ (PDB: 5N5G)^44^ has a dizinc bridging hydroxide/water, as do other B1MBls.^26^ The NH_2_- of sulfamoyl group of **6** replaces this “hydrolytic” water, probably in its deprotonated form, though this cannot be discerned from the crystal structure. The C2-carboxylate of **6** ligates to Zn2, as observed in substrate derived complexes and those of inhibitors with analogously placed carboxylates, including CB2/VNRX-5133 (Figure 1).^41−43^

The binding mode of **6** is related to that of bicyclic boronate MBL inhibitors, (e.g., VIM-2:Zn_2_:CB2 (PDB: 5FQC, superimposition Figure 2b)),^43^ in which the binding of the two boron-bound oxygens mimics the binding modes proposed for the two oxygens in the oxyanion intermediate in MBL catalysis. However, in the NSPC complex, one of these oxygens is “replaced” by the tetrahedral sulfamoyl amino group (NR_2_-S-NH_2_ 108°; NR_2_SO_(1)_ 105°; NR_2_–S-O_(2)_ 114°, O_(1)_-S-NH_2_ 111°, see Figure S1a). Comparison of the Zn(1)–Zn(2) distances in the VIM-1:Zn_2_:**6** and the VIM-2:Zn_2_:CB2 complex reveals differences. The Zn(1)–Zn(2) distance is increased in the VIM-2:Zn_2_:CB2 complex to 4.34 Å compared to 3.47 and 3.62 Å^44^ in the unligated VIM-2:Zn_2_ (PDB: 5N5G) and VIM-1:Zn_2_ (PDB: 4NQ2) complexes, respectively^46^ (see Supporting Information Figure S1e). Binding of **6**, however, does not substantially increase the Zn(1)–Zn(2) distance, i.e. it is 3.60 Å compared to the unligated VIM-1/VIM-2. This distance is similar to those reported for a hydrolyzed VIM-1:Zn_2_:meropenem complex (PDB: 5N5I, Zn(1)–Zn(2): 3.50 Å, Figure S1d)^44^ and other unligated VIM family members.^47^

Antimicrobial susceptibility testing of the NSPCs in combination with Meropenem, following CLSI guidelines,^49,50^ was performed in a minimum inhibition concentration (MIC) antimicrobial assay format with 4 clinically relevant NDM-1 producing strains of *Escherichia coli* and *Klebsiella pneumoniae* (Table 2). At a fixed concentration of 8 μg mL^–1^, the *N*-sulfamoyl pyrroles reduce the meropenem MIC from 64 to ∼0.375 μg mL^–1^. In all cases, the MICs for the NSPCs were better than those for VNRX-5133. At a concentration of 0.5 μg mL^–1^ of the NSPC, the analogue potencies can be compared; **6** and **8**–**10** exhibit greater potency compared to the higher mass compounds **13** and **14**, bearing 4-morpholinophenyl or bicyclic heteroaromatic groups at C3, respectively. These differences may reflect differences in uptake as the pIC_50_s of **13**/**14** against NDM-1 are consistent with the other analogues (Table 1). Furthermore, the comparative data for aminopyrimidine **11** and its saturated analogue **12** show the latter is significantly less active at 0.5 μg mL^–1^, in particular with the B68-1, S117, and IR47 strains, likely reflecting their relative NDM-1 pIC_50_ values (Table 1). These data correlate with the submicromolar/nanomolar enzymatic inhibition for NDM-1 and show the ability of the series to penetrate the cell membrane, at least in the studied Enterobacteriaceae clinical isolates.

**Table 2:** MIC Values of Meropenem (MEM)-SPC Combination against NDM-1 Producing Enterobacteriaceae

strain				MIC (μg mL^–1^)									
	species, genotype	MEM	[*I*] (μg mL^–1^)	MEM VNRX^a^	MEM **6a**	MEM **6b**	MEM **8**	MEM **9**	MEM **10**	MEM **11**	MEM **12**	MEM **13**	MEM **14**
**IR57**	*E. coli*	64	0.5		1	1	0.5	1	1	1.5	4	4	1.5
	^*bla*^NDM-1		2		≤0.25	≤0.25	≤0.25	≤0.25	≤0.25	0.375	≤0.25	0.5	≤0.25
			8	0.5	≤0.25	≤0.25	≤0.25	≤0.25	≤0.25	≤0.25	0.375	0.5	≤0.25
**B68-1**	*K. pneumoniae*	64	0.5		0.5	0.5	0.5	0.5	1	1	16	1	3
	*^bla^*NDM-1		2		≤0.25	≤0.25	≤0.25	≤0.25	≤0.25	≤0.25	≤0.25	≤0.25	≤0.25
			8	0.5	≤0.25	≤0.25	≤0.25	≤0.25	≤0.25	0.5	0.5	0.5	≤0.25
**S117**	*E. coli*	256	0.5		4	2	1	1	1.5	1.5	32	8	4
	*^bla^*NDM-1		2		≤0.25	≤0.25	≤0.25	≤0.25	≤0.25	≤0.25	≤0.25	0.5	≤0.25
			8	1	≤0.25	≤0.25	≤0.25	≤0.25	≤0.25	0.5	0.5	0.5	≤0.25
**IR43**	*K. pneumoniae*	128	0.5		≤0.25	≤0.25	0.375	0.5	0.5	0.5	16	0.5	1.5
	*^bla^*NDM-1		2		≤0.25	≤0.25	≤0.25	≤0.25	≤0.25	≤0.25	≤0.25	≤0.25	≤0.25
			8	0.25	≤0.25	≤0.25	≤0.25	≤0.25	≤0.25	≤0.25	0.375	0.5	≤0.25

a MIC values of meropenem (MEM) and VRNX were at 10 μg mL^–1^.^41^ MIC experiments were repeated in triplicate.

## Conclusions

Our biochemical and microbiological results combined with recently published data^34,22^ reveal the NSPCs as promising MBL inhibitors with particularly potent (low nM) activity against NDM-1 and IMP-1 and submicromolar activity against VIM-1 and VIM-2 enzymes. The structural studies presented here define an NSPC binding mode very similar to that of the α-carboxylate- and *N*-sulfamoyl-substituted furans and pyrroles as MBL inhibitors described by Wachino et al.^21^ Thus, the likely deprotonated amino group of the tetrahedral sulfamoyl group bridges the two zinc ions replacing the hydrolytic water in a manner reflecting a tetrahedral intermediate in catalysis. The structural analyses suggest that the relatively low activity against VIM-1/VIM-2 may in part reflect differences in inhibitor (and substrate) carboxylate binding mode. Dynamics at the dizinc center of the protein, which are not observable with cryo-temperature crystal structures, may also account for the observed differences in inhibition data between the tested MBL enzymes. Indeed, the overall highly conserved active site architecture of the MBL superfamily enzymes supports a wide range of reactions, including nucleic acid hydrolysis and redox reactions, and in some cases, human MBLs are being pursued as drug targets.^51,52^ The compact and polar nature of the NSPCs and related scaffolds suggests that they may have wide utility as inhibitors of MBL superfamily enzymes. However, further derivatization of the NSPCs is warranted to increase potency and spectrum of activity toward the most abundant resistance causing MBLs to restore utility of important β-lactamase antibacterials.

## Methods

The experimental procedures describing the synthesis and characterization of the compounds, the evaluation of their biological activity (enzyme assays, *in vitro* antibacterial susceptibility testing), and X-ray crystallography studies are fully described in the Supporting Information.

### General Information

Commercially available reagents and solvents were from Merck or Fluorochem and were used as received. All manipulations with air- and moisture-sensitive compounds were carried out under a positive pressure of argon in flame-dried glassware. Reactions under microwave conditions were carried out in Biotage Initiator EXP microwave reactor with Robot Sixty sample processor.

Chromatographic separations/purifications were performed either using manually packed columns with Silica gel 60 (Merck, 15–40 μm) for dry column vacuum chromatography (DCVC)) or using Reveleris X2 Flash Chromatography Purification System (BÜCHI) with FlashPure Silica prepacked columns. Reactions were monitored by TLC on silica gel 60 F254 plates (Merck).

NMR spectra were acquired using a 600 MHz Bruker Avance III HD machine equipped with a 5 mm DCH cryoprobe and a 400 MHz Bruker Avance II equipped with a 5 mm BBFO probe. Chemical shifts were referenced to residual protio- and perdeuterio-solvent resonances (δ_H_ 7.26 and δ_C_ 77.16 for CDCl_3_; δ_H_ 2.50 and δ_C_ 39.52 for DMSO-*d*_*6*_) as internal standards for ^1^H NMR and ^13^C NMR spectra, respectively. ^19^F NMR spectra were referenced indirectly via the ^2^H signal of the lock substance (CDCl_3_ or DMSO-*d*_*6*_) and the Ξ(^19^F) value. All NMR spectra were processed with MestReNova software v. 14.1.

Low resolution mass spectrometry (LRMS) data were obtained using a Waters Acquity H-class UPLC with a Sample Manager FTN and a TUV dual wavelength detector coupled to a QDa single quadrupole analyzer using electrospray ionization (ESI). UPLC separation was achieved with a C18 reversed-phase column (Acquity UPLC BEH C18, 2.1 × 50 mm, 1.7 μm) operated at 40 °C, using a linear gradient of the binary solvent system of buffer A (H_2_O:MeCN:formic acid, 95:5:0.1 v/v/v%) to buffer B (MeCN:formic acid, 100:0.1 v/v%) from 0 to 100% B in 3.5 min, then 1 min at 100% B, maintaining a flow rate of 0.8 mL/min. High resolution mass spectra were recorded using a Bruker μTOF (ESI) spectrometer. The *m*/*z* values are reported in Daltons.

Analytical HPLC was carried out using an Ultimate HPLC system (Thermo Scientific) consisting of a LPG-3400A pump (1 mL/min), a WPS-3000SL autosampler, and a DAD-3000D diode array detector (220 and 254 nm) using a Gemini-NX C18 column (4.6 × 250 mm, 3 μm, 110 Å, Phenomenex); gradient elution 0 to 100% B (MeCN-H_2_O-TFA 90:10:0.1 v/v/v%) in solvent A (H_2_O-TFA 100:0.1 v/v%) over 15 min.

Preparative HPLC (prepHPLC) was carried out using an Ultimate HPLC system (Thermo Scientific) consisting of a LPG-3200BX pump (20 mL/min), a Rheodyne 9725i injector, a 10 mL loop, a MWD-300SD detector (220 and 254 nm), and an AFC-3000SD automated fraction collector using a Gemini-NX C18 column (21.2 × 250 mm, 5 μm, 110 Å, Phenomenex); gradient elution 0 to 100% B (MeCN-H_2_O-formic acid 90:10:0.1 v/v/v%) in solvent A (H_2_O-formic acid 100:0.1 v/v%) over 15 min (unless noted otherwise).

Data for both analytical and preparative HPLC were acquired and processed using Chromeleon software v. 6.80.

### 3-Bromo-1-(phenylsulfonyl)-1*H*-pyrrole (**2**)

#### *N*1-Sulfonylation^53^

Sodium hydride (2.20 g, 55.0 mmol, 60 wt %, 1.1 equiv) was added portionwise to the solution of pyrrole (3.47 mL, 50.0 mmol, 1.0 equiv) in dry DMF (150 mL) at 0 °C. The obtained mixture was stirred for 1 h at the same temperature (note for this step: a constant flow of nitrogen gas was used to reduce foaming). Benzenesulfonyl chloride (7.66 mL, 66.0 mmol, 1.2 equiv) was added slowly over 5 min at 0 °C; the cooling bath was removed, and the reaction was further stirred for 0.5 h at room temperature (starting pyrrole was consumed, TLC). The reaction mixture was carefully quenched with half-saturated NH_4_Cl (200 mL) at 0 °C and diluted with 200 mL of EtOAc. The organic phase was washed with water (4 × 150 mL), brine (150 mL), dried over Na_2_SO_4_, and concentrated under reduced pressure, providing 10.64 g of crude 1-(phenylsulfonyl)-1*H*-pyrrole as a beige solid which was taken through to the next step without further purification.

#### Bromination^54^

A solution of bromine (2.57 mL, 50 mmol, 1 equiv) in acetic acid (40 mL) was added dropwise to the solution of 1-(phenylsulfonyl)-1*H*-pyrrole (10.64 g, ∼50 mmol, 1 equiv) in AcOH (90 mL). The mixture was refluxed for 1 h, then cooled to rt, concentrated and coevaporated with toluene (2 × 150 mL). Purification by DCVC (5% EtOAc - heptane) afforded 11.53 g of the desired product as a purple oil which solidified upon standing. Further purification by crystallization from MeOH (20 mL) gave 7.69 g (54% from pyrrole) of **2** as a white crystalline solid.

^1^H NMR (400 MHz, CDCl_3_) δ 7.90–7.83 (m, 2H), 7.67–7.59 (m, 1H), 7.58–7.46 (m, 2H), 7.19–7.15 (m, 1H), 7.09 (t, *J* = 2.9 Hz, 1H), 6.29 (dd, *J* = 3.4, 1.6 Hz, 1H); ^13^C NMR (101 MHz, CDCl_3_) δ 138.6, 134.4, 129.7, 127.1, 121.4, 119.9, 116.5, 102.4. The analytical data are consistent with those reported in the literature.^54^

### Benzyl 3-Bromo-1-(phenylsulfonyl)-1*H*-pyrrole-2-carboxylate (**3**)

According to a modified version of the reported procedure,^34^ a 1.6 M solution of *n*-BuLi in cyclohexane (25.0 mL, 40.0 mmol, 1.25 equiv) was slowly added to a precooled −78 °C stirred solution of *i-*Pr_2_NH (5.87 mL, 41.6 mmol, 1.3 equiv) in anhydrous THF (24 mL) under argon atmosphere. After addition was complete, the reaction mixture was stirred for 10 min at −10 °C, then recooled back to −78 °C. A solution of pyrrole derivative **2** (9.15 g, 32.0 mmol, 1 equiv) in THF (30 mL) was added over 20 min at −78 °C. The reaction flask was stirred at the same temperature for 1 h, followed by dropwise addition (∼15 min) of benzyl chloroformate (CbzCl) (8.22 mL, 57.6 mmol, 1.8 equiv) in 10 mL THF. (Note: the traces of CO_2_ from the CbzCl solution in THF were removed with the stream of argon before use.) The reaction mixture was stirred for 30 min at −78 °C, then slowly warmed to 0 °C (∼2 h), quenched with 50 mL of NH_4_Cl_sat_, and diluted with EtOAc (100 mL) and H_2_O (100 mL). The aqueous layer was extracted with EtOAc (2 × 100 mL), dried over Na_2_SO_4_, and concentrated under reduced pressure. The residue was purified by DCVC (2%, then 10% EtOAc/Hept) to give 12.16 g of crude product as an orange oil; impure fractions containing the desired compound were repurified by DCVC (2%, then 10% EtOAc/Hept) and afforded ester **3** (10.94 g, purity >80% by ^1^H NMR) as a pale orange oil which solidified upon storage in the refrigerator.

^1^H NMR (400 MHz, CDCl_3_) δ 7.93–7.88 (m, 2H), 7.64–7.57 (m, 1H), 7.55 (d, *J* = 3.4 Hz, 1H), 7.51–7.45 (m, 2H), 7.41–7.31 (m, 5H), 6.40 (d, *J* = 3.5 Hz, 1H), 5.28 (s, 2H). The analytical data are consistent with those reported in the literature.^34^

### Benzyl 3-Bromo-1*H*-pyrrole-2-carboxylate (**S1**)

A 1 M solution of TBAF in THF (28.6 mL, 28.6 mmol, 1.1 equiv) was added dropwise to a solution of *N*-sulfonylpyrrole **3** (10.94 g, ∼26 mmol, 1 equiv) in dry THF (75 mL) at room temperature. The obtained reaction mixture was stirred for 2 h, and then H_2_O (100 mL) was added. The aqueous layer was extracted with EtOAc (3 × 75 mL); the organic extracts were combined, washed with water (2 × 100 mL), brine (100 mL), dried over Na_2_SO_4_, and concentrated to dryness. Purification by DCVC (5%, then 20% EtOAc/Hept) afforded two fractions containing the product:

1. The less polar fraction (1.32 g) was repurified by column chromatography on SiO_2_ (Reveleris, 0→15% EtOAc/Hept gradient), providing 942 mg of **S1** as a white solid.
2. The more polar fraction (5.87 g) was crystallized from 30 mL of a 20% EtOAc/Hept mixture. The precipitate (1.08 g) was discarded, and the mother liquor was concentrated to give 4.69 g of **S1** as a colorless oil, quickly solidifying upon standing.

The repurified product obtained from both fractions was of analogous purity (^1^H NMR) and equally acceptable for the next chemical step. Total yield −5.64 g (63% over 2 steps from **2**).

^1^H NMR (400 MHz, CDCl_3_) δ 9.35 (br s, 1H), 7.50–7.44 (m, 2H), 7.43–7.31 (m, 3H), 6.85 (t, *J* = 3.1 Hz, 1H), 6.35 (t, *J* = 2.9 Hz, 1H), 5.36 (s, 2H); ^13^C NMR (101 MHz, CDCl_3_) δ 160.1, 135.9, 128.7, 128.3, 122.9, 120.1, 115.1, 104.2, 66.5. The analytical data are consistent with those reported in the literature.^34^

### ((Benzyloxy)carbonyl)((4-(dimethyliminio)pyridin-1(4*H*)-yl)sulfonyl)azanide (**7**)

A solution of benzyl alcohol (6.28 mL, 60.6 mmol, 1.0 equiv) in CH_2_Cl_2_ (100 mL) was cooled to 0 °C followed by the dropwise addition of chlorosulfonyl isocyanate (5.21 mL, 60 mmol, 1.0 equiv). After stirring at 0 °C for 10 min, 4-(dimethylamino)pyridine (14.7 g, 120 mmol, 2 equiv) was added portionwise, and the reaction mixture allowed to warm to room temperature and stirred overnight. The resulting mixture was diluted with CH_2_Cl_2_ (100 mL), washed with water (3 × 100 mL), dried over MgSO_4_, filtered, and concentrated to dryness under reduced pressure to give the desired product as a white solid (18.0 g, 90%).

^1^H NMR (400 MHz, DMSO-*d*_6_) δ 8.51–8.43 (m, 2H), 7.37–7.27 (m, 3H), 7.27–7.22 (m, 2H), 6.98–6.90 (m, 2H), 4.87 (s, 2H), 3.22 (s, 6H); ^13^C NMR (101 MHz, DMSO-*d*_6_) δ 157.5, 156.6, 138.4, 136.9, 128.2, 127.6, 127.6, 106.3, 65.9, 40.0. The analytical data are consistent with those reported in the literature.^55^

### Benzyl 1-(*N*-((Benzyloxy)carbonyl)sulfamoyl)-3-bromo-1*H*-pyrrole-2-carboxylate (**4**)

Sodium hydride (60% in mineral oil, 822 mg, 20.6 mmol, 1.5 equiv) was added portionwise to a precooled 0 °C solution of benzyl 3-bromo-1-pyrrole-2-carboxylate (**S1**) (3.84 g, 13.7 mmol, 1 equiv) in dry THF (41 mL). After stirring at 0 °C for 30 min, sulfamoylating reagent **7** (5.06 g, 15.1 mmol, 1.1 equiv) was added, and the reaction mixture was heated to reflux for 4 h. After cooling to 0 °C, the reaction was quenched by the dropwise addition of water (40 mL), concentrated under reduced pressure to remove organic solvents, and extracted into ethyl acetate (3 × 100 mL). The combined organic phases were washed with brine (40 mL), dried over Na_2_SO_4_, and concentrated to dryness under reduced pressure. The residue was purified by column chromatography on SiO_2_ (Reveleris, 20→100% EtOAc/Hept, then 0→20% MeOH/EtOAc gradients) to give 7.03 g of the product **4** as the sodium salt as a beige foam. The residue was taken up in EtOAc (40 mL), sequentially washed with 0.5 M HCl_aq_ (2 × 40 mL), brine (40 mL), dried over Na_2_SO_4_, and evaporated to dryness *in vacuo* and purified by column chromatography (Reveleris, 10→50% EtOAc/heptane gradient) to afford 4.68 g (69%) of the desired compound **4** as a yellowish oil.

^1^H NMR (400 MHz, CDCl_3_) δ 7.50–7.44 (m, 3H), 7.41–7.24 (m, 9H), 6.32 (d, *J* = 3.4 Hz, 1H), 5.35 (s, 2H), 5.14 (s, 2H); ^13^C NMR (101 MHz, CDCl_3_) δ 159.5, 149.6, 134.7, 134.1, 129.7, 129.1, 128.9, 128.8, 128.8, 128.7, 128.6, 121.6, 114.9, 112.4, 69.5, 68.0. The analytical data are consistent with those reported in the literature.^34^

### General Procedure A: Suzuki–Miyaura Cross-Coupling Reaction

A microwave vial charged with bromide **4** (197 mg, 0.40 mmol, 1 equiv), the corresponding boronate (0.52 mmol, 1.3 equiv), Na_2_CO_3_ (127 mg, 1.20 mmol, 3 equiv), Pd(dppf)Cl_2_·DCM (16.3 mg, 0.02 mmol, 0.05 equiv) and a degassed dioxane/H_2_O mixture (2:1, 2 mL) was purged with argon, sealed, and stirred under microwave irradiation at 100 °C for 3 h. After cooling to rt, the reaction mixture was diluted with 2 mL of EtOAc and 2 mL of H_2_O; the organic phase was separated, and the aqueous layer was extracted with EtOAc (2 × 2 mL). The combined organic phases were filtered through a pad of Na_2_SO_4_ with Celite on top, and the filtrate was concentrated under reduced pressure and further purified by column chromatography on SiO_2_ (Reveleris purification system) to afford the desired C3 substituted pyrrole.

### Sodium ((Benzyloxy)carbonyl)((2-((benzyloxy)carbonyl)-3-(4-fluorophenyl)-1*H*-pyrrol-1-yl)sulfonyl)azanide (**5**)

Use of General Procedure A with 4-fluorophenyl)boronic acid as the coupling partner gave, after purification by column chromatography (Reveleris, 20→90% EtOAc/Hept gradient), the desired compound **5** as a yellow amorphous solid in 70% yield (149 mg).

^1^H NMR (600 MHz, CDCl_3_) δ 7.55 (d, *J* = 3.1 Hz, 1H), 7.13 (t, *J* = 7.4 Hz, 1H), 7.10–7.03 (m, 5H), 7.00 (t, *J* = 7.6 Hz, 2H), 6.86 (dd, *J* = 8.4, 5.3 Hz, 2H), 6.69–6.61 (m, 4H), 5.84 (d, *J* = 3.1 Hz, 1H), 4.89 (s, 2H), 4.81 (s, 2H); ^13^C NMR (151 MHz, CDCl_3_) δ 162.2 (d, *J* = 246.5 Hz), 161.8, 158.4 (br), 136.5, 136.1, 134.5, 131.2 (d, *J* = 3.3 Hz), 130.9 (d, *J* = 8.1 Hz), 129.7, 128.44, 128.40, 128.3, 128.1, 127.9, 127.8, 118.9, 114.6 (d, *J* = 21.5 Hz), 110.9, 67.9, 67.2; ^19^F NMR (376 MHz, CDCl_3_) δ −115.4; LRMS (ESI) *m*/*z*: [M-Na]^−^ calcd for C_26_H_20_FN_2_NaO_6_S 507.1, found 507.2.

### General Procedure B: Hydrogenolysis of *N*-Cbz and *O*-Bn Protected Pyrrole-2-carboxylates

A one-necked round-bottom flask containing a 0.025 M methanol solution of the corresponding *O*-Bn- and *N*-Cbz-protected pyrrole (1 equiv) was charged with Pd on carbon (10% w/w, 0.2 equiv), sealed, and evacuated/backfilled with dihydrogen gas (3 times). The reaction mixture was hydrogenated at atmospheric pressure (H_2_ balloon) overnight under vigorous stirring, then filtered through a pad of Celite, and the filter cake was washed with methanol. The combined filtrates were concentrated to dryness under reduced pressure.

### 3-(4-Fluorophenyl)-1-sulfamoyl-1*H*-pyrrole-2-carboxylic acid (**6a**)

The *N*-Cbz sodium salt **5** (72.7 mg, 0.137 mmol) was dissolved in 10 mL of EtOAc, transferred to a separatory funnel, and successively washed with 1 M HCl_aq_ (2 × 5 mL), brine (5 mL), and dried over Na_2_SO_4_. The solvent was removed *in vacuo* to give **S8** as a yellow oil in 96% yield (67.2 mg).

^1^H NMR (600 MHz, CDCl_3_) δ 8.69 (br s, 1H), 7.55 (d, *J* = 3.3 Hz, 1H), 7.38–7.34 (m, 3H), 7.34–7.28 (m, 3H), 7.27–7.22 (m, 4H, overlapped with solvent peak), 7.04–7.00 (m, 2H), 6.94–6.87 (m, 2H), 6.23 (d, *J* = 3.3 Hz, 1H), 5.18 (s, 2H), 5.12 (s, 2H); ^19^F NMR (376 MHz, CDCl_3_) δ −114.1.

The oil obtained above (67.2 mg) was hydrogenated according to General Procedure B; then, the crude residue was further purified by prepHPLC to give the desired compound **6a** in 72% yield (27.0 mg) as a white fluffy solid.

^1^H NMR (600 MHz, DMSO-*d*_6_) δ 13.17 (br s, 1H), 8.17 (br s, 2H), 7.46–7.42 (m, 2H), 7.42 (d, *J* = 3.2 Hz, 1H), 7.24–7.18 (m, 2H), 6.37 (d, *J* = 3.2 Hz, 1H); ^13^C NMR (151 MHz, DMSO-*d*_6_) δ 162.5, 161.5 (d, *J* = 244.1 Hz), 131.7, 130.75 (d, *J* = 3.4 Hz), 130.69 (d, *J* = 8.2 Hz), 125.2, 120.8, 114.8 (d, *J* = 21.4 Hz), 110.7; ^19^F NMR (376 MHz, DMSO-*d*_6_) δ −115.8; HPLC analysis t_R_ 11.3 min, purity >99.6%; HRMS (ESI) *m*/*z*: [M-H]^−^ calcd for C_11_H_8_FN_2_O_4_S 283.0194, found 283.0189.

## Supporting Information Available

Synthesis and characterization of compounds and information on enzymatic and microbiological assays and crystallography (PDF)
