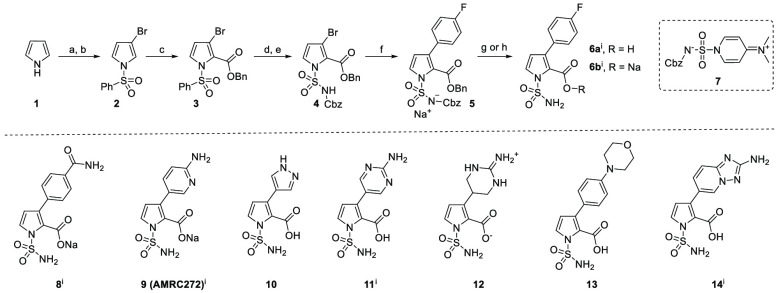# Correction to “Structural Basis of Metallo-β-lactamase Inhibition by *N*-Sulfamoylpyrrole-2-carboxylates”

**DOI:** 10.1021/acsinfecdis.1c00472

**Published:** 2021-10-04

**Authors:** Alistair J. M. Farley, Yuri Ermolovich, Karina Calvopiña, Patrick Rabe, Tharindi Panduwawala, Jürgen Brem, Fredrik Björkling, Christopher J. Schofield

In Scheme 1, in the footnote, (i) should be changed from “structure previously disclosed” to “structure and synthesis previously disclosed.^34^”

Scheme 1 should also be changed to include superscript “i” after **6a** and **6b**. Here is the updated graphic for Scheme 1:

**Scheme 1:**