# Supplementary material for: Structural Basis of Metallo-β-lactamase Inhibition by N-Sulfamoylpyrrole-2-carboxylates
Source: ACS Infect Dis. 2021 May 18;7(6):1809–17. doi: 10.1021/acsinfecdis.1c00104 (PMC8205225; doi:10.1021/acsinfecdis.1c00104)
Supplement: Supplementary file 1 — id1c00104_si_001.pdf [file id1c00104_si_001.pdf]

Supporting Information for

Structural basis of metallo-β-lactamase inhibition by N-sulfamoylpyrrole-2-carboxylates

Alistair J. M. Farley[1§], Yuri Ermolovich[2§], Karina Calvopiña[1], Patrick Rabe[1], Tharindi Panduwawala,[1] Jürgen Brem[1], Fredrik Björkling[2]*, Christopher J. Schofield[1]*

[1]*Department of Chemistry, Chemistry Research Laboratory and the Ineos Institute for Antimicrobial Research, University of Oxford, 12 Mansfield Road,Oxford, OX1 3TA, UK.*

[2]*Department of Drug Design and Pharmacology, Faculty of Health and Medical Sciences, University of Copenhagen, DK-2100 Copenhagen Ø, Denmark*

[§] The contributions of these authors should be considered equal.

*fb@sund.ku.dk; *christopher.schofield@chem.ox.ac.uk

Funding declaration

We thank the Innovative Medicines Initiative (European Lead factory and ENABLE components), the Medical Research Council and the Wellcome Trust, and Cancer Research UK and the Ineos Institute for Antimicrobial Research for funding our work on antibiotics, MBL fold / metallo-enzymes, and β-lactamase inhibitors. This research was funded in whole, or in part, by the Wellcome Trust [Grant number 106244/Z/14/Z]. For the purpose of open access, the author has applied a CC BY public copyright licence to any Author Accepted Manuscript version arising from this submission.

Table of contents

1 Compound Synthesis 2

1.1 General information 2

1.2 Synthesis and characterization of compounds 3

2 Biochemical Assays 12

3 Crystallography 13

4 References 15

5 NMR spectra 17

6 HPLC chromatograms of final compounds 41

1 Compound Synthesis

1.1 General information

Commercially available reagents and solvents were from Merck or Fluorochem and were used as received. All manipulations with air- and moisture-sensitive compounds were carried out under a positive pressure of argon in flame-dried glassware. Reactions under microwave conditions were carried out in Biotage® Initiator EXP microwave reactor with Robot Sixty sample processor.

Chromatographic separation/purification was performed either using manually packed columns with Silica gel 60 (Merck, 15 – 40 µm) for dry column vacuum chromatography (DCVC)) or using Reveleris® X2 Flash Chromatography Purification System (BÜCHI) with FlashPure Silica pre-packed columns. Reactions were monitored by TLC on silica gel 60 F254 plates (Merck).

NMR-spectra were acquired using 600 MHz Bruker Avance III HD machine equipped with 5 mm DCH cryoprobe and 400 MHz Bruker Avance II equipped with a 5 mm BBFO probe. Chemical shifts were referenced to residual protio- and perdeuterio-solvent resonances (δ_H 7.26 and δ_C 77.16 for $CDCl_3$; δ_H 2.50 and δ_C 39.52 for DMSO-d_6) as internal standards for ^{1}H NMR and ^{13}C NMR spectra, respectively. ^{19}F NMR spectra were referenced indirectly via the ^{2}H signal of the lock substance ($CDCl_3$ or DMSO-d_6) and the $\Xi(^{19}F)$ value. All NMR spectra were processed with MestReNova software v. 14.1.

LRMS machine (low resolution mass spectrometry) data were obtained using a Waters Acquity H-class UPLC with a Sample Manager FTN and a TUV dual wavelength detector coupled to a QDa single quadrupole analyzer using electrospray ionization (ESI). UPLC separation was achieved with a C18 reversed-phase column (Acquity UPLC BEH C18, 2.1 mm × 50 mm, 1.7 µm) operated at 40 °C, using a linear gradient of the binary solvent system of buffer A (H_2O:MeCN:formic acid, 95:5:0.1 v/v/v%) to buffer B (MeCN:formic acid, 100:0.1 v/v%) from 0 to 100% B in 3.5 min, then 1 min at 100% B, maintaining a flow rate of 0.8 mL/min. High resolution mass spectra were recorded using a Bruker µTOF (ESI) spectrometer. The m/z values are reported in Daltons.

Analytical HPLC was carried out on an Ultimate HPLC system (Thermo Scientific) consisting of a LPG-3400A pump (1 mL/min), a WPS-3000SL autosampler and a DAD-3000D diode array detector (220 and 254 nm) using a Gemini-NX C18 column (4.6 × 250 mm, 3 µm, 110 Å, Phenomenex); gradient elution 0 to 100% B (MeCN-H_2O-TFA 90:10:0.1 v/v/v%) in solvent A (H_2O-TFA 100:0.1 v/v%) over 15 min.

Preparative HPLC (prepHPLC) was carried out on an Ultimate HPLC system (Thermo Scientific) consisting of a LPG-3200BX pump (20 mL/min), a Rheodyne 9725i injector, a 10 mL loop, a MWD-300SD detector (220 and 254 nm) and an AFC-3000SD automated fraction collector using a Gemini-NX C18 column (21.2 × 250 mm, 5 µm, 110 Å, Phenomenex); gradient elution 0 to 100% B (MeCN-H_2O-formic acid 90:10:0.1 v/v/v%) in solvent A (H_2O-formic acid 100:0.1 v/v%) over 15 min (unless noted otherwise).

Data for both analytical and preparative HPLC were acquired and processed using Chromeleon software v. 6.80.

1.2 Synthesis and characterization of compounds

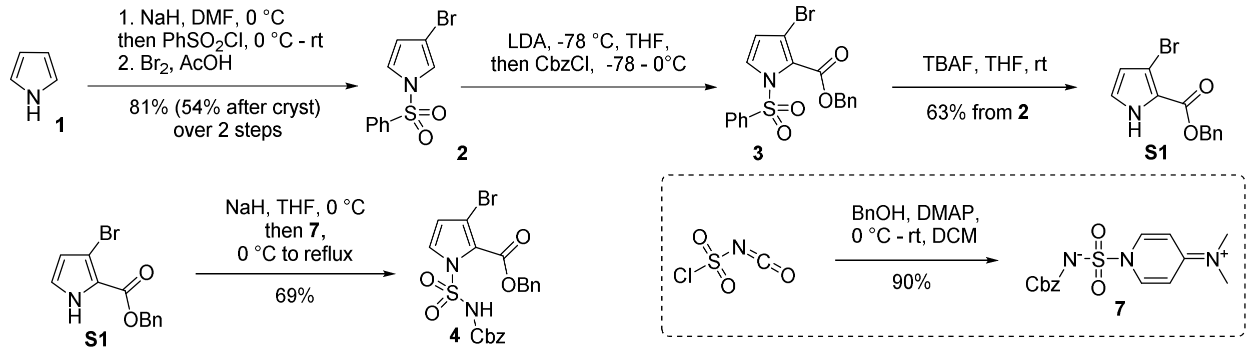

Scheme S1. Synthesis of common bromopyrrole intermediate 4.

3-Bromo-1-(phenylsulfonyl)-1*H*-pyrrole (2)

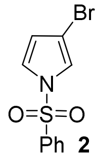

N1-Sulfonylation.[1] Sodium hydride (2.20 g, 55.0 mmol, 60 wt. %, 1.1 equiv) was added portionwise to the solution of pyrrole (3.47 mL, 50.0 mmol, 1.0 equiv) in dry DMF (150 mL) at 0 °C. The obtained mixture was stirred for 1 h at the same temperature (*Note for this step*: a constant flow of nitrogen gas was used to reduce foaming). Benzenesulfonyl chloride (7.66 mL, 66.0 mmol, 1.2 equiv) was added slowly over 5 min at 0 °C, the cooling bath was removed, and the reaction was further stirred for 0.5 h at room temperature (starting pyrrole was consumed, TLC). The reaction mixture was carefully quenched with half-saturated NH_4Cl (200 mL) at 0 °C and diluted with 200 mL EtOAc. The organic phase was washed with water (4×150 mL), brine (150 mL), dried over Na_2SO_4 and concentrated under reduced pressure providing 10.64 g of crude 1-(phenylsulfonyl)-1*H*-pyrrole as a beige solid which was taken through to the next step without further purification.

Bromination.[2] A solution of bromine (2.57 mL, 50 mmol, 1 equiv) in acetic acid (40 mL) was added dropwise to the solution of 1-(phenylsulfonyl)-1*H*-pyrrole (10.64 g, ~50 mmol, 1 equiv) in AcOH (90 mL). The mixture was refluxed for 1 h, then cooled to rt, concentrated and co-evaporated with toluene (2×150 mL). Purification by DCVC (5% EtOAc - heptane) afforded 11.53 g of the desired product as a purple oil which solidified upon standing. Further purification by crystallization from MeOH (20 mL) gave 7.69 g (54% from pyrrole) of **2** as a white crystalline solid.

^{1}H NMR (400 MHz, $CDCl_3$) δ 7.90 – 7.83 (m, 2H), 7.67 – 7.59 (m, 1H), 7.58 – 7.46 (m, 2H), 7.19 – 7.15 (m, 1H), 7.09 (t, J = 2.9 Hz, 1H), 6.29 (dd, J = 3.4, 1.6 Hz, 1H); **^{13}C NMR** (101 MHz, $CDCl_3$) δ 138.6, 134.4, 129.7, 127.1, 121.4, 119.9, 116.5, 102.4. The analytical data are consistent with those reported in the literature.[2]

Benzyl 3-bromo-1-(phenylsulfonyl)-1*H*-pyrrole-2-carboxylate (3)

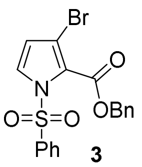

According to a modified version of the reported procedure,[3] a 1.6 M solution of *n*-BuLi in cyclohexane (25.0 mL, 40.0 mmol, 1.25 equiv) was slowly added to a precooled to -78 °C stirred solution of *i*-Pr_2NH (5.87 mL, 41.6 mmol, 1.3 equiv) in anhydrous THF (24 mL) under argon atmosphere. After addition was complete, the reaction mixture was stirred for 10 min at -10 °C, then recooled back to -78 °C. A solution of pyrrole derivative **2** (9.15 g, 32.0 mmol, 1 equiv) in THF (30 mL) was added over 20 min at -78 °C. The reaction flask was stirred at the same temperature for 1 h, followed by

dropwise addition (~15 min) of benzyl chloroformate (CbzCl) (8.22 mL, 57.6 mmol, 1.8 equiv) in 10 mL THF. (*Note*: the traces of CO_2 from the CbzCl solution in THF were removed with the stream of argon before use). The reaction mixture was stirred for 30 min at -78 °C, then slowly warmed to 0 °C (~2 h), quenched with 50 mL NH_4Cl_{sat} and diluted with EtOAc (100 mL) and H_2O (100 mL). The aqueous layer was extracted with EtOAc (2×100 mL), dried over Na_2SO_4 and concentrated under reduced pressure. The residue was purified by DCVC (2%, then 10% EtOAc – Hept) to give 12.16 g of crude product as an orange oil; impure fractions containing the desired compound were repurified by DCVC (2%, then 10% EtOAc – Hept) and afforded ester **3** (10.94 g, purity >80% by ^{1}H NMR) as a pale orange oil which solidified upon storage in the refrigerator.

^{1}H NMR (400 MHz, $CDCl_3$) δ 7.93 – 7.88 (m, 2H), 7.64 – 7.57 (m, 1H), 7.55 (d, *J* = 3.4 Hz, 1H), 7.51 – 7.45 (m, 2H), 7.41 – 7.31 (m, 5H), 6.40 (d, *J* = 3.5 Hz, 1H), 5.28 (s, 2H). The analytical data are consistent with those reported in the literature.[3]

Benzyl 3-bromo-1*H*-pyrrole-2-carboxylate (S1)[3]

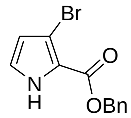

S1

A 1 M solution of TBAF in THF (28.6 mL, 28.6 mmol, 1.1 equiv) was added dropwise to a solution of *N*-sulfonylpyrrole **3** (10.94 g, ~26 mmol, 1 equiv) in dry THF (75 mL) at room temperature. The obtained reaction mixture was stirred for 2 h, then H_2O (100 mL) was added. The aqueous layer was extracted with EtOAc (3×75 mL); the organic extracts were combined, washed with water (2×100 mL), brine (100 mL), dried over Na_2SO_4 and concentrated to dryness. Purification by DCVC (5%, then 20% EtOAc – Hept) afforded two fractions containing the product:

1. *Less polar fraction* (1.32 g) was repurified by column chromatography on SiO_2 (Reveleris®, 0→15% EtOAc – Hept gradient) providing 942 mg of **S1** as a white solid;
2. *More polar fraction* (5.87 g) was crystallized from 30 mL 20% EtOAc – Hept mixture. The precipitate (1.08 g) was discarded, the mother liquor was concentrated to give 4.69 g of **S1** as a colorless oil, quickly solidifying upon standing.

The repurified product obtained from both fractions was of analogous purity (^{1}H NMR) and equally acceptable for the next chemical step. Total yield – 5.64 g (63% over 2 steps from **2**).

^{1}H NMR (400 MHz, $CDCl_3$) δ 9.35 (br s, 1H), 7.50 – 7.44 (m, 2H), 7.43 – 7.31 (m, 3H), 6.85 (t, *J* = 3.1 Hz, 1H), 6.35 (t, *J* = 2.9 Hz, 1H), 5.36 (s, 2H); **^{13}C NMR** (101 MHz, $CDCl_3$) δ 160.1, 135.9, 128.7, 128.3, 122.9, 120.1, 115.1, 104.2, 66.5. The analytical data are consistent with those reported in the literature. [3]

((Benzyloxy)carbonyl)((4-(dimethyliminio)pyridin-1(4*H*)-yl)sulfonyl)azanide (7)

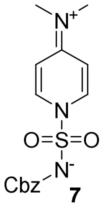

7

A solution of benzyl alcohol (6.28 ml, 60.6 mmol, 1.0 equiv) in CH_2Cl_2 (100 ml) was cooled to 0 °C followed by the dropwise addition of chlorosulfonyl isocyanate (5.21 ml, 60 mmol, 1.0 equiv). After stirring at 0 °C for 10 minutes, 4-(dimethylamino)pyridine (14.7 g, 120 mmol, 2 equiv) was added portionwise, and the reaction mixture allowed to warm to room temperature and stirred overnight. The resulting mixture was diluted with CH_2Cl_2 (100 ml), washed with water (3×100 ml), dried over $MgSO_4$, filtered and concentrated to dryness under reduced pressure to give the desired product as a white solid (18.0 g, 90%).

^{1}H NMR (400 MHz, DMSO-d_6) δ 8.51 – 8.43 (m, 2H), 7.37 – 7.27 (m, 3H), 7.27 – 7.22 (m, 2H), 6.98 – 6.90 (m, 2H), 4.87 (s, 2H), 3.22 (s, 6H); **^{13}C NMR** (101 MHz, DMSO-d_6) δ 157.5,

156.6, 138.4, 136.9, 128.2, 127.6, 127.6, 106.3, 65.9, 40.0. The analytical data are consistent with those reported in the literature.[4]

Benzyl 1-(*N*-((benzyloxy)carbonyl)sulfamoyl)-3-bromo-1*H*-pyrrole-2-carboxylate (4)[3]

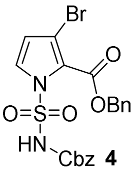

Sodium hydride (60% in mineral oil, 822 mg, 20.6 mmol, 1.5 equiv) was added portionwise to a precooled to 0 °C solution of benzyl 3-bromo-1-pyrrole-2-carboxylate (**S1**) (3.84 g, 13.7 mmol, 1 equiv) in dry THF (41 mL). After stirring at 0 °C for 30 min, sulfamoylating reagent **7** (5.06 g, 15.1 mmol, 1.1 equiv) was added, and the reaction mixture heated to reflux for 4 hours. After cooling to 0 °C, the reaction was quenched by the dropwise addition of water (40 ml), concentrated under reduced pressure to remove organic solvents and extracted into ethyl acetate (3×100 ml). The combined organic phases were washed with brine (40 ml), dried over Na_2SO_4 and concentrated to dryness under reduced pressure. The residue was purified by column chromatography on SiO_2 (Reveleris®, 20→100% EtOAc – Hept, then 0→20% MeOH – EtOAc gradients) to give 7.03 g of the product **4** as the sodium salt as a beige foam. The residue was taken up in EtOAc (40 mL), sequentially washed with 0.5 M HCl_{aq} (2×40 mL), brine (40 ml), dried over Na_2SO_4 and evaporated to dryness *in vacuo* and purified by column chromatography (Reveleris®, 10→50% EtOAc – heptane gradient) to afford 4.68 g (69%) of the desired compound **4** as a yellowish oil.

^{1}H NMR (400 MHz, $CDCl_3$) δ 7.50 – 7.44 (m, 3H), 7.41 – 7.24 (m, 9H), 6.32 (d, *J* = 3.4 Hz, 1H), 5.35 (s, 2H), 5.14 (s, 2H); **^{13}C NMR** (101 MHz, $CDCl_3$) δ 159.5, 149.6, 134.7, 134.1, 129.7, 129.1, 128.9, 128.8, 128.8, 128.7, 128.6, 121.6, 114.9, 112.4, 69.5, 68.0. The analytical data are consistent with those reported in the literature.[3]

General Procedure A – Suzuki-Miyaura cross-coupling reaction

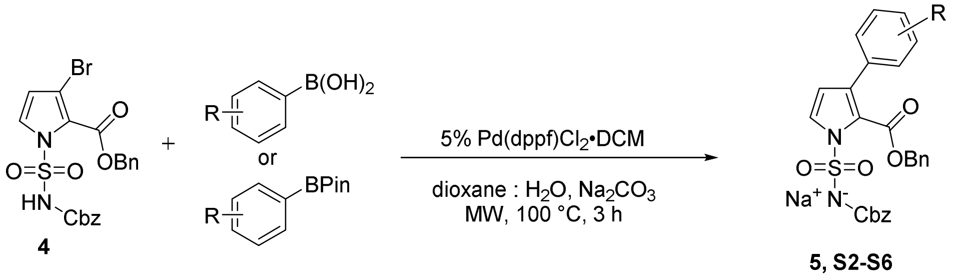

A microwave vial charged with bromide **4** (197 mg, 0.40 mmol, 1 equiv), the corresponding boronate (0.52 mmol, 1.3 equiv), Na_2CO_3 (127 mg, 1.20 mmol, 3 equiv), Pd(dppf)Cl_2•DCM (16.3 mg, 0.02 mmol, 0.05 equiv) and degassed dioxane – H_2O mixture (2:1, 2 mL) was purged with argon, sealed and stirred under microwave irradiation at 100 °C for 3 h. After cooling to rt, the reaction mixture was diluted with 2 mL EtOAc and 2 mL H_2O; the organic phase was separated; the aqueous layer was extracted with EtOAc (2×2 mL). The combined organic phases were filtered through a pad of Na_2SO_4 with Celite® on top and the filtrate was concentrated under reduced pressure and further purified by column chromatography on SiO_2 (Reveleris® purification system) to afford the desired C3 substituted pyrrole.

Sodium ((benzyloxy)carbonyl)((2-((benzyloxy)carbonyl)-3-(4-fluorophenyl)-1*H*-pyrrol-1-yl)sulfonyl)azanide (5)

5

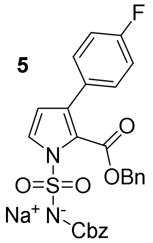

Use of General Procedure A with 4-fluorophenyl)boronic acid as the coupling partner gave, after purification by column chromatography (Reveleris®, 20→90% EtOAc – Hept gradient), the desired compound **5** as a yellow amorphous solid in 70% yield (149 mg).

^{1}H NMR (600 MHz, $CDCl_3$) δ 7.55 (d, *J* = 3.1 Hz, 1H), 7.13 (t, *J* = 7.4 Hz, 1H), 7.10 – 7.03 (m, 5H), 7.00 (t, *J* = 7.6 Hz, 2H), 6.86 (dd, *J* = 8.4, 5.3 Hz, 2H), 6.69 – 6.61 (m, 4H), 5.84 (d, *J* = 3.1 Hz, 1H), 4.89 (s, 2H), 4.81 (s, 2H); **^{13}C NMR** (151 MHz, $CDCl_3$) δ 162.2 (d, *J* = 246.5 Hz), 161.8, 158.4 (br), 136.5, 136.1, 134.5, 131.2 (d, *J* = 3.3 Hz), 130.9 (d, *J* = 8.1 Hz), 129.7, 128.44, 128.40, 128.3, 128.1, 127.9, 127.8, 118.9, 114.6 (d, *J* = 21.5 Hz), 110.9, 67.9, 67.2; **^{19}F NMR** (376 MHz, $CDCl_3$) δ -115.4; **LRMS** (ESI) *m/z*: $[M\text{-}Na]^-$ calcd for $C_{26}H_{20}FN_2NaO_6S$ 507.1, found 507.2.

Sodium ((benzyloxy)carbonyl)((2-((benzyloxy)carbonyl)-3-(4-carbamoylphenyl)-1*H*-pyrrol-1-yl)sulfonyl)azanide (**S2**)

S2

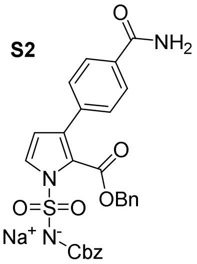

Use of General Procedure A with 4-(4,4,5,5-tetramethyl-1,3,2-dioxaborolan-2-yl)benzamide as the coupling partner gave, after purification by column chromatography (Reveleris®, 0→20% CH_2Cl_2 – MeOH gradient), the desired compound **S2** as a beige solid in 72% yield (159 mg).

^{1}H NMR (600 MHz, DMSO-*d*$_6$) δ 7.93 (br s, 1H), 7.81 – 7.78 (m, 2H), 7.40 – 7.35 (m, 4H), 7.33 – 7.25 (m, 10H), 6.30 (d, *J* = 3.1 Hz, 1H), 5.15 (s, 2H), 4.87 (s, 2H); **^{13}C NMR** (151 MHz, DMSO-*d*$_6$) δ 167.6, 162.4, 157.3 (br), 137.7, 137.3, 135.6, 132.2, 128.2, 128.13, 128.09, 127.8, 127.6, 127.4, 127.3, 127.0, 126.1 (br), 124.8 (br), 120.7, 107.3, 66.6, 65.4; **LRMS** (ESI) *m/z*: $[M\text{+}2H\text{-}Na]^+$ calcd for $C_{27}H_{24}N_3O_7S$ 534.1, found 534.4; $[M\text{-}Na]^-$ calcd for $C_{27}H_{22}N_3NaO_7S$ 532.1, found 532.1.

Sodium ((3-(6-aminopyridin-3-yl)-2-((benzyloxy)carbonyl)-1*H*-pyrrol-1-yl)sulfonyl)-((benzyloxy)carbonyl)azanide (S3)

S3

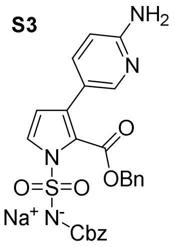

Use of General Procedure A with 5-(4,4,5,5-tetramethyl-1,3,2-dioxaborolan-2-yl)pyridin-2-amine as the coupling partner gave, following purification by column chromatography (Reveleris®, 0→20% CH_2Cl_2– MeOH gradient), the desired compound **S3** as a brown amorphous solid (film) in 70% yield (147 mg).

^{1}H NMR (600 MHz, DMSO-*d*$_6$) δ 7.94 (d, *J* = 2.3 Hz, 1H), 7.66 (dd, *J* = 8.9, 2.3 Hz, 1H), 7.44 – 7.39 (m, 2H), 7.33 – 7.25 (m, 9H), 7.14 (br s, 2H), 6.70 (d, *J* = 8.9 Hz, 1H), 6.19 (d, *J* = 3.1 Hz, 1H), 5.14 (s, 2H), 4.85 (s, 2H); **^{13}C NMR** (151 MHz, DMSO-*d*$_6$) δ 161.8, 157.6, 154.8 (br), 141.3 (br), 138.5 (br), 137.9, 135.7, 128.2, 128.1, 128.1, 127.7, 127.4, 127.3, 125.9, 123.9 (br), 120.0, 119.2, 110.8 (br), 107.0, 66.4, 65.3; **LRMS** (ESI) *m/z*: $[M\text{+}2H\text{-}Na]^+$ calcd for $C_{25}H_{21}N_4NaO_6S$ 507.1, found 507.3; $[M\text{-}Na]^-$ calcd 505.1, found 505.1. The analytical data are consistent with those reported in the literature.[3]

Sodium ((benzyloxy)carbonyl)((2-((benzyloxy)carbonyl)-3-(1*H*-pyrazol-4-yl)-1*H*-pyrrol-1-yl)sulfonyl)azanide (S4)

S4

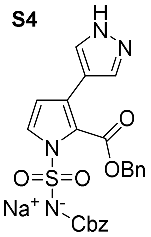

Use of General Procedure A with 4-(4,4,5,5-tetramethyl-1,3,2-dioxaborolan-2-yl)-1*H*-pyrazole as the coupling partner gave, after purification by column chromatography (Reveleris®, 0→20% CH_2Cl_2 – MeOH gradient), the desired compound **S4** as a beige solid in 26% yield (52.3 mg).

^{1}H NMR (600 MHz, DMSO-d_6) δ 12.74 (br s, 1H), 7.87 – 7.58 (m, 2H, contains 7.79 (br s,1H) and 7.64 (br s,1H)), 7.51 – 7.47 (m, 2H), 7.33 – 7.23 (m, 9H), 6.21 (d, *J* = 3.0 Hz, 1H), 5.19 (s, 2H), 4.84 (s, 2H); **^{13}C NMR** (151 MHz, DMSO-d_6) δ 162.5, 157.7, 138.0, 137.5 (br), 136.1, 128.14, 128.10, 128.06, 127.6, 127.4, 127.2, 126.2 (br), 125.8, 120.5, 118.9, 114.1, 106.8, 66.2, 65.2; **LRMS** (ESI) *m/z*: $[M+2H-Na]^+$ calcd for $C_{23}H_{19}N_4NaO_6S$ 481.1, found 481.2; $[M-Na]^-$ calcd 479.1, found 479.1.

Sodium ((3-(2-aminopyrimidin-5-yl)-2-((benzyloxy)carbonyl)-1*H*-pyrrol-1-yl)sulfonyl)((benzyloxy)carbonyl)azanide (S5)

S5

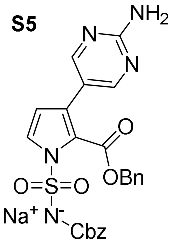

Use of General Procedure A with 5-(4,4,5,5-tetramethyl-1,3,2-dioxaborolan-2-yl)pyrimidin-2-amine as the coupling partner gave, following purification by column chromatography (Reveleris®, 0→10% CH_2Cl_2 – MeOH gradient), the desired compound **S5** as a light brown solid in 77% yield (163 mg).

^{1}H NMR (600 MHz, DMSO-d_6) δ 8.24 (s, 2H), 7.44 – 7.40 (m, 2H), 7.33 – 7.24 (m, 9H), 6.66 (br s, 2H), 6.19 (d, *J* = 3.1 Hz, 1H), 5.13 (s, 2H), 4.86 (s, 2H); **^{13}C NMR** (151 MHz, DMSO-d_6) δ 162.2, 161.8, 157.5 (br), 156.7, 137.9, 135.8, 128.1, 128.1, 128.0, 127.7, 127.4, 127.3, 126.2, 123.5 (br), 119.7, 117.1, 107.0, 66.3, 65.3; **LRMS** (ESI) *m/z*: $[M+2H-Na]^+$ calcd for $C_{24}H_{20}N_5NaO_6S$ 508.1, found 508.4; $[M-Na]^-$ calcd 506.1, found 506.1.

Sodium ((benzyloxy)carbonyl)((2-((benzyloxy)carbonyl)-3-(4-morpholinophenyl)-1*H*-pyrrol-1-yl)sulfonyl)azanide (S6)

S6

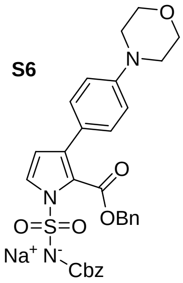

Use of General Procedure A with (4-morpholinophenyl)boronic acid as the coupling partner gave, following purification by column chromatography (Reveleris®, 0→10% CH_2Cl_2 – MeOH gradient), the desired compound **S6** as a beige solid in 88% yield (211 mg).

^{1}H NMR (600 MHz, DMSO-d_6) δ 7.38 – 7.35 (m, 2H), 7.34 – 7.25 (m, 9H), 7.23 – 7.19 (m, 2H), 6.88 – 6.84 (m, 2H), 6.19 (d, *J* = 3.1 Hz, 1H), 5.14 (s, 2H), 4.89 (s, 2H), 3.76 – 3.72 (m, 4H), 3.12 – 3.08 (m, 4H); **^{13}C NMR** (151 MHz, DMSO-d_6) δ 162.5, 156.8 (br), 149.7, 137.6, 135.7, 128.3, 128.2, 128.2, 128.1, 127.8, 127.5, 127.4, 125.1, 125.0 (br), 119.5, 114.8, 107.5 (br), 66.4, 66.1, 65.6, 48.3; **LRMS** (ESI) *m/z*: $[M+2H-Na]^+$ calcd for $C_{30}H_{28}N_3NaO_7S$ 576.2, found 576.4; $[M-Na]^-$ calcd 574.1, found 574.3.

Potassium ((3-(2-amino-[1,2,4]triazolo[1,5-a]pyridin-6-yl)-2-((benzyloxy)carbonyl)-1*H*-pyrrol-1-yl)sulfonyl)-((benzyloxy)carbonyl)azanide (S7)

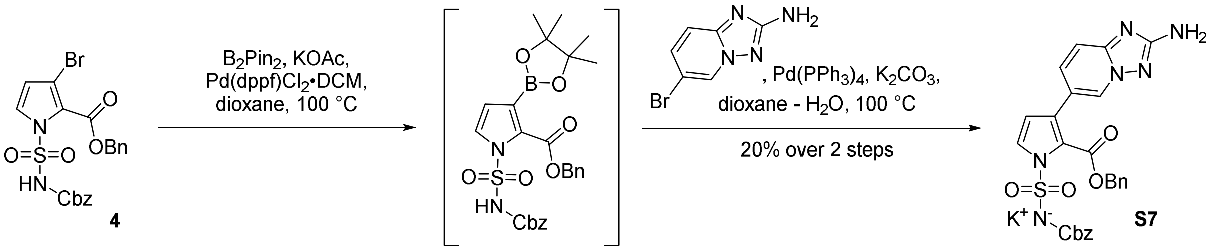

Step 1, borylation. A microwave vial with stirring bar charged with aryl bromide **4** (493 mg, 1 mmol, 1 equiv), bis(pinacolato)diboron (279 mg, 1.1 mmol, 1.1 equiv), KOAc (294 mg, 3.0 mmol, 3 equiv), $Pd(dppf)Cl_2$•DCM (40.8 mg, 0.05 mmol, 5 mol%) and degassed dioxane (4 mL) was purged with argon, then sealed and stirred at 100 °C overnight. After being cooled to rt, the reaction mixture was diluted with 4 mL EtOAc and 4 mL H_2O. The organic phase was separated; the aqueous layer was extracted with EtOAc (2×4 mL). Combined organic phases were filtered through a pad of Na_2SO_4 with Celite® on top. The filtrate was concentrated under reduced pressure to give 792 mg of the crude aryl boronate as a dark solid residue.

Step 2, Suzuki – Miyaura coupling. Half of crude boronate obtained above (~ 0.5 mmol, 1.5 equiv), 6-bromo[1,2,4]triazolo[1,5-*a*]pyridin-2-amine (71.0 mg, 0.333 mmol, 1 equiv), K_2CO_3 (138 mg, 1.0 mmol, 3 equiv), $Pd(PPh_3)_4$ (19.3 mg, 17.7 µmol, 0.05 equiv) followed by degassed DMF – H_2O mixture (2:1, 2.5 mL) were placed into a microwave vial equipped with a stirring bar. The reaction vessel was purged with argon, sealed and kept under microwave irradiation at 100 °C for 3 h. After being cooled to rt, the reaction mixture was concentrated to dryness, then redissolved in 10 mL CH_2Cl_2– MeOH (3:1) and 10 mL H_2O. The organic phase was separated, the aqueous one was extracted with CH_2Cl_2– MeOH (3:1, 3×5 mL); the combined organic extracts were filtered through a pad of Na_2SO_4 with Celite® on top and concentrated under reduced pressure. The solid residue was further purified by column chromatography on SiO_2 (Reveleris®, 0→10% MeOH – CH_2Cl_2 gradient) providing 38.9 mg (20%) of **S7** as a pale beige solid.

^{1}H NMR (600 MHz, DMSO-d_6) δ 8.52 (d, J = 1.7 Hz, 1H), 7.39 (dd, J = 7.4, 2.2 Hz, 2H), 7.37 (dd, J = 9.0, 1.8 Hz, 1H), 7.33 – 7.21 (m, 10H), 6.30 (d, J = 3.0 Hz, 1H), 5.98 (br s, 2H), 5.14 (s, 2H), 4.86 (s, 2H); **^{13}C NMR** (151 MHz, DMSO-d_6) δ 166.3, 162.1, 157.7, 149.2, 137.9, 135.6, 129.7, 128.2, 128.1, 128.0, 127.7, 127.4, 127.3, 125.5, 125.1, 123.6, 120.4, 118.8, 111.6, 107.5, 66.5, 65.3; **LRMS** (ESI) *m/z*: [M+2H-Na]$^+$ calcd for $C_{26}H_{21}N_6NaO_6S$ 547.1, found 547.4; [M-Na]$^-$ calcd 545.1, found 545.2.

General Procedure B – hydrogenolysis of *N*-Cbz and *O*-Bn protected pyrrole-2-carboxylates

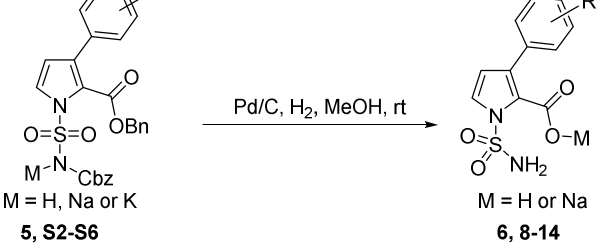

A one-necked round-bottom flask containing 0.025 M methanol solution of the corresponding *O*-Bn- and *N*-Cbz-protected pyrrole (1 equiv) was charged with Pd on carbon (10% w/w, 0.2 equiv), sealed and evacuated/backfilled with dihydrogen gas (3 times). The reaction mixture was hydrogenated at atmospheric pressure (H_2 balloon) overnight under vigorous stirring, then filtered through a pad of Celite® and the filter cake was washed with methanol; the combined filtrates were concentrated to dryness under reduced pressure. Details of further purification are specified below. (NOTE: compounds **4-8**, obtained after purification by prepHPLC and lyophilization contain low levels of formic acid/formate (<<2 w/w% by ^{1}H NMR) and considered as they are in neutral (**4**,**5**,**7**,**8**) or zwitterionic (**6**) form).

3-(4-Fluorophenyl)-1-sulfamoyl-1*H*-pyrrole-2-carboxylic acid (6a)

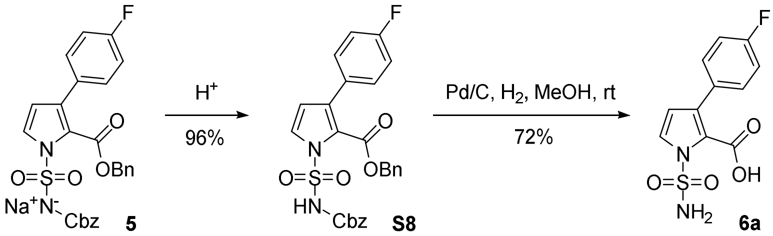

The *N*-Cbz sodium salt **5** (72.7 mg, 0.137 mmol) was dissolved in 10 mL EtOAc, transferred to a separatory funnel and successively washed with 1 M HCl_{aq} (2×5 mL), brine (5 mL) and dried over Na_2SO_4. The solvent was removed *in vacuo* to give **S8** as a yellow oil in 96% yield (67.2 mg).

^{1}H NMR (600 MHz, $CDCl_3$) δ 8.69 (br s, 1H), 7.55 (d, *J* = 3.3 Hz, 1H), 7.38 – 7.34 (m, 3H), 7.34 – 7.28 (m, 3H), 7.27 – 7.22 (m, 4H, overlapped with solvent peak), 7.04 – 7.00 (m, 2H), 6.94 – 6.87 (m, 2H), 6.23 (d, *J* = 3.3 Hz, 1H), 5.18 (s, 2H), 5.12 (s, 2H); **^{19}F NMR** (376 MHz, $CDCl_3$) δ -114.1.

The oil obtained above (67.2 mg) was hydrogenated according to General Procedure B; then the crude residue was further purified by prepHPLC to give the desired compound **6a** in 72% yield (27.0 mg) as a white fluffy solid.

^{1}H NMR (600 MHz, DMSO-d_6) δ 13.17 (br s, 1H), 8.17 (br s, 2H), 7.46 – 7.42 (m, 2H), 7.42 (d, *J* = 3.2 Hz, 1H), 7.24 – 7.18 (m, 2H), 6.37 (d, *J* = 3.2 Hz, 1H); **^{13}C NMR** (151 MHz, DMSO-d_6) δ 162.5, 161.5 (d, *J* = 244.1 Hz), 131.7, 130.75 (d, *J* = 3.4 Hz), 130.69 (d, *J* = 8.2 Hz), 125.2, 120.8, 114.8 (d, *J* = 21.4 Hz), 110.7; **^{19}F NMR** (376 MHz, DMSO-d_6) δ -115.8; **HPLC analysis** t_R 11.3 min, purity >99.6%; **HRMS** (ESI) *m/z*: $[M-H]^-$ calcd for $C_{11}H_8FN_2O_4S$ 283.0194, found 283.0189.

Sodium 3-(4-fluorophenyl)-1-sulfamoyl-1*H*-pyrrole-2-carboxylate (6b)

6b

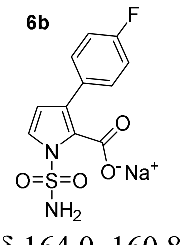

Use of General Procedure B with protected pyrrole **5** (0.111 mmol) gave the desired compound **6b** without further purification in 93% yield (31.5 mg) as an off-white solid. The sample for testing was prepared by dispersing in water and freeze-drying.

^{1}H NMR (600 MHz, DMSO-d_6) δ 8.89 (br s, 2H), 7.56 (dd, *J* = 8.5, 5.6 Hz, 2H), 7.14 – 7.07 (m, 3H), 6.22 (d, *J* = 3.2 Hz, 1H); **^{13}C NMR** (151 MHz, DMSO-d_6) δ 164.0, 160.8 (d, *J* = 242.5 Hz), 132.2 (d, *J* = 3.0 Hz), 130.4 (d, *J* = 8.0 Hz), 128.6 (br), 126.3 (br), 120.2 (br), 114.2 (d, *J* = 21.1 Hz), 108.9; **^{19}F NMR** (376 MHz, DMSO-d_6) δ -117.6; **HPLC analysis** t_R 11.3 min, purity >96.3%; **HRMS** (ESI) *m/z*: $[M-Na]^-$ calcd for

$C_{11}H_8FN_2O_4S$ 283.0194, found 283.0189. The analytical data are consistent with those reported in the literature.[3]

Sodium 3-(4-carbamoylphenyl)-1-sulfamoyl-1*H*-pyrrole-2-carboxylate (8)

8

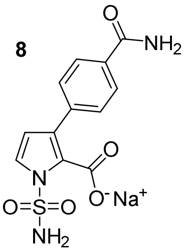

Use of General Procedure B with protected pyrrole **S2** (0.265 mmol) gave the desired compound **8** without further purification in 96% yield as a pale beige solid (84.1 mg). The sample for testing was prepared by dissolution in minimal amount of water, filtration through 0.22 µm nylon syringe filter followed by lyophilization.

^{1}H NMR (600 MHz, DMSO-d_6) δ 8.82 (br s, 2H), 7.93 (br s, 1H), 7.79 (d, *J* = 8.0 Hz, 2H), 7.60 (d, *J* = 8.1 Hz, 2H), 7.25 (br s, 1H), 7.11 (d, *J* = 3.2 Hz, 1H), 6.29 (d, *J* = 3.2 Hz, 1H); **^{13}C NMR** (151 MHz, DMSO-d_6) δ 167.9, 164.2, 138.8, 131.6, 129.5 (br), 128.0, 126.9, 126.0 (br), 120.1 (br), 108.7; **HPLC analysis** t_R 9.0 min, purity >95.6%; **HRMS** (ESI) *m/z*: [M-Na]$^-$ calcd for $C_{12}H_{10}N_3O_5S$ 308.0347, found 308.0343.

Sodium 3-(6-aminopyridin-3-yl)-1-sulfamoyl-1*H*-pyrrole-2-carboxylate (9)

9

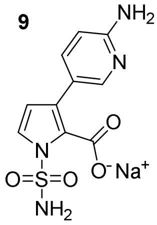

Use of General Procedure B with protected pyrrole **S3** (0.125 mmol) gave the desired compound **9** without further purification in 45% yield as a brown solid (16.9 mg). The sample for testing was prepared by dissolution in minimal amount of water, filtration through 0.22 µm nylon syringe filter followed by lyophilization.

^{1}H NMR (600 MHz, DMSO-d_6) δ 8.98 (br s, 2H), 8.04 (d, *J* = 2.3 Hz, 1H), 7.60 (dd, *J* = 8.5, 2.4 Hz, 1H), 7.01 (d, *J* = 3.2 Hz, 1H), 6.36 (d, *J* = 8.5 Hz, 1H), 6.10 (d, *J* = 3.2 Hz, 1H), 5.71 (br s, 2H); **^{13}C NMR** (151 MHz, DMSO-d_6) δ 164.5, 157.8, 147.0, 137.6, 129.2, 124.4, 120.4, 119.4, 108.0, 106.6; **HPLC analysis** t_R 6.7 min, purity >94.8%; **HRMS** (ESI) *m/z*: [M-Na]$^-$ calcd for $C_{10}H_9N_4O_4S$ 281.0350, found 281.0347. Analytical data is consistent with that reported in the literature.[3]

3-(1*H*-Pyrazol-4-yl)-1-sulfamoyl-1*H*-pyrrole-2-carboxylic acid (10)

10

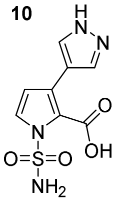

Use of General Procedure B with protected pyrrole **S4** (78.8 µmol) followed by purification by prepHPLC (standard conditions) and lyophilization gave the desired compound **10** as a white fluffy solid in 75% yield (15.1 mg).

^{1}H NMR (600 MHz, DMSO-d_6) δ 13.06 (br s, 2H), 8.04 (br s, 2H), 7.90 (s, 2H), 7.39 (d, *J* = 3.2 Hz, 1H), 6.46 (d, *J* = 3.2 Hz, 1H); **^{13}C NMR** (151 MHz, DMSO-d_6) δ 162.6, 133.2 (br), 126.1, 125.5, 119.3, 113.4, 109.9; **HPLC analysis** t_R 7.8 min, purity >97.0%; **HRMS** (ESI) *m/z*: [M+H]$^+$ calcd for $C_8H_9N_4O_4S$ 257.0339, found 257.0340.

3-(2-Aminopyrimidin-5-yl)-1-sulfamoyl-1*H*-pyrrole-2-carboxylic acid (11) and 3-(2-iminohexahydropyrimidin-5-yl)-1-sulfamoyl-1*H*-pyrrole-2-carboxylic acid (12)

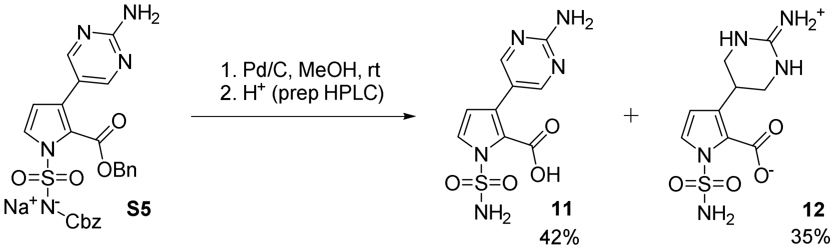

Use of General procedure B for 60 h at room temperature with protected pyrrole **S5** (68.6 µmol) followed by purification by prepHPLC (0→70% mobile phase B gradient, 30 min) and lyophilization afforded the aminopyrimidine **11** as a white fluffy solid in 42% yield (8.2 mg) and the guanidine **12** as a white fluffy solid in 35% yield (7.0 mg).

Analytical data for **11**: **^{1}H NMR** (600 MHz, DMSO-d_6) δ 13.17 (br s, 1H), 8.28 (s, 2H), 8.16 (br s, 2H), 7.46 (d, *J* = 3.2 Hz, 1H), 6.72 (br s, 2H), 6.37 (d, *J* = 3.2 Hz, 1H); **^{13}C NMR** (151 MHz, DMSO-d_6) δ 162.5, 162.1, 157.6, 128.8 (br), 126.3 (br), 120.3 (br), 116.8, 110.2. **HPLC analysis**, t_R 6.4 min and 6.5 min (insufficient protonation by mobile phase), purity >99.8%; **HRMS** (ESI) *m/z*: $[M+H]^+$ calcd for $C_9H_{10}N_5O_4S$ 284.0448, found 284.0447.

Analytical data for **12**: **^{1}H NMR** (600 MHz, DMSO-d_6) δ 8.93 (br s, 2H), 8.53 (br s, 2H), 7.24 (br s, 2H), 7.11 (d, *J* = 3.2 Hz, 1H), 6.06 (d, *J* = 3.2 Hz, 1H), 3.83 (tt, *J* = 10.1, 4.6 Hz, 1H), 3.40 (dt, *J* = 11.6, 4.3 Hz, 2H, overlapped with residual water peak), 3.21 (t, *J* = 11.2 Hz, 2H, overlapped with residual water peak); **^{13}C NMR** (151 MHz, DMSO-d_6) δ 164.5, 153.8, 128.6 (br), 128.0 (br), 121.6 (br), 106.0, 42.9, 28.6; **HPLC analysis** t_R 6.1 min, purity >98.9%; **HRMS** (ESI) *m/z*: $[M+H]^+$ calcd for $C_9H_{14}N_5O_4S$ 288.0761, found 288.0761.

3-(4-Morpholinophenyl)-1-sulfamoyl-1*H*-pyrrole-2-carboxylic acid (13)

13

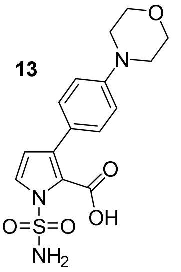

Use of General Procedure B with protected pyrrole **S6** (0.132 mmol) followed by purification by prepHPLC (standard conditions) and lyophilization gave the desired compound **13** as an off-white fluffy solid in 44% yield (20.2 mg).

^{1}H NMR (600 MHz, DMSO-d_6) δ 13.01 (br s, 1H), 8.13 (br s, 2H), 7.36 (d, *J* = 3.2 Hz, 1H), 7.32 – 7.27 (m, 2H), 6.96 – 6.91 (m, 2H), 6.31 (d, *J* = 3.2 Hz, 1H), 3.76 – 3.73 (m, 4H), 3.15 – 3.11 (m, 4H); **^{13}C NMR** (151 MHz, DMSO-d_6) δ 163.0, 150.1, 132.5 (br), 129.3, 124.8 (br), 124.8, 120.5 (br), 114.4, 110.5, 66.1, 48.2; **HPLC analysis** t_R 9.0 min, purity >96.3%; **HRMS** (ESI) *m/z*: $[M+H]^+$ calcd for $C_{15}H_{18}N_3O_5S$ 352.0962, found 352.0962.

3-(2-Amino-[1,2,4]triazolo[1,5-a]pyridin-6-yl)-1-sulfamoyl-1*H*-pyrrole-2-carboxylic acid (14)

14

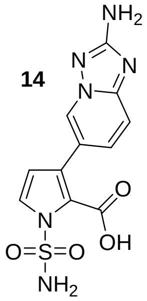

Use of General Procedure B with protected pyrrole **S7** (63.3 µmol) followed by purification by prepHPLC (standard conditions) and lyophilization gave the desired compound **13** as a white solid in 10% yield (2.0 mg).

^{1}H NMR (600 MHz, DMSO-*d*$_6$) δ 8.65 – 8.58 (m, 1H), 8.26 (br s, 2H), 7.47 (dd, *J* = 9.1, 1.8 Hz, 1H), 7.45 (d, *J* = 3.1 Hz, 1H), 7.34 (dd, *J* = 9.1, 0.6 Hz, 1H), 6.46 (d, *J* = 3.1 Hz, 1H), 6.00 (br s, 2H); **^{13}C NMR** (151 MHz, DMSO-*d*$_6$) δ 166.4, 162.2, 149.5, 130.7, 128.6 (br), 126.4, 125.4 (br), 121.6 (br), 118.5, 111.2, 110.7; **HPLC analysis** t_R 7.1 min, purity >99.1%; **HRMS** (ESI) *m/z*: $[M+H]^+$ calcd for $C_{11}H_{11}N_6O_4S$ 323.0557, found 323.0557.

2 Biochemical Assays

The inhibitory activity of NSPCs **6**, **8-14** against representative B1 subfamily MBLs [obtained according to previously reported production and purification procedures: VIM-1,[5] VIM-2, NDM-1 and IMP-1[6]] was determined using a fluorogenic assay monitoring the enzymatic breakdown of the cephalosporin probe FC5.[6] The FC5/meropenem assays were conducted at room temperature in clear-bottomed Greiner 384 black well microplates (FC5) or Greiner 96 well UV star microplates (meropenem), using a ClarioStar or PHERAstar FS microplate reader (BMG LabTech). Representative β-lactamases were tested at the following concentrations: VIM-1, 100 pM; NDM-1, 20 pM; IMP-1, 20 pM; and VIM-2, 500 pM. The concentration of FC5 employed was 5 µM for all enzymes. IMP-1, VIM-1, VIM-2 and NDM-1 inhibition assays were screened in "MBL buffer" (50 mM HEPES, pH 7.2, 1 µM $ZnSO_4$, 1 µg mL−1 BSA, 0.01% v/v Triton X-100). The initial rates of reaction (measured after 10 min pre-incubation of the NSPC with the enzyme) were assessed by monitoring the fluorescence intensity at λ_{ex} = 380 nm and λ_{em} = 460 nm. Following the determination of initial rates of reaction, the data were fitted using a four-parameter function: log (inhibitor) vs. response, variable slope in GraphPad Prism 6 to obtain IC50 values.[6, 7]

3 Crystallography

Single VIM-1 crystals were grown as reported previously.[8] The VIM-1:Zn_2:**6** complex was obtained by mixing the crystal containing droplet (4.5 mL) with the same volume of the sulfamoyl compound (4.5 mL, 12.5 mM in VIM-1 crystallization buffer (2.4 mM $NH_4H_2PO_4$, 0.1 M Tris pH 8.5)). The crystals were harvested and cryo-cooled in liquid N_2. Data for the single crystal were collected at 100 K using synchrotron radiation at the Diamond Light Source beamline I24 and processed using the Xia2 pipelines (Table S1).[9] Structures were solved by isomorphous molecular replacement using reported structural data file of VIM-1 (PDB: 5N5G)[8] as a search model. The structure was iteratively fitted and refined using PHENIX[10] and Coot.[11] Processing and refinement statistics are shown in Table S1.

Table S1: Data collection and Refinement statistics.

**Dataset**	**IPNS:Fe:ACV[a]** (PDB ID: 6ZAE)
**Data Collection (T in K)**	MFX (100)
Beamline (Wavelength, Å)	DLS I24 (0.9686)
Detector	Dectris Pilatus3 6M
Data Processing	Xia 2
Space group	$P\ 1\ 2_1\ 1$
Cell dimensions	
a,b,c (Å)	39.53, 67.43, 40.27
α, β, γ (°)	90, 93.51, 90
No. of molecules/ASU	1
No. reflections	64128 (3074)*
Resolution (Å)	40.19-1.21 (1.23-1.21)*
R_{merge}	0.049 (0.628)*
I/σI	16.30 (2.20)*
CC-half	0.999 (0.721)*
Completeness (%)	99.8 (95.9)*
Multiplicity	6.30 (5.60)*
Wilson B value (Å^2)	12.68
**Refinement**	PHENIX
R_{work}/R_{free+}	0.1697/0.2951
No. atoms	2227
- Enzyme	1924
- ligand	21
- Water	282
Average B-factors	20.47
- Enzyme	19.14
- ligand	22.53
- Water	29.43
R.m.s deviations	
- Bond lengths (Å)	0.009
- Bond angles (°)	1.10

*Highest resolution shell in parentheses. DLS = Diamond Light Source.

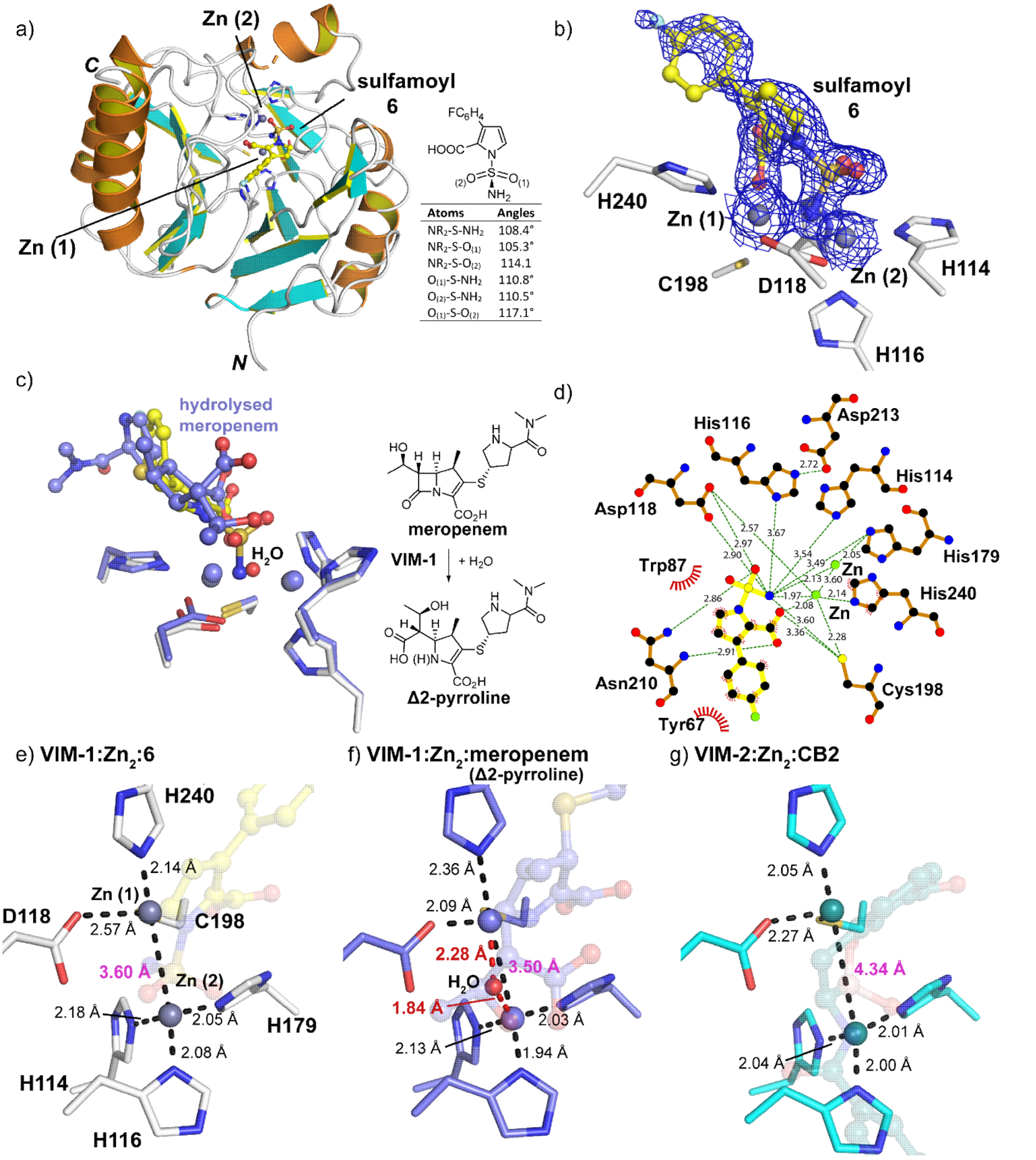

Figure S1. **Comparison of the binding mode of 6 with those of CB2 and meropenem.** a) Ribbons view of VIM-1:Zn$_2$:**6** (PDB: 7AYJ). The *N*-sulfamoylpyrrole-2-carboxylate **6** NSPC **6**, yellow) and Zn-interacting residues (H114, H116, D118, H179, C198, H240) are shown as sticks. The secondary structure elements α-helices (orange) and β-strands (cyan) are shown as cartoons. b) Composite 2mF$_o$-DF$_c$ omit map of VIM-1:Zn$_2$:**6** (PDB: 7AYJ, 1.21 Å resolution, 1.0 σ contour level) showing the NSPC binding to VIM-1. Note, C198 is partially oxidized so that the structure was deposited with 75% Cys198 and 25% Csd198 (3-sulfonoalanine, data not shown). c) Superimposition of VIM-1:Zn$_2$:**6** (PDB: 7AYJ) and VIM-1:Zn$_2$:meropenem (hydrolyzed meropenem, Δ^2-pyrroline form, PDB: 5N5I)[8] structures. Note, while the hydrolyzed meropenem structure has a Zn-coordinated water present, the sulfamoyl NH$_2$ group of **6** replaces this water. d) Key interactions at the active site. Comparison of the Zn(1)-Zn(2) distances in e) VIM-1:Zn$_2$:**6** (PDB: 7AYJ, 3.60 Å, this study), f) VIM-1:Zn$_2$:meropenem (Δ^2-pyrroline form, PDB: 5N5I, 3.50 Å),[8] and the cyclic boronate VIM-2:Zn$_2$:CB2 complex (PDB:

5FQC, 4.34 Å)[12] reveal the impact of the cyclic boronate ligand on the Zn(1)-Zn(2) distance, whereas the impact of the sulfamoyl ligand on the Zn(1)-Zn(2) distance is negligible. Note, VIM-1:Zn_2 (PDB: 5N5G, not shown)[8] and VIM-2:Zn_2 (PDB: 4NQ2, not shown)[13] structures without ligands reveal Zn(1)-Zn(2) distances of 3.62 Å and 3.47 Å, respectively in accord with former reports.[14]

4 References

1. Steeds, H. G.; Knowles, J. P.; Yu, W. L.; Richardson, J.; Cooper, K. G.; Booker-Milburn, K. I., Rapid Access to Azabicyclo[3.3.1]nonanes by a Tandem Diverted Tsuji–Trost Process. *Chem. Eur. J.* **2020,** *26* (63), 14330-14334.
2. Ohta, T.; Fukuda, T.; Ishibashi, F.; Iwao, M., Design and Synthesis of Lamellarin D Analogues Targeting Topoisomerase I. *J. Org. Chem.* **2009,** *74* (21), 8143-8153.
3. Wilkinson, A.; Cooper, I.; Orr, D.; Finlayson, J.; Bunt, A.; Appelqvist, P.; Wallberg, H.; Wångsell, F., Antibacterial compounds. *WO 2019220125* **2019**.
4. Lu, X.; Zhang, H.; Tonge, P. J.; Tan, D. S., Mechanism-based inhibitors of MenE, an acyl-CoA synthetase involved in bacterial menaquinone biosynthesis. *Bioorg. Med. Chem. Lett.* **2008,** *18* (22), 5963-5966.
5. Makena, A.; Düzgün, A. Ö.; Brem, J.; McDonough, M. A.; Rydzik, A. M.; Abboud, M. I.; Saral, A.; Çiçek, A. Ç.; Sandalli, C.; Schofield, C. J., Comparison of Verona Integron-Borne Metallo-β-Lactamase (VIM) Variants Reveals Differences in Stability and Inhibition Profiles. *Antimicrob. Agents Chemother.* **2015,** *60* (3), 1377-1384.
6. van Berkel, S. S.; Brem, J.; Rydzik, A. M.; Salimraj, R.; Cain, R.; Verma, A.; Owens, R. J.; Fishwick, C. W. G.; Spencer, J.; Schofield, C. J., Assay Platform for Clinically Relevant Metallo-beta-lactamases. *J. Med. Chem.* **2013,** *56* (17), 6945-6953.
7. Krajnc, A.; Brem, J.; Hinchliffe, P.; Calvopiña, K.; Panduwawala, T. D.; Lang, P. A.; Kamps, J. J. A. G.; Tyrrell, J. M.; Widlake, E.; Saward, B. G.; Walsh, T. R.; Spencer, J.; Schofield, C. J., Bicyclic Boronate VNRX-5133 Inhibits Metallo- and Serine-β-Lactamases. *J. Med. Chem.* **2019,** *62* (18), 8544-8556.
8. Salimraj, R.; Hinchliffe, P.; Kosmopoulou, M.; Tyrrell, J. M.; Brem, J.; van Berkel, S. S.; Verma, A.; Owens, R. J.; McDonough, M. A.; Walsh, T. R.; Schofield, C. J.; Spencer, J., Crystal structures of VIM-1 complexes explain active site heterogeneity in VIM-class metallo-beta-lactamases. *FEBS J.* **2019,** *286* (1), 169-183.
9. Winter, G.; Lobley, C. M. C.; Prince, S. M., Decision making in xia2. *Acta Crystallograph. Section D* **2013,** *69* (7), 1260-1273.
10. Adams, P. D.; Grosse-Kunstleve, R. W.; Hung, L. W.; Ioerger, T. R.; McCoy, A. J.; Moriarty, N. W.; Read, R. J.; Sacchettini, J. C.; Sauter, N. K.; Terwilliger, T. C., PHENIX: building new software for automated crystallographic structure determination. *Acta Crystallogr. D Biol Crystallogr.* **2002,** *58* (Pt 11), 1948-54.
11. Emsley, P.; Lohkamp, B.; Scott, W. G.; Cowtan, K., Features and development of Coot. *Acta Crystallogr. D Biol. Crystallogr.* **2010,** *66* (Pt 4), 486-501.
12. Brem, J.; Cain, R.; Cahill, S.; McDonough, M. A.; Clifton, I. J.; Jimenez-Castellanos, J. C.; Avison, M. B.; Spencer, J.; Fishwick, C. W.; Schofield, C. J., Structural basis of metallo-beta-lactamase, serine-beta-lactamase and penicillin-binding protein inhibition by cyclic boronates. *Nat. Commun.* **2016,** *7,* 12406.

13. Aitha, M.; Marts, A. R.; Bergstrom, A.; Møller, A. J.; Moritz, L.; Turner, L.; Nix, J. C.; Bonomo, R. A.; Page, R. C.; Tierney, D. L.; Crowder, M. W., Biochemical, Mechanistic, and Spectroscopic Characterization of Metallo-β-lactamase VIM-2. *Biochemistry* **2014,** *53* (46), 7321-7331.
14. Meini, M.-R.; Llarrull, L. I.; Vila, A. J., Overcoming differences: The catalytic mechanism of metallo-β-lactamases. *FEBS Lett.* **2015,** *589* (22), 3419-3432.

5 NMR spectra

^{1}H NMR of **2** (400 MHz, $CDCl_3$)

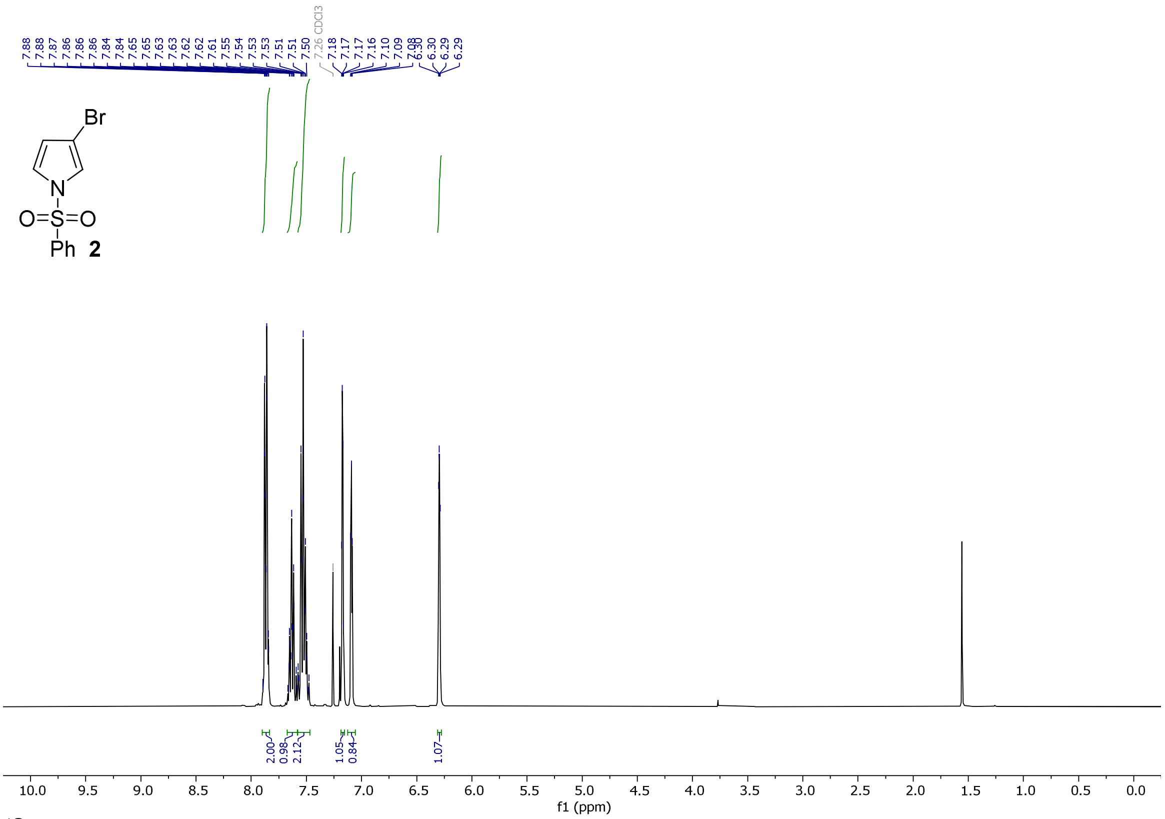

^{13}C NMR of **2** (101 MHz, $CDCl_3$)

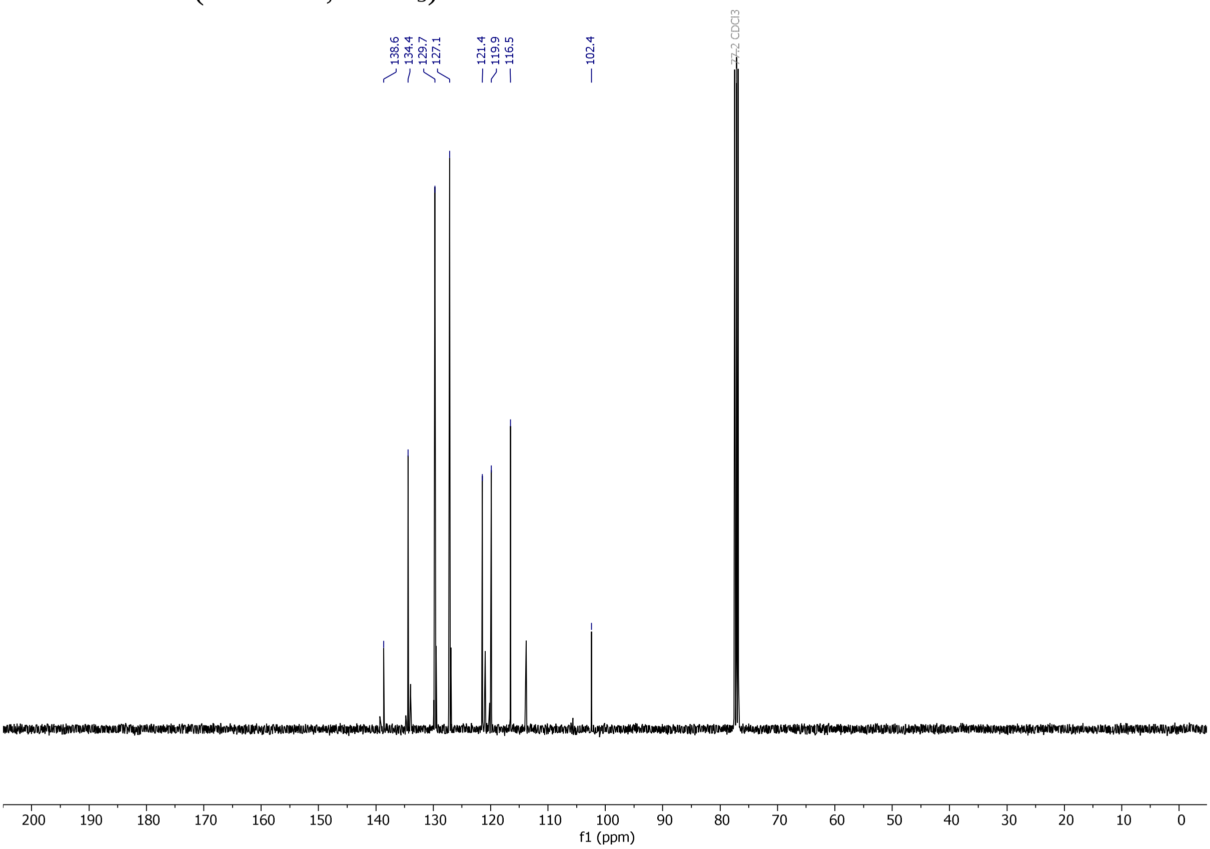

^{1}H NMR of **S1** (400 MHz, $CDCl_3$)

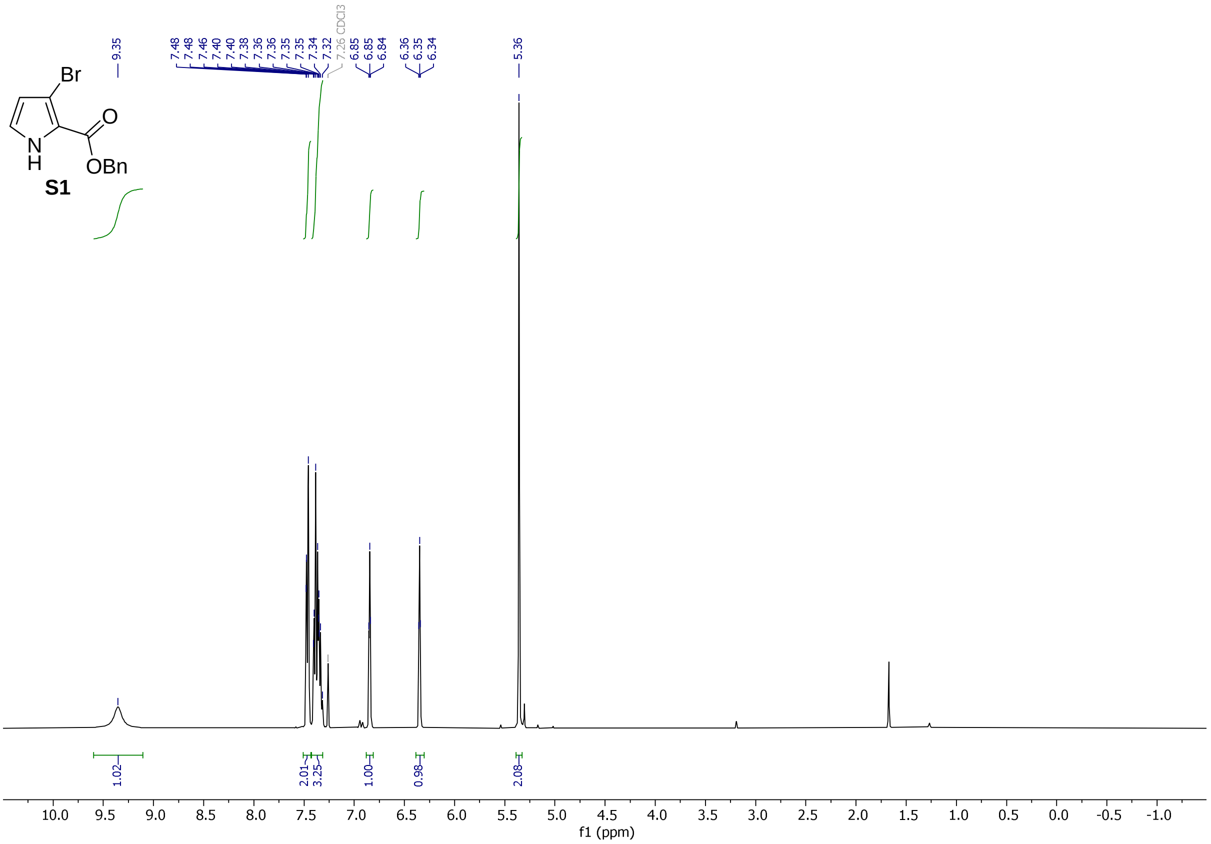

^{13}C NMR of **S1** (101 MHz, $CDCl_3$)

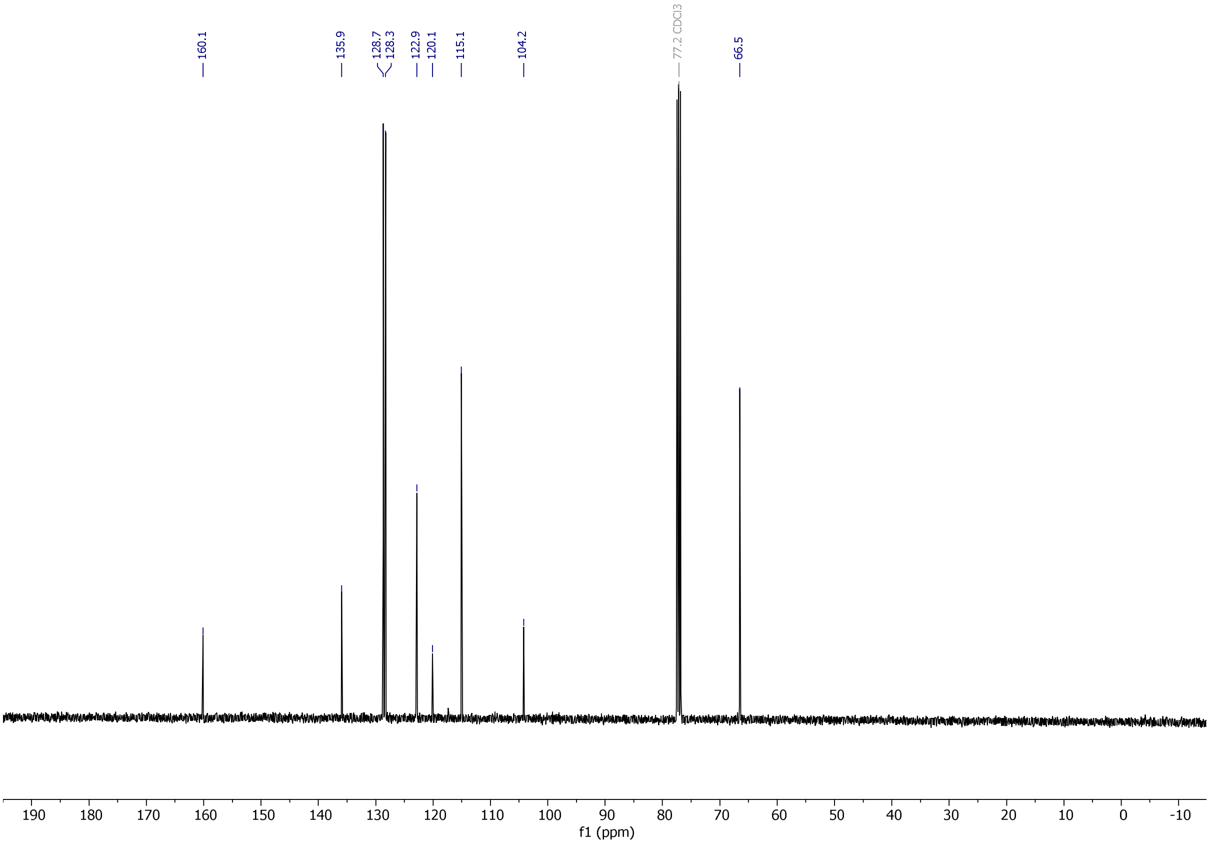

^{1}H NMR of **7** (400 MHz, DMSO-*d*$_6$)

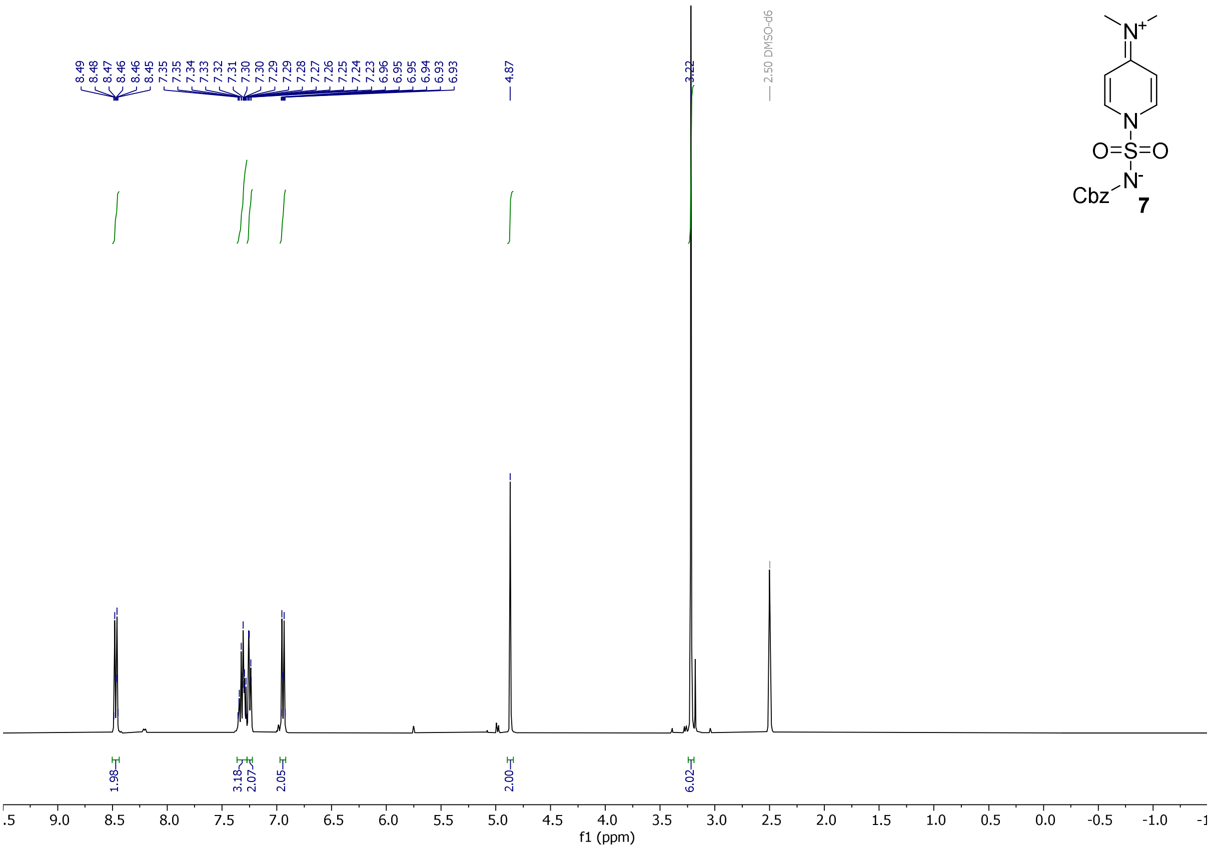

^{13}C NMR of **7** (101 MHz, DMSO-*d*$_6$)

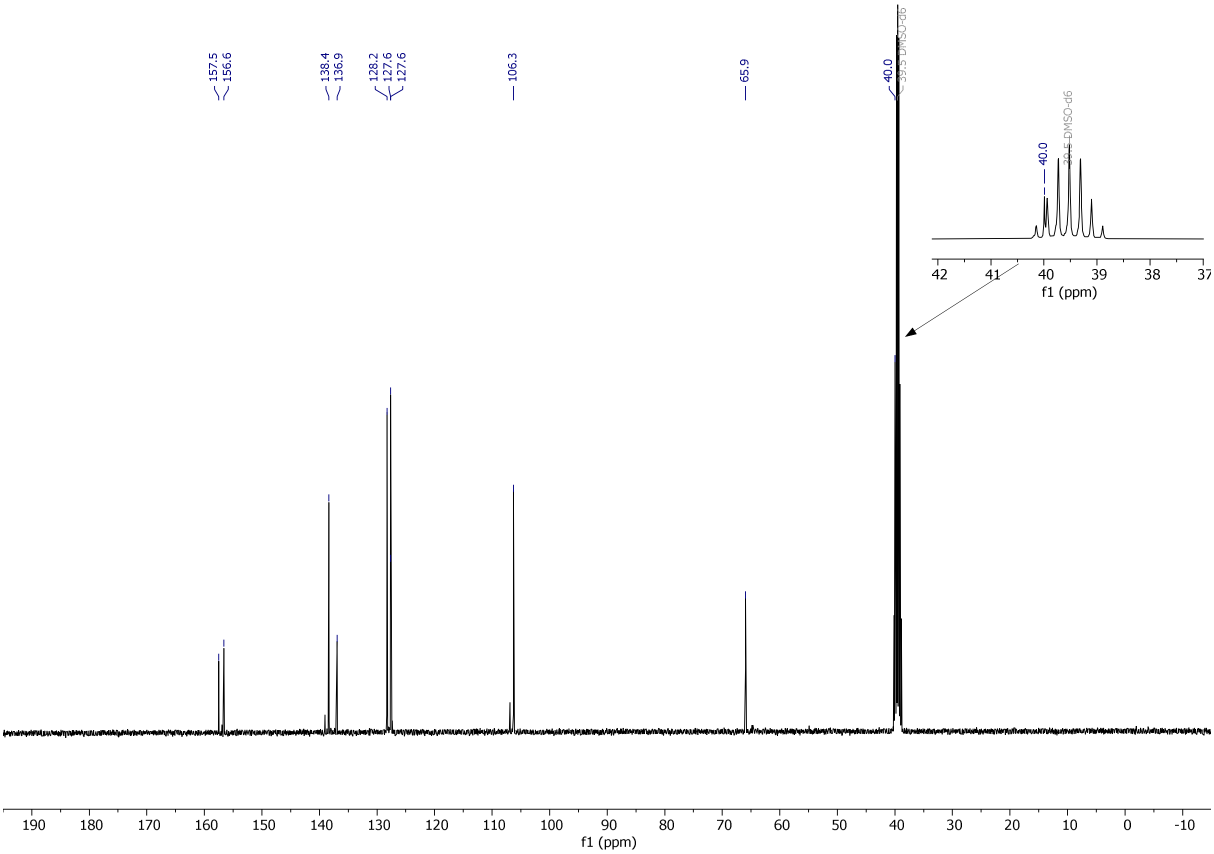

^{1}H NMR of **4** (400 MHz, $CDCl_3$)

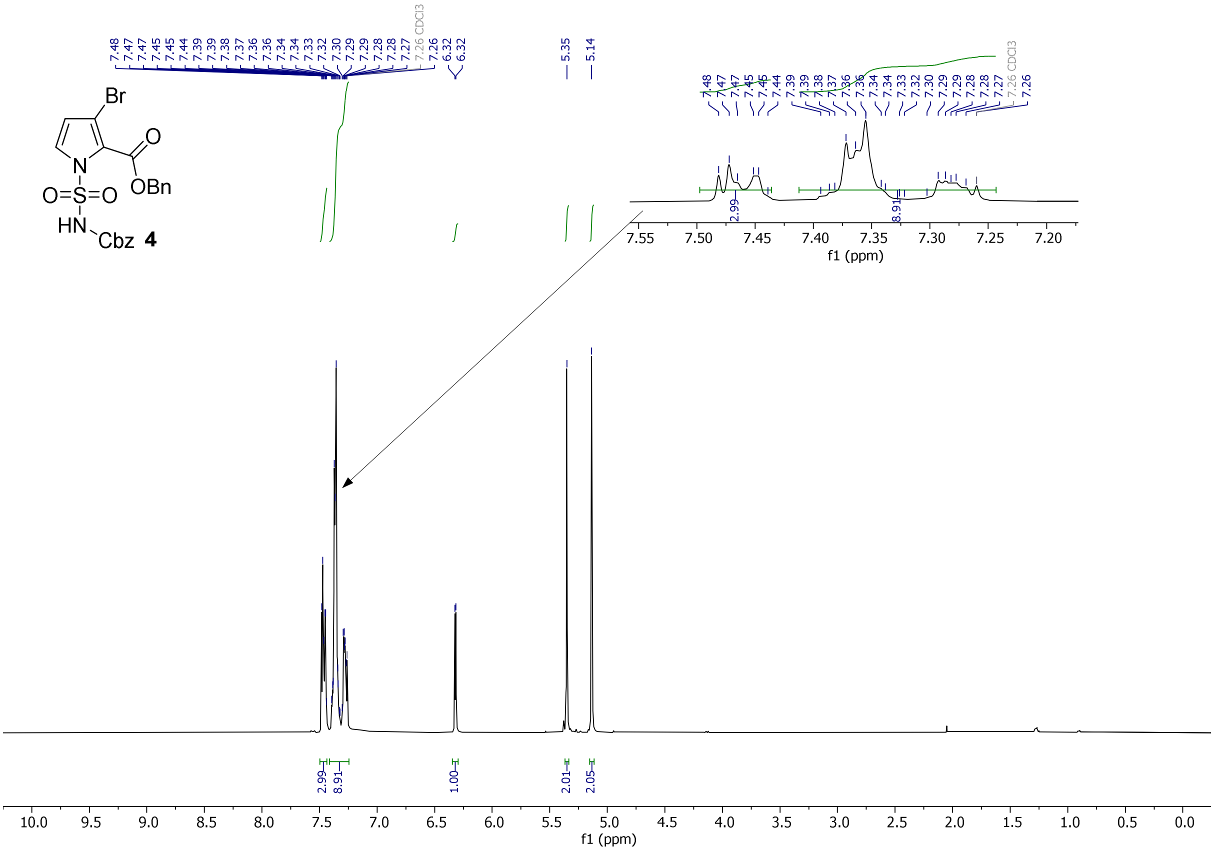

^{13}C NMR of **4** (101 MHz, $CDCl_3$)

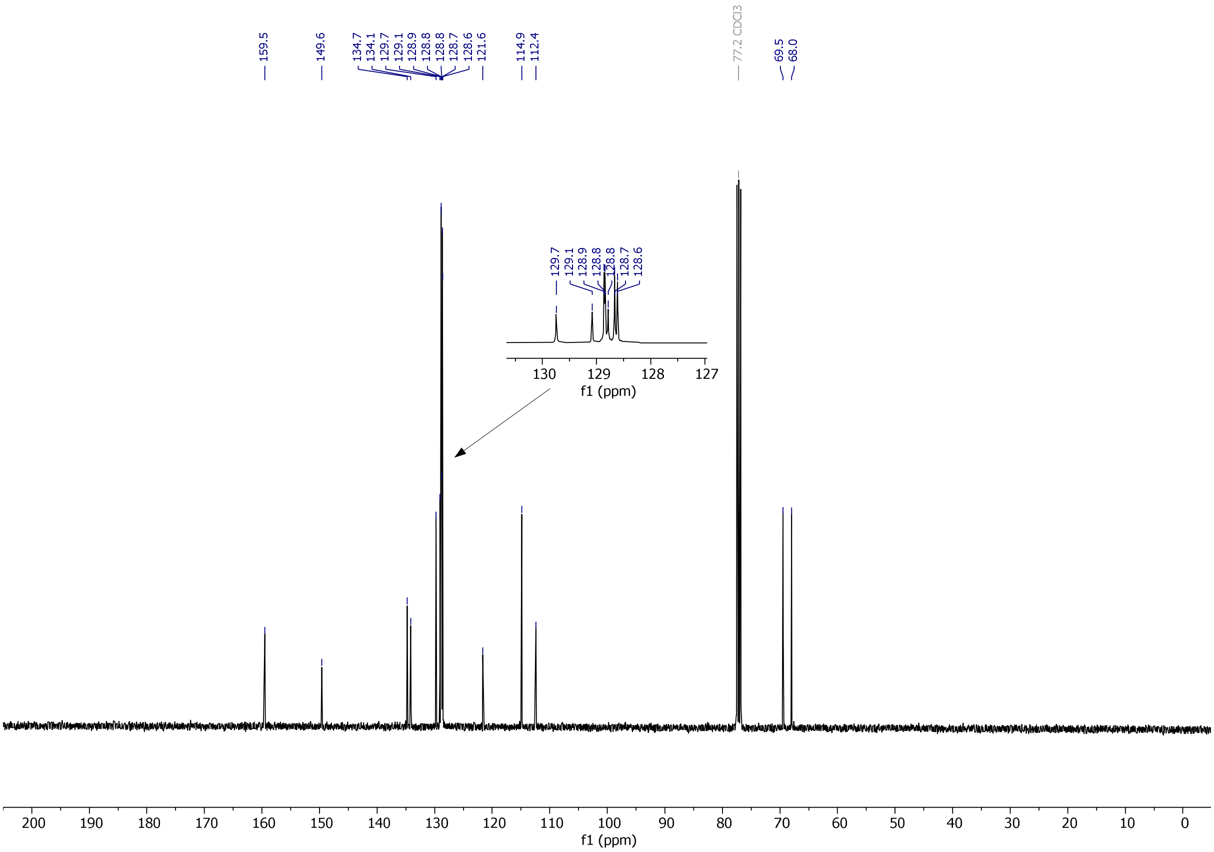

^{1}H NMR of **5** (600 MHz, $CDCl_3$)

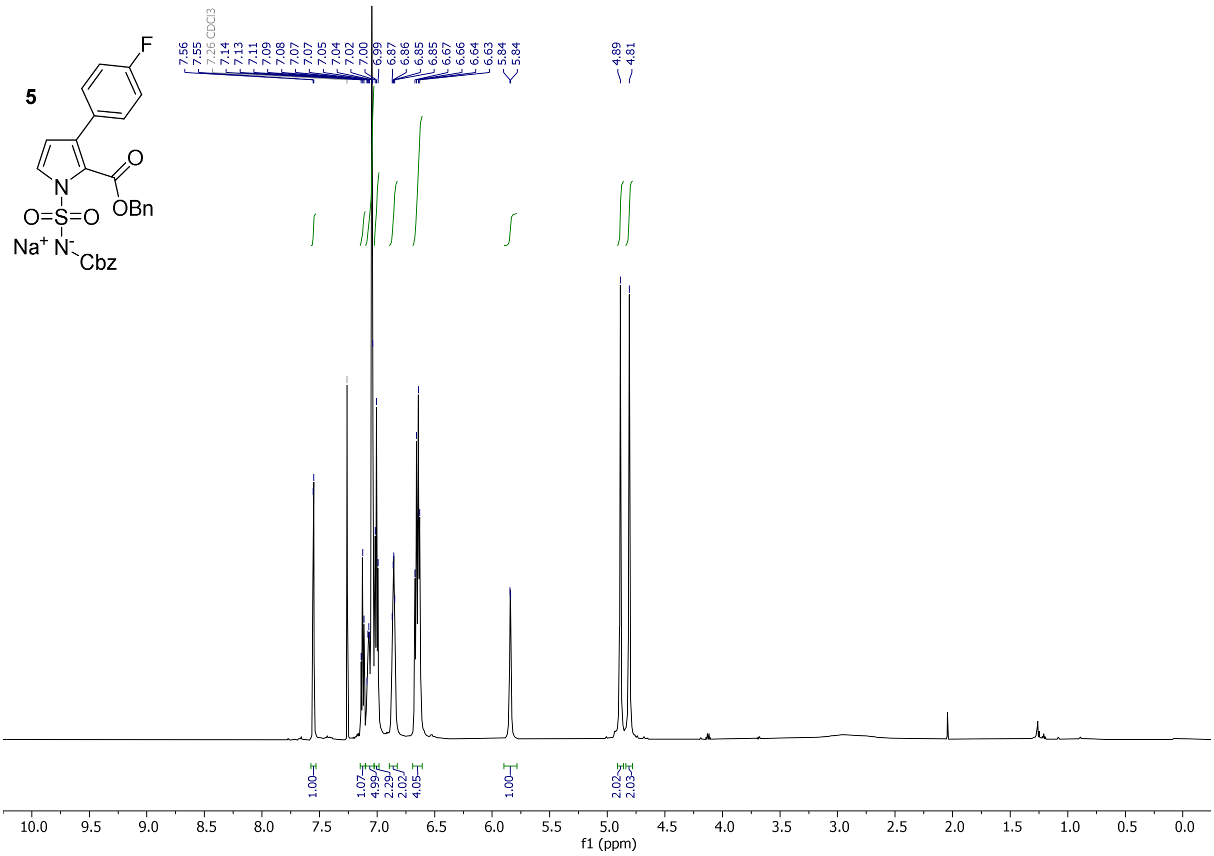

^{13}C NMR of **5** (151 MHz, $CDCl_3$)

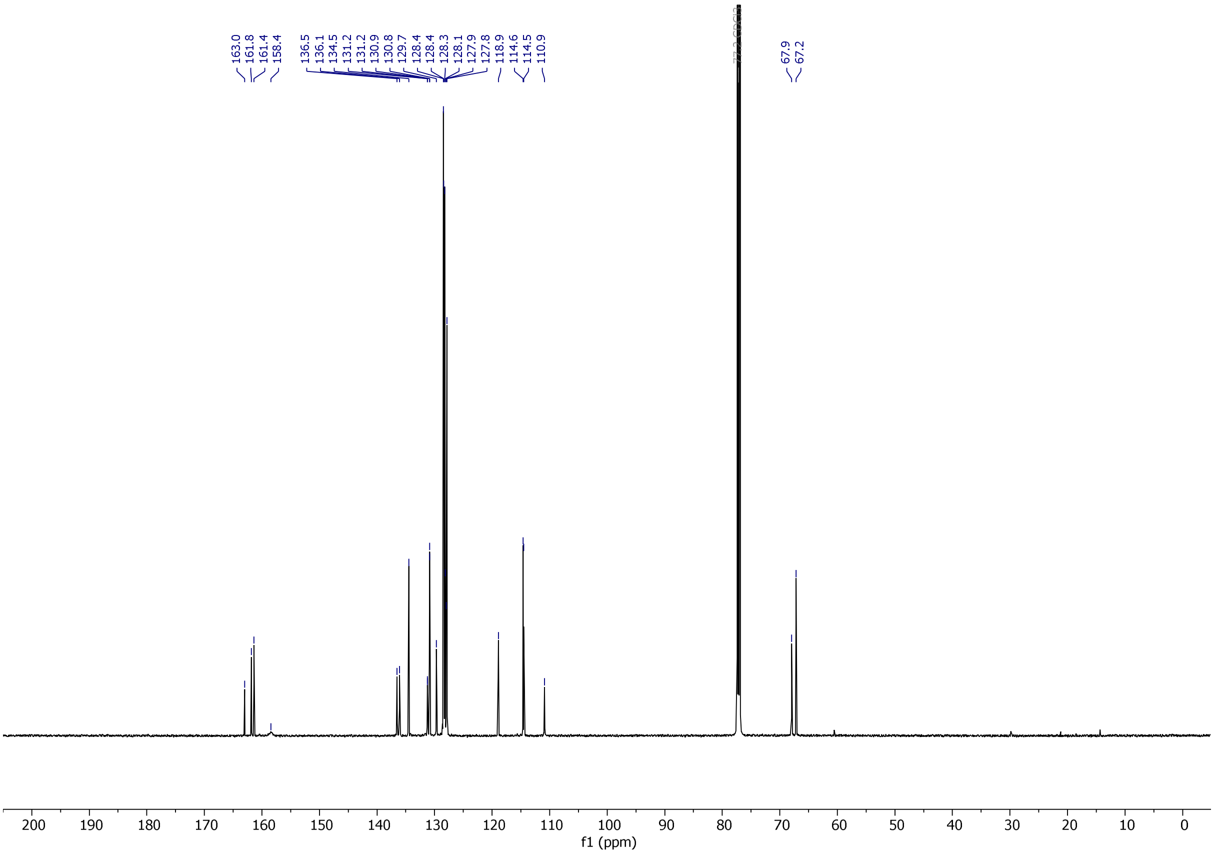

^{19}F NMR of **5** (376 MHz, $CDCl_3$)

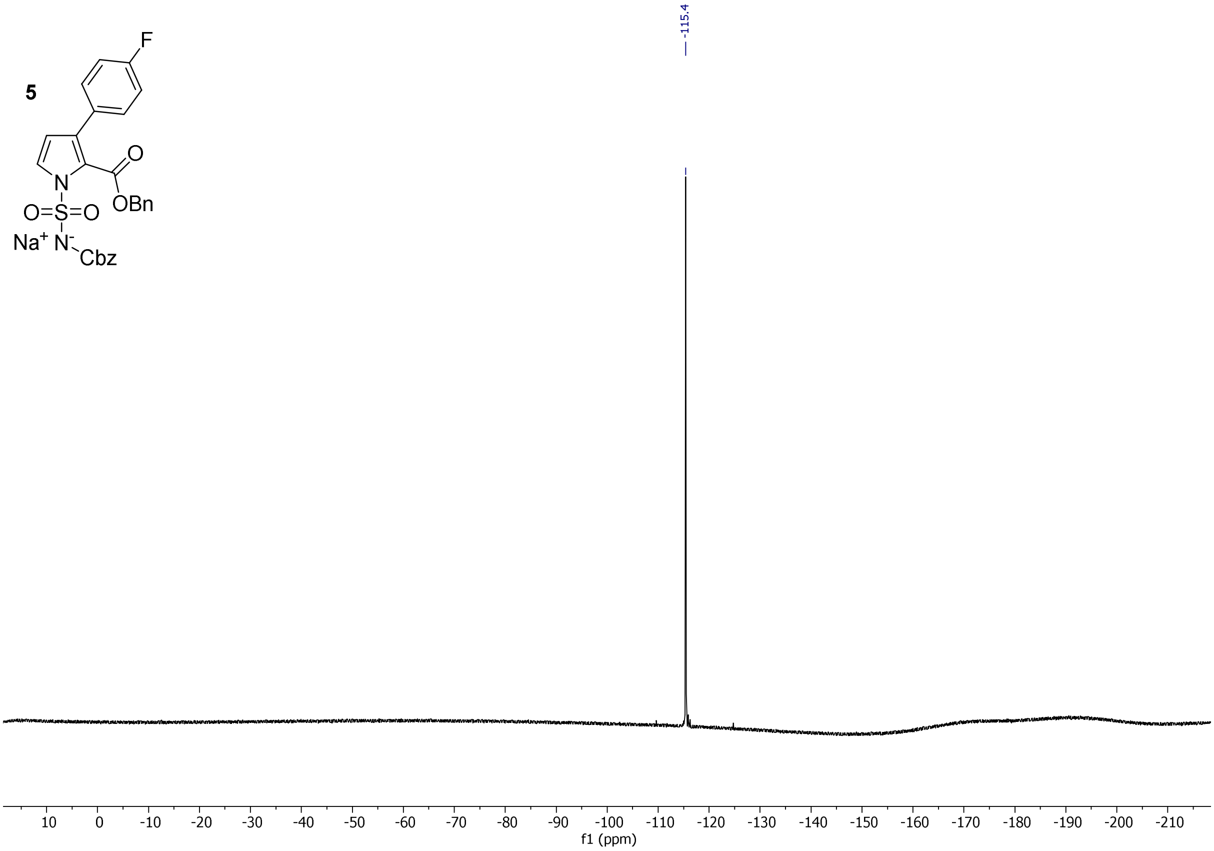

^{1}H NMR of **S2** (600 MHz, DMSO-d_6)

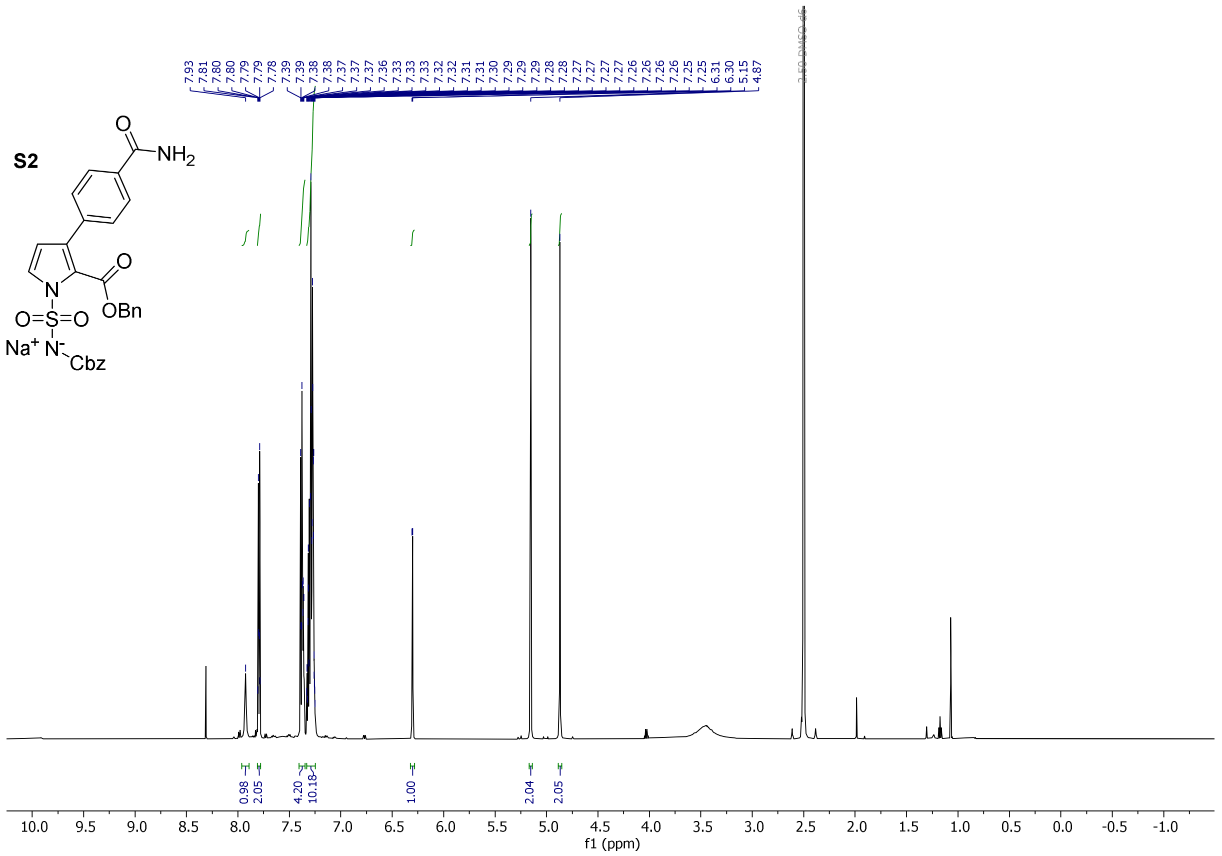

^{13}C NMR of **S2** (151 MHz, DMSO-d_6)

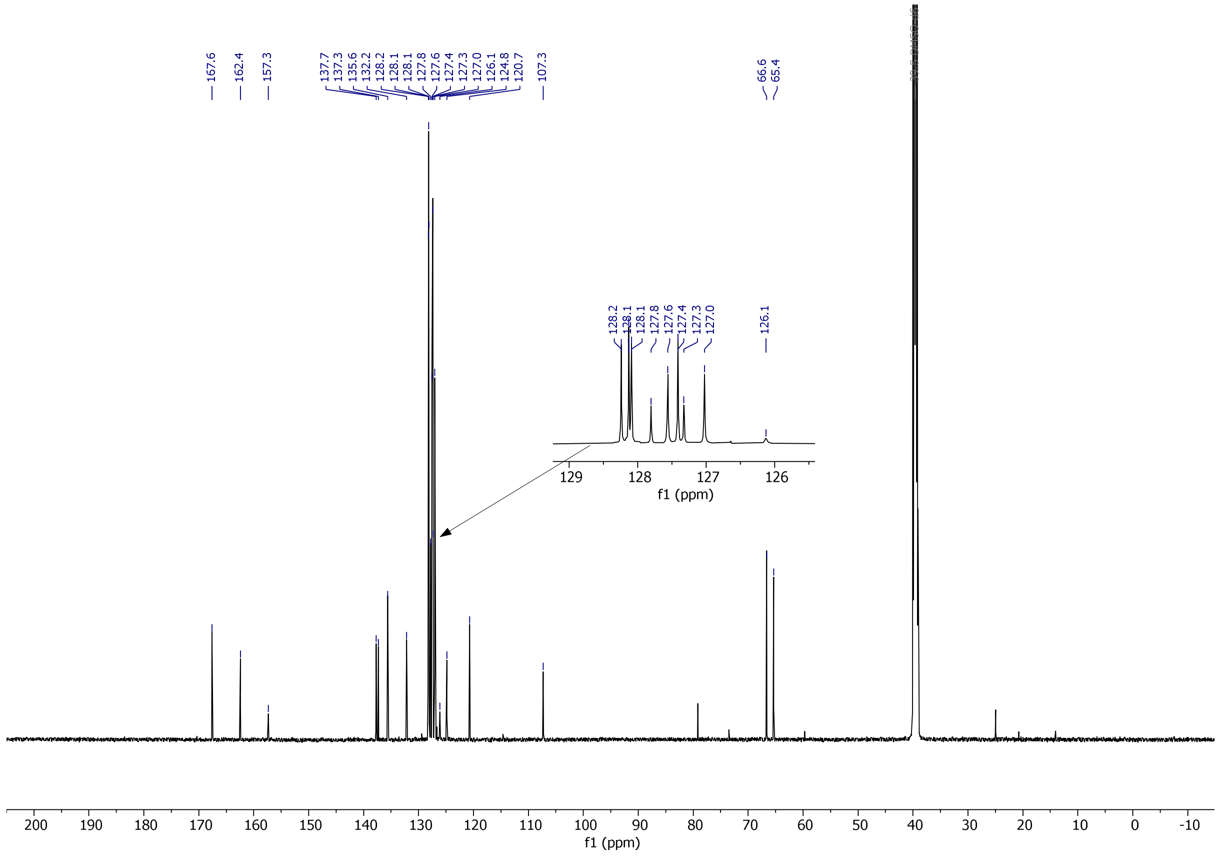

^{1}H NMR of **S3** (600 MHz, DMSO-d_6)

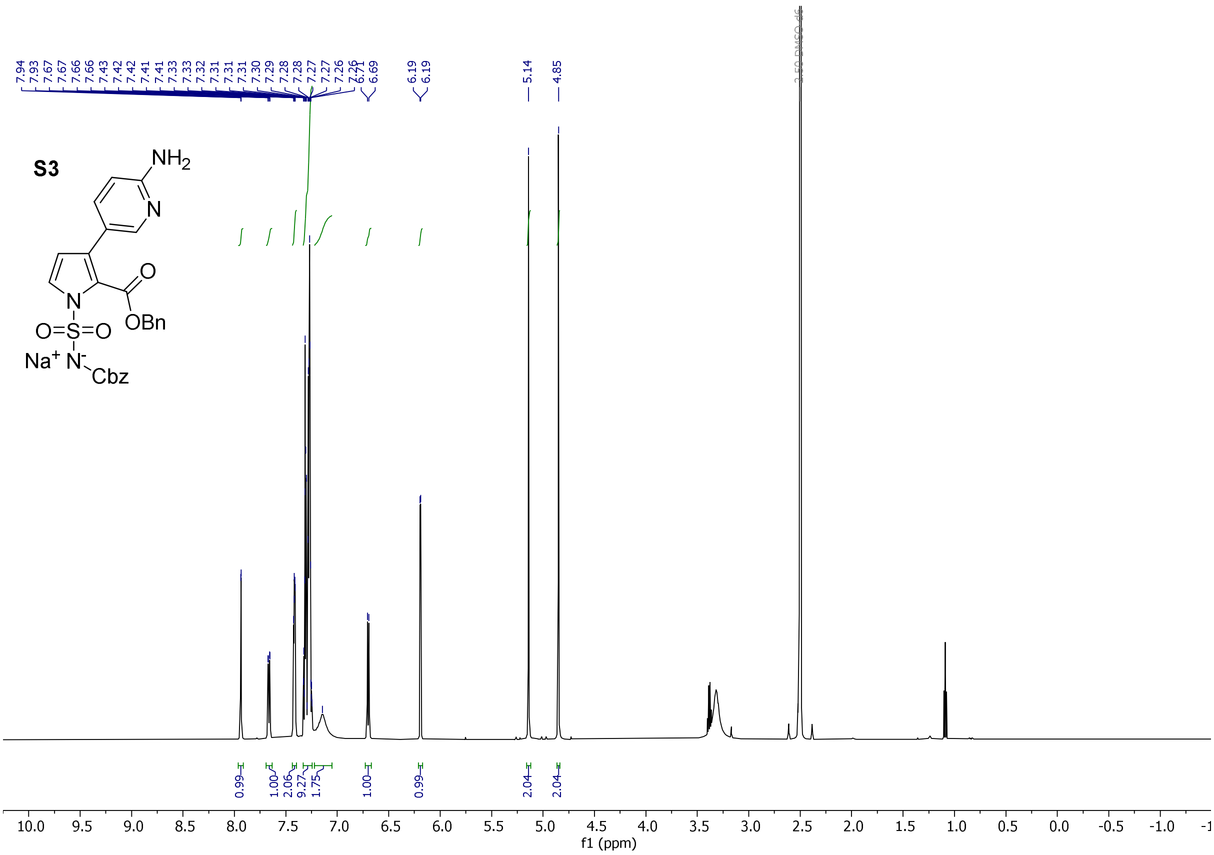

^{13}C NMR of **S3** (151 MHz, DMSO-d_6)

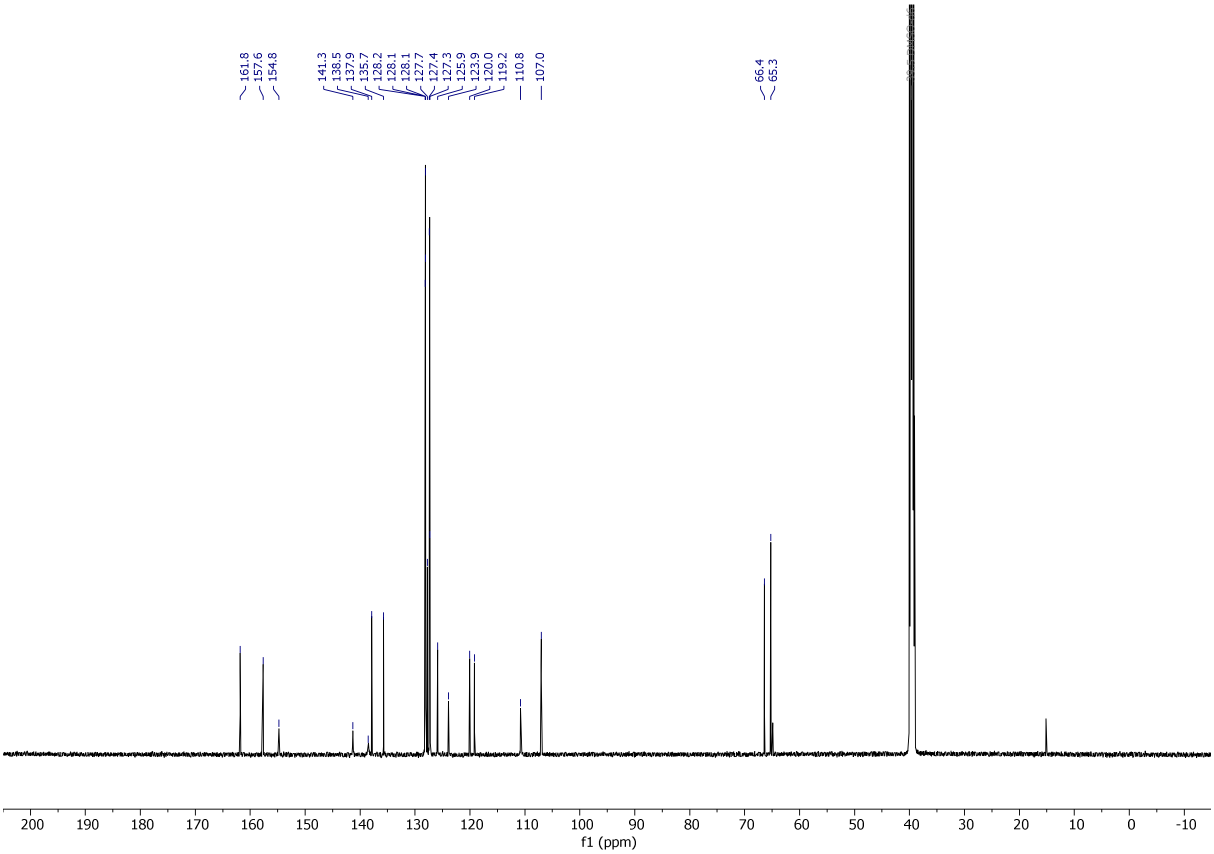

^{1}H NMR of **S4** (600 MHz, DMSO-d_6)

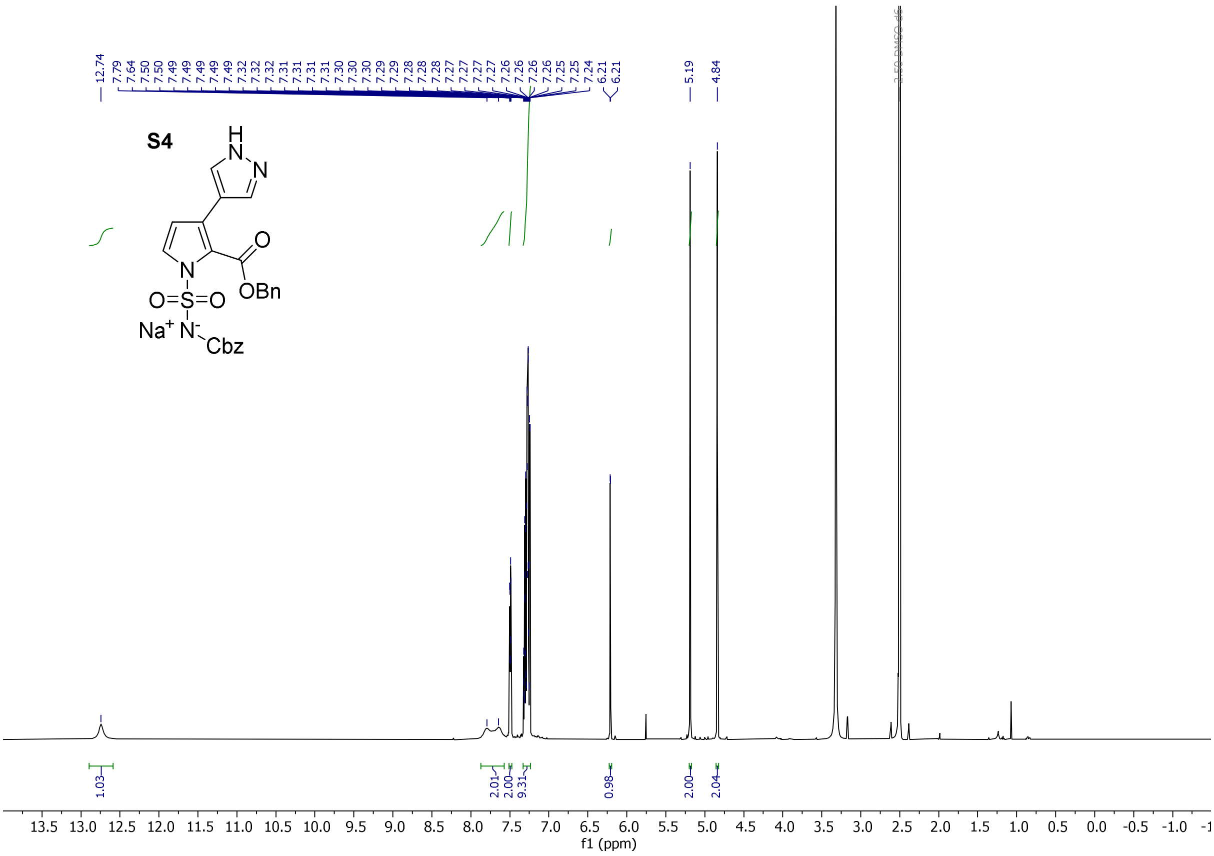

^{13}C NMR of **S4** (151 MHz, DMSO-d_6)

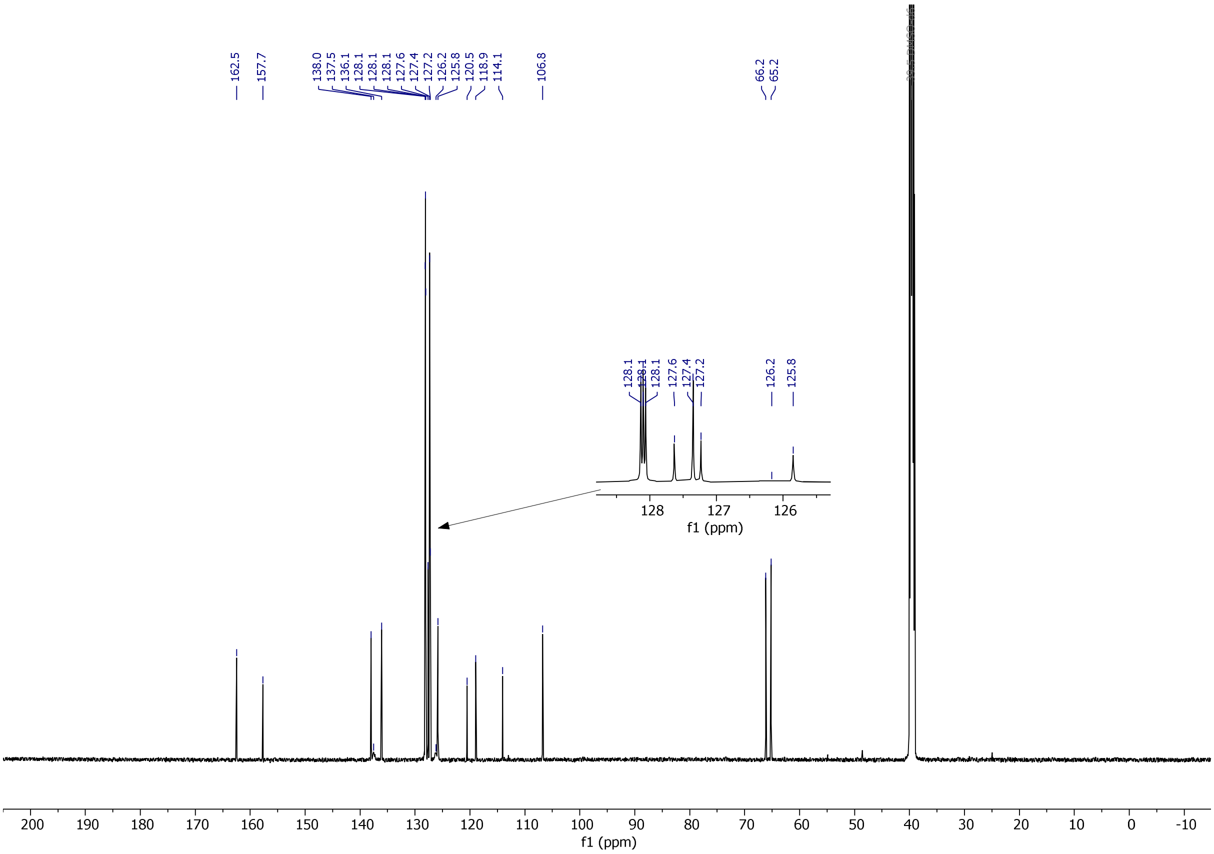

^{1}H NMR of **S5** (600 MHz, DMSO-d_6)

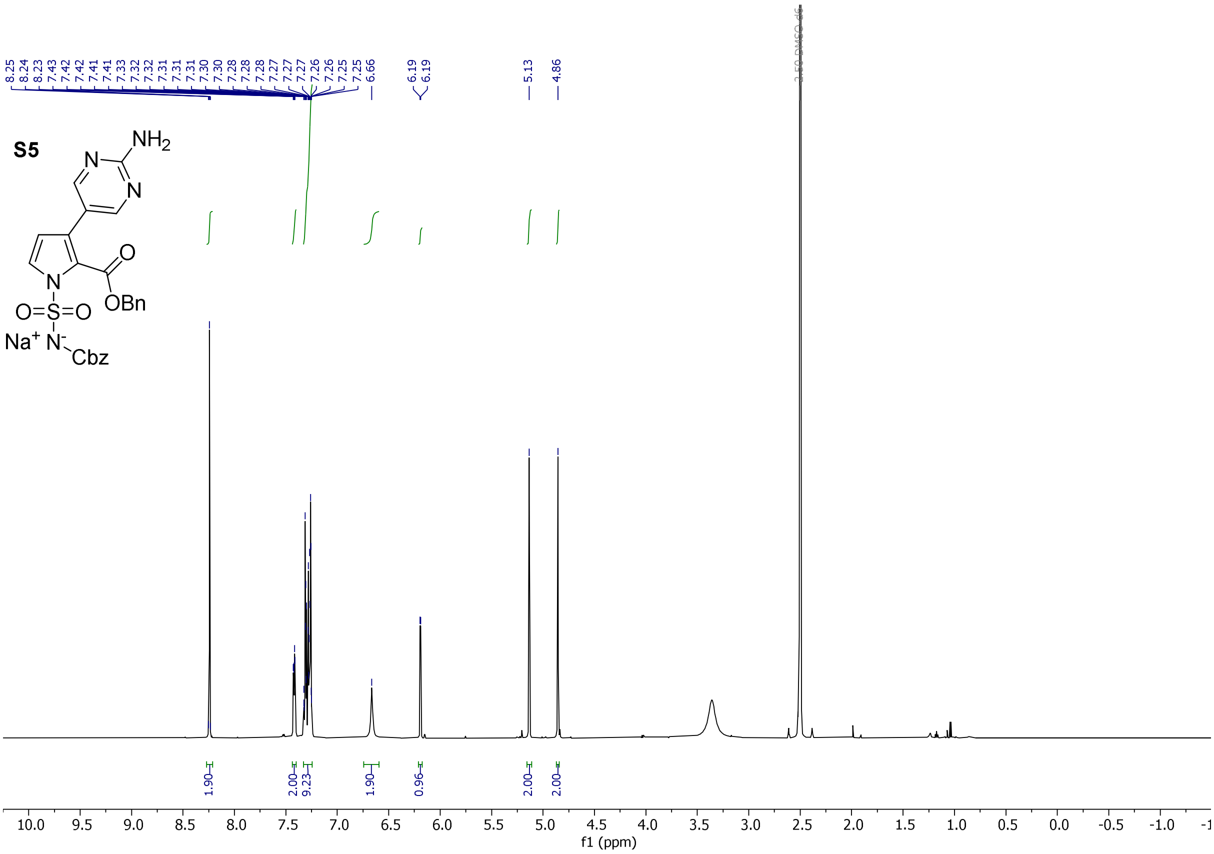

^{13}C NMR of **S5** (151 MHz, DMSO-d_6)

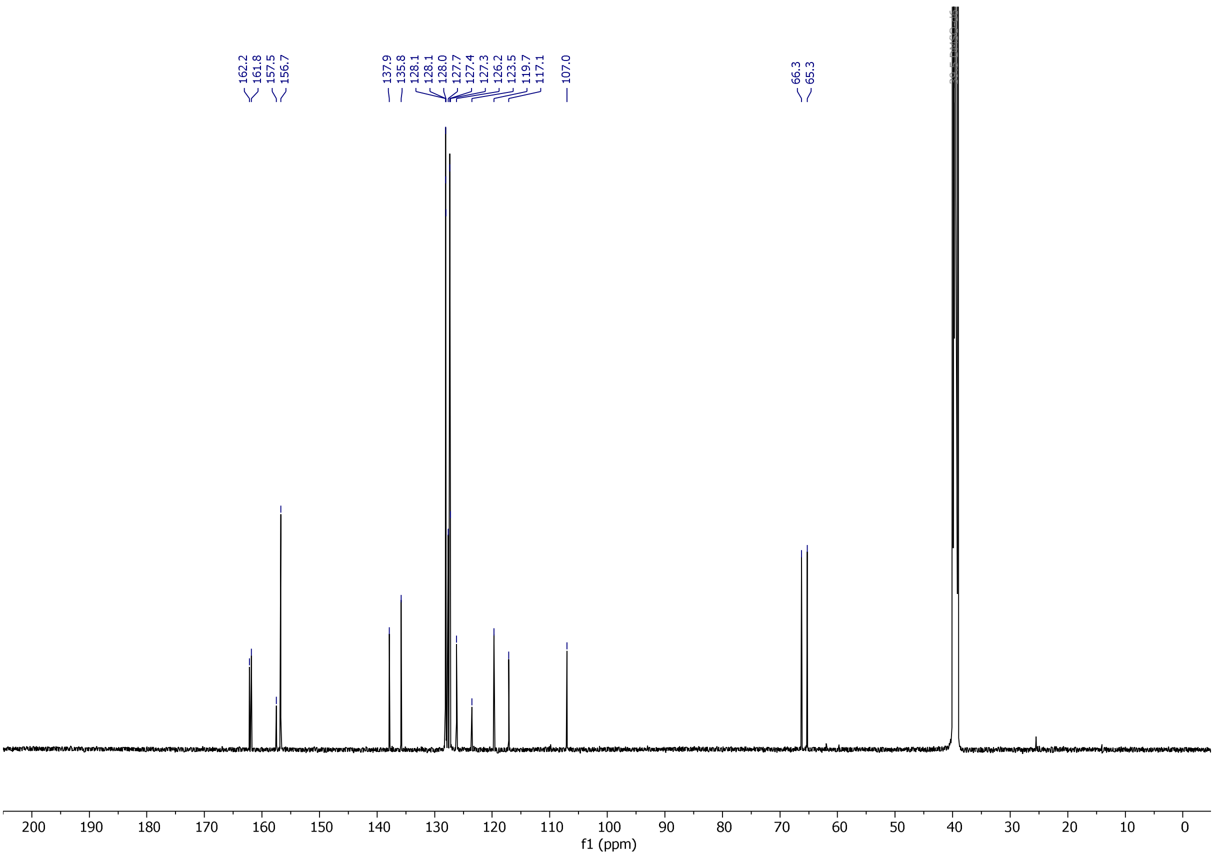

^{1}H NMR of **S6** (600 MHz, DMSO-d_6)

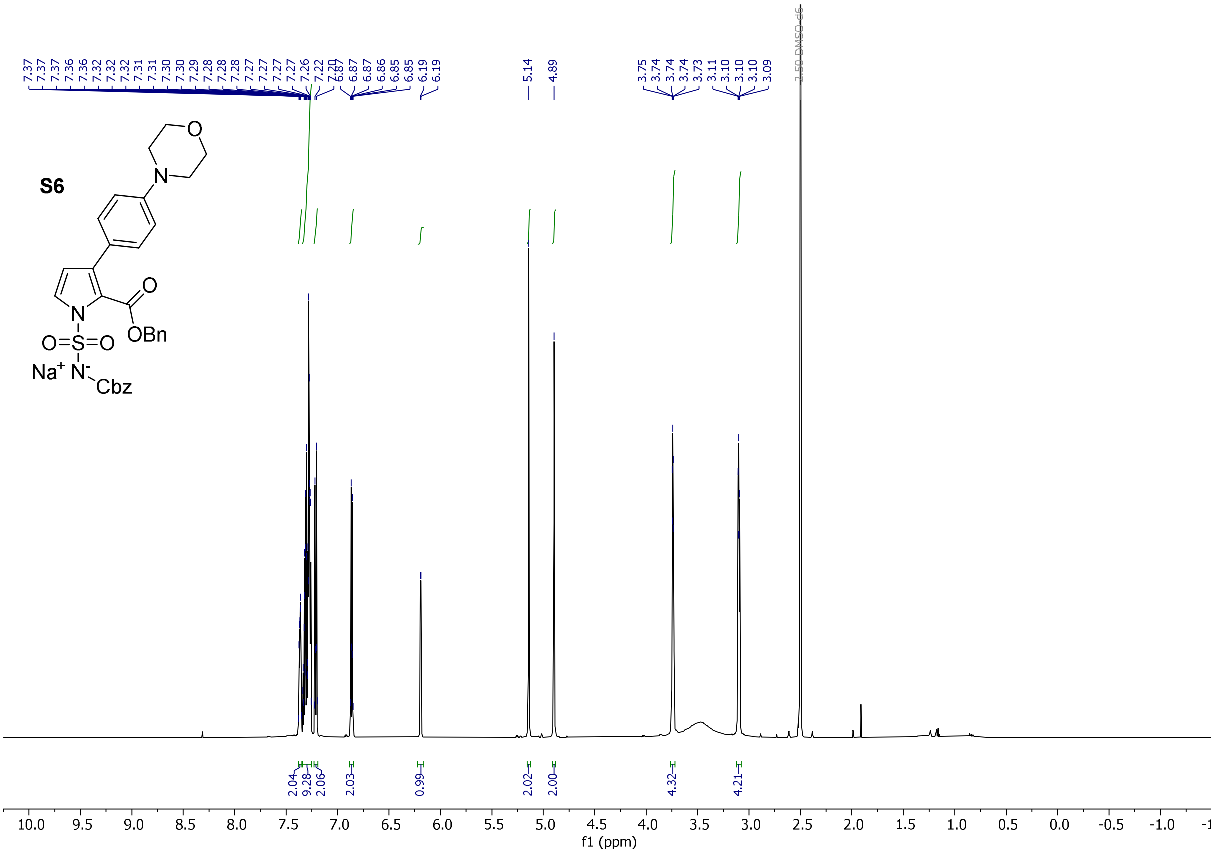

^{13}C NMR of **S6** (151 MHz, DMSO-d_6)

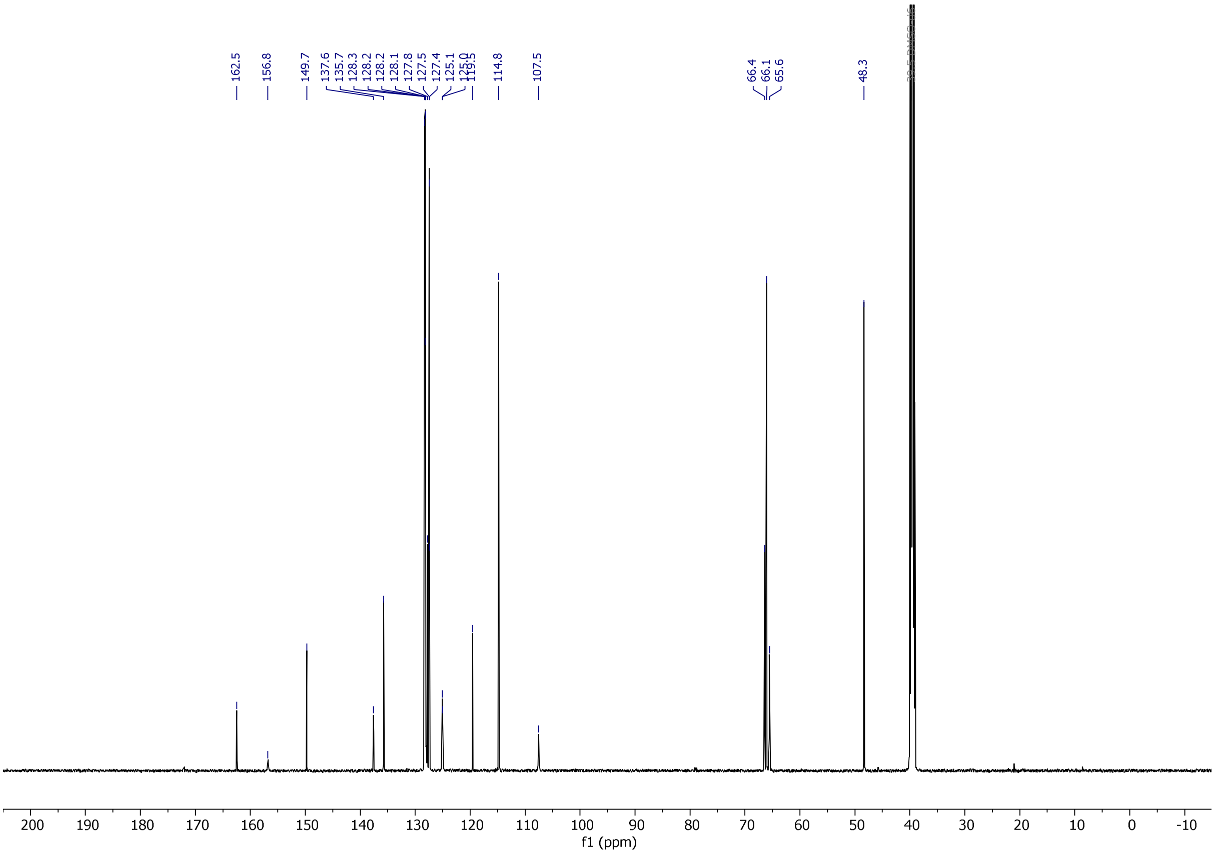

^{1}H NMR of **S7** (600 MHz, DMSO-d_6)

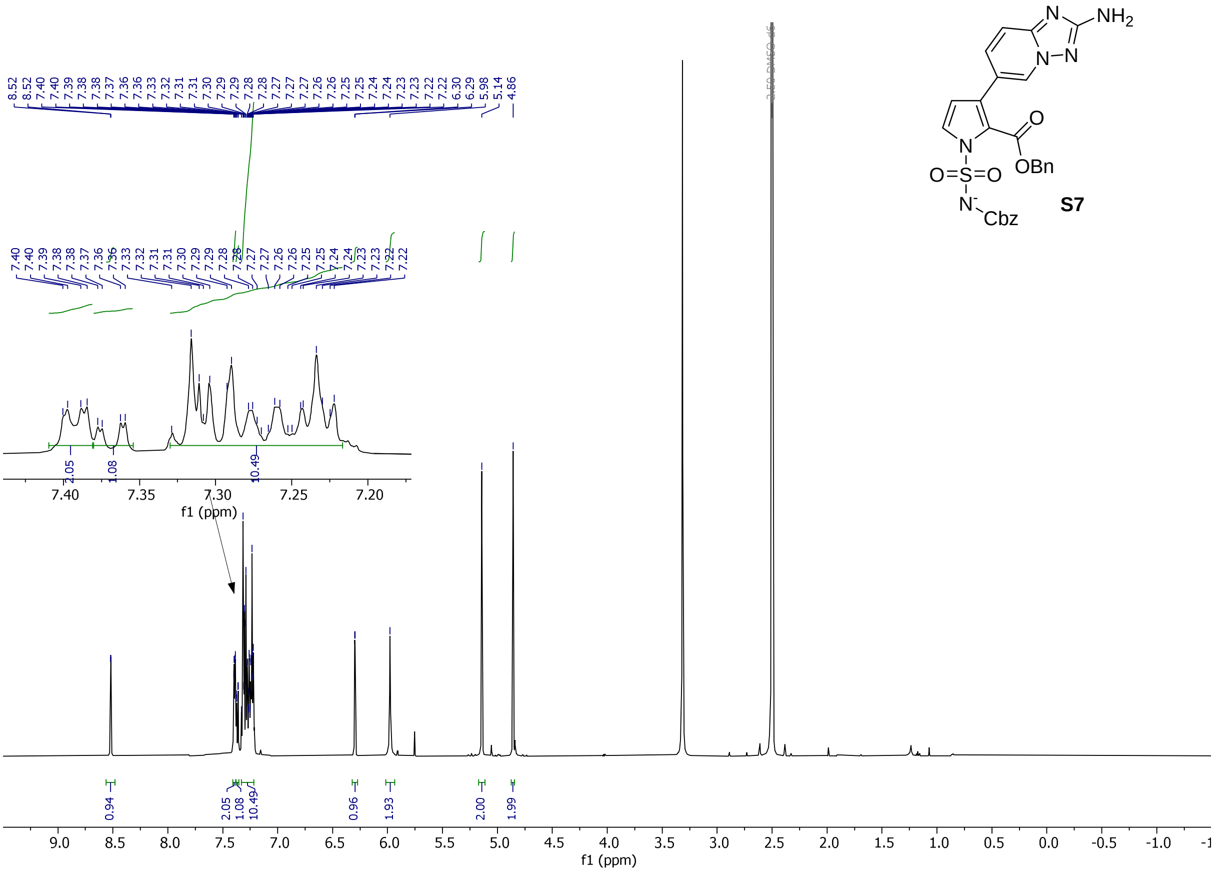

S7

^{13}C NMR of **S7** (151 MHz, DMSO-d_6)

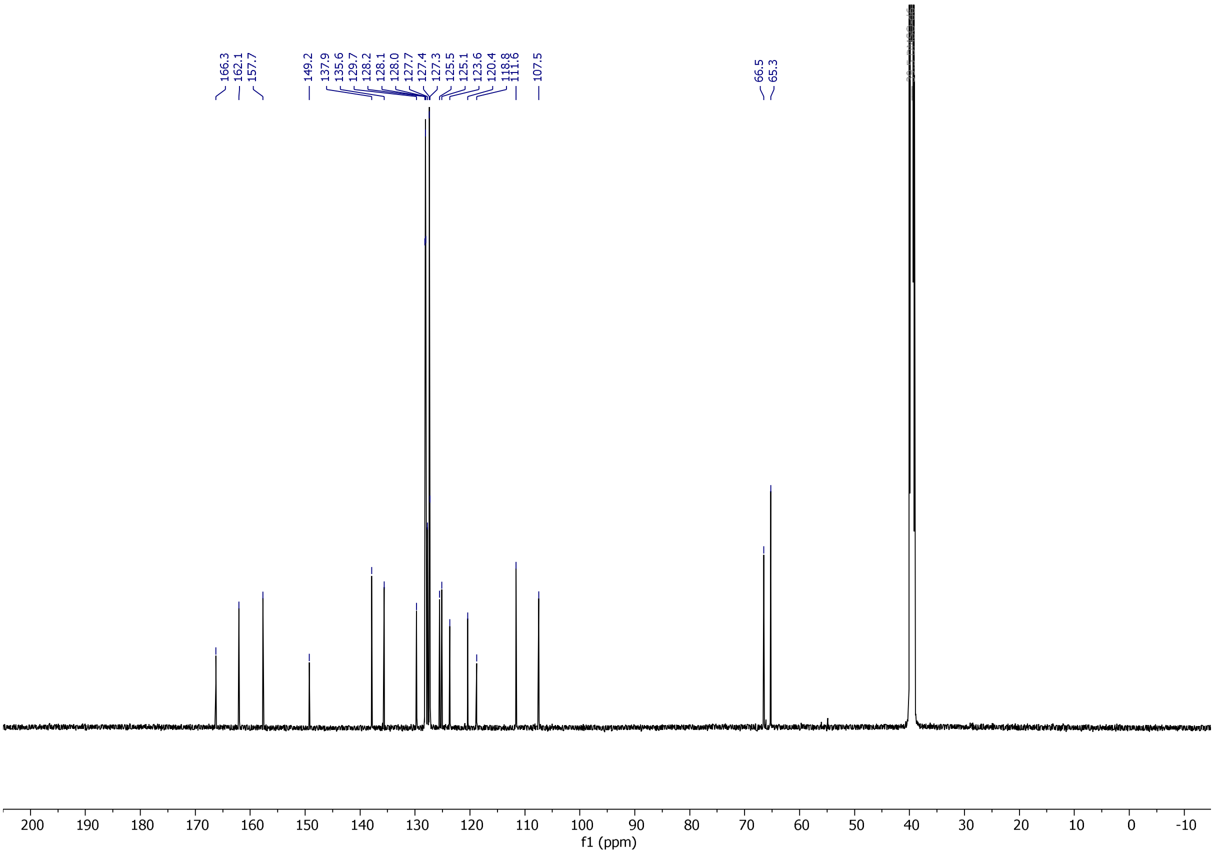

^{1}H NMR of **S8** (600 MHz, $CDCl_3$)

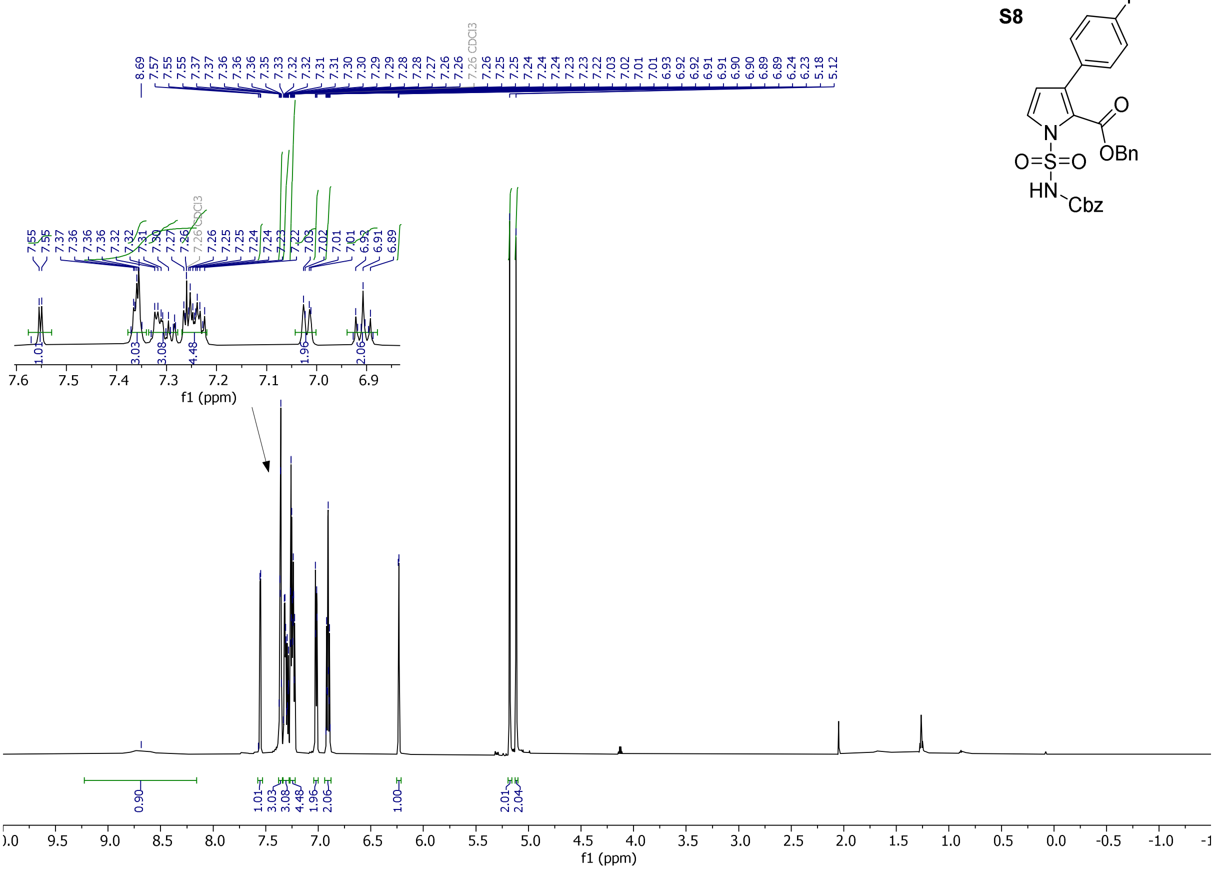

^{19}F NMR of **S8** (376 MHz, $CDCl_3$)

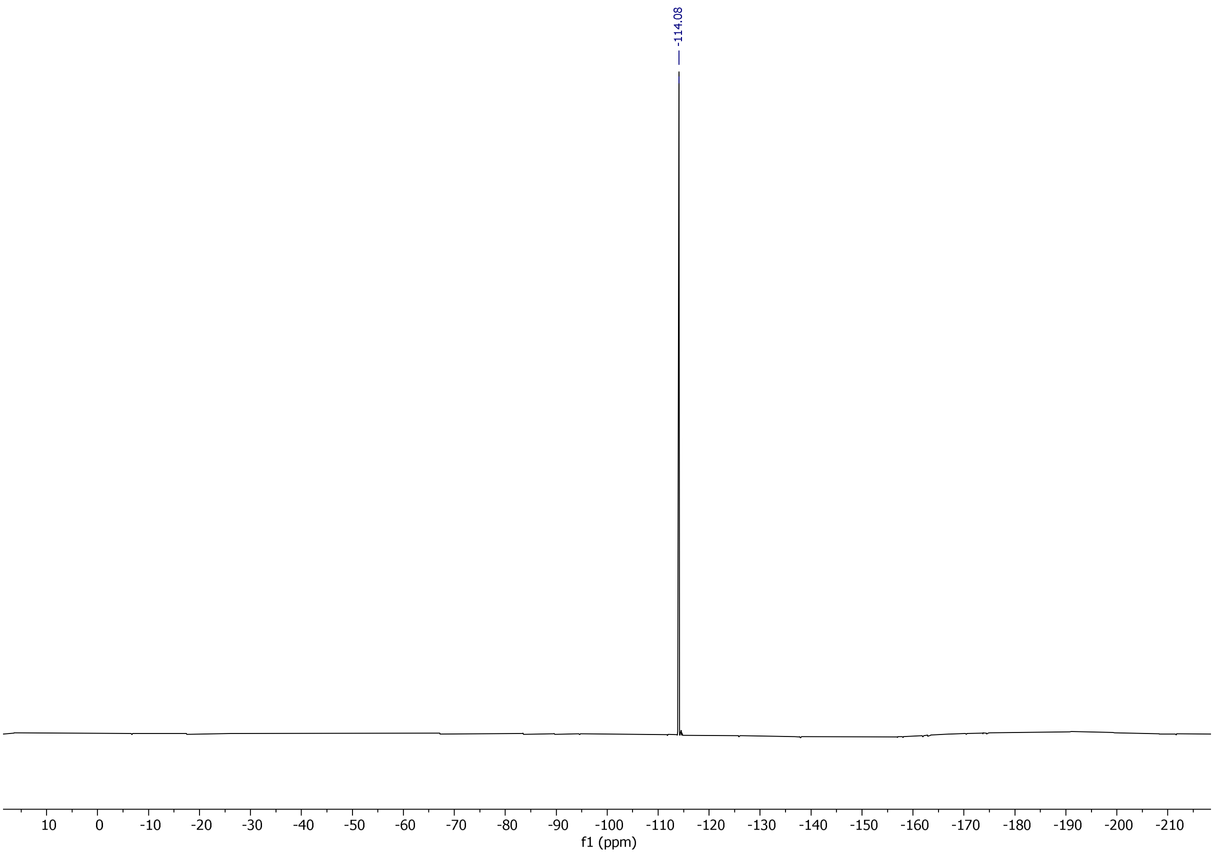

^{1}H NMR of **6a** (600 MHz, DMSO-d_6)

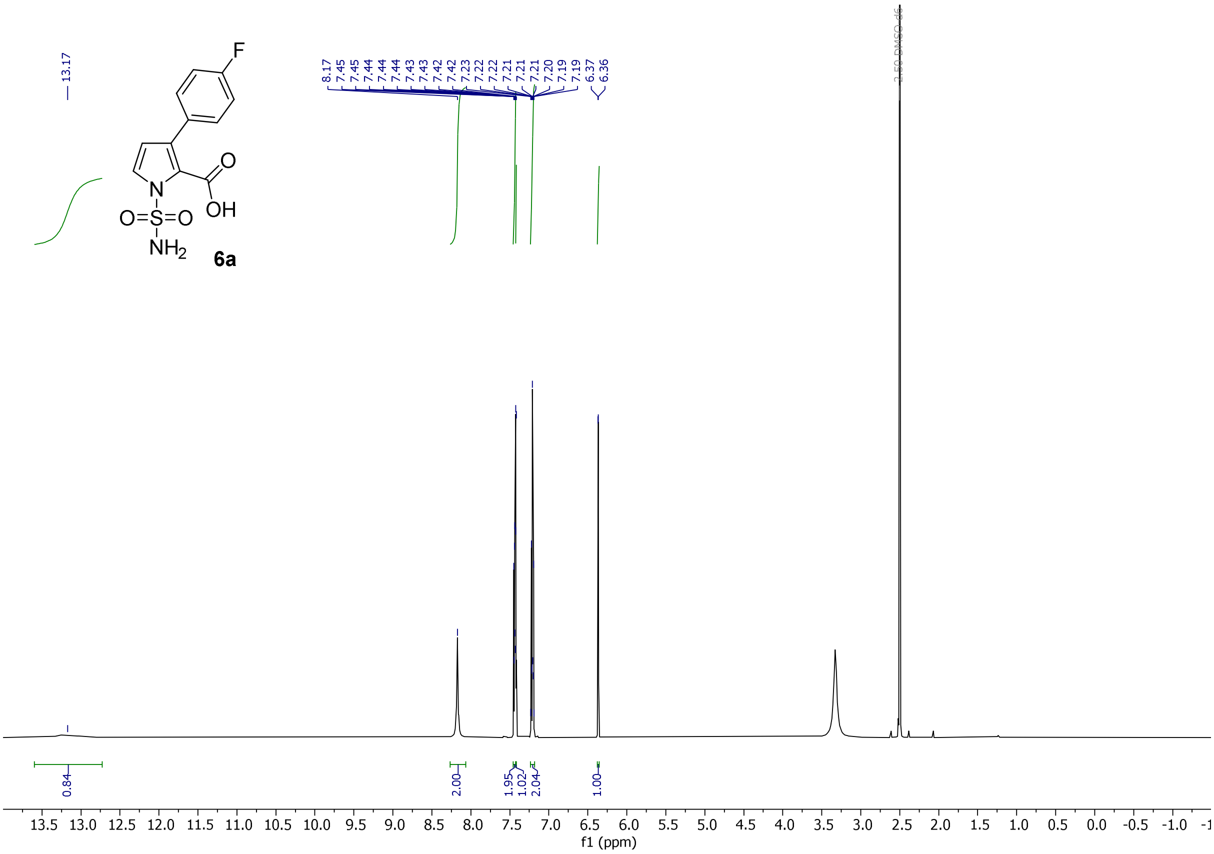

^{13}C NMR of **6a** (151 MHz, DMSO-d_6)

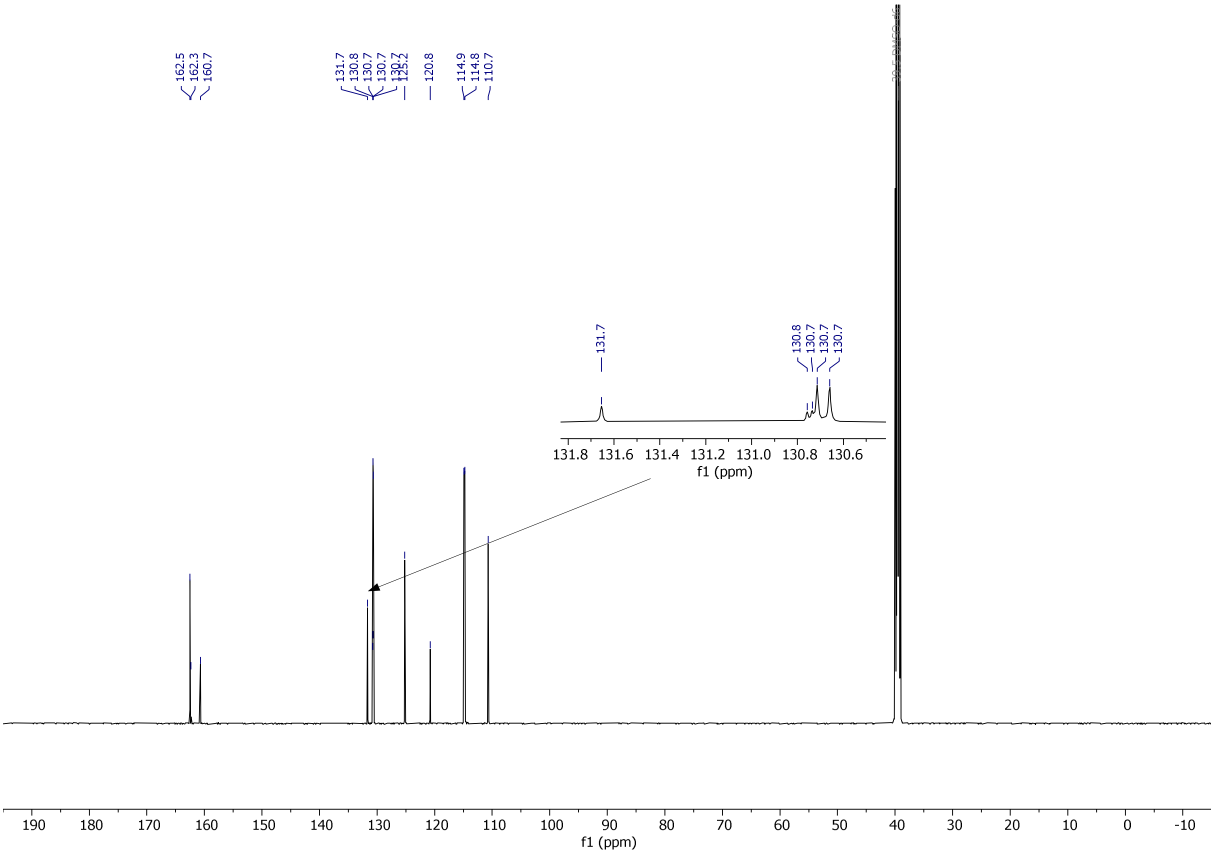

^{19}F NMR of **6a** (376 MHz, DMSO-d_6)

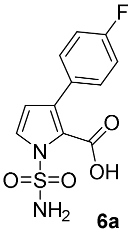

6a

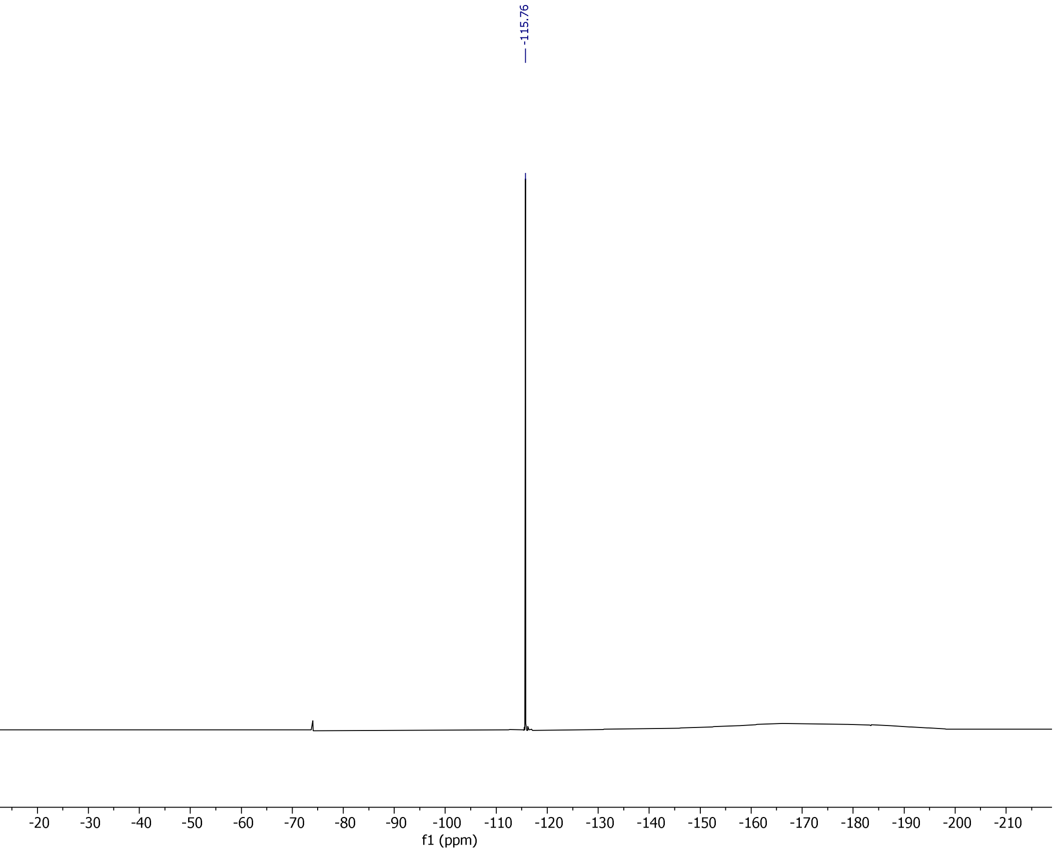

^{1}H NMR of **6b** (600 MHz, DMSO-d_6)

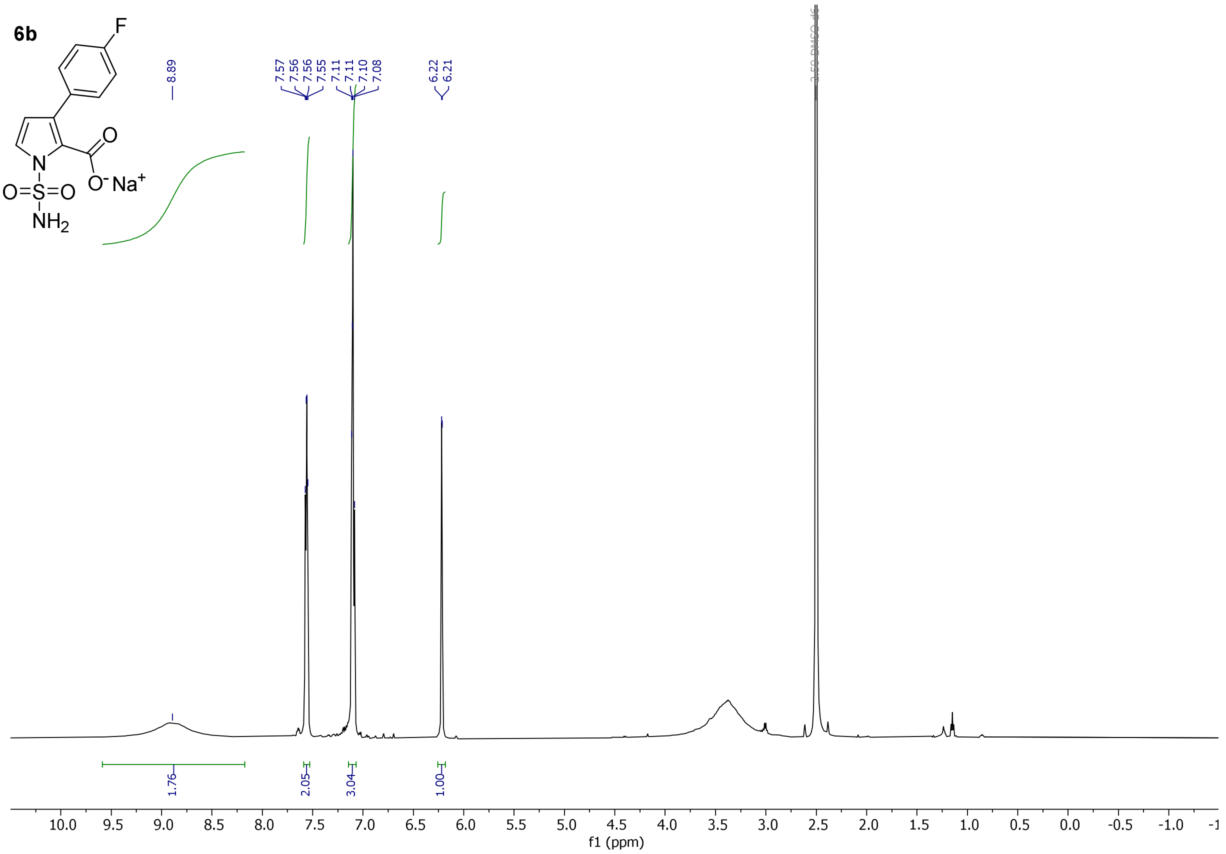

^{13}C NMR of **6b** (151 MHz, DMSO-d_6)

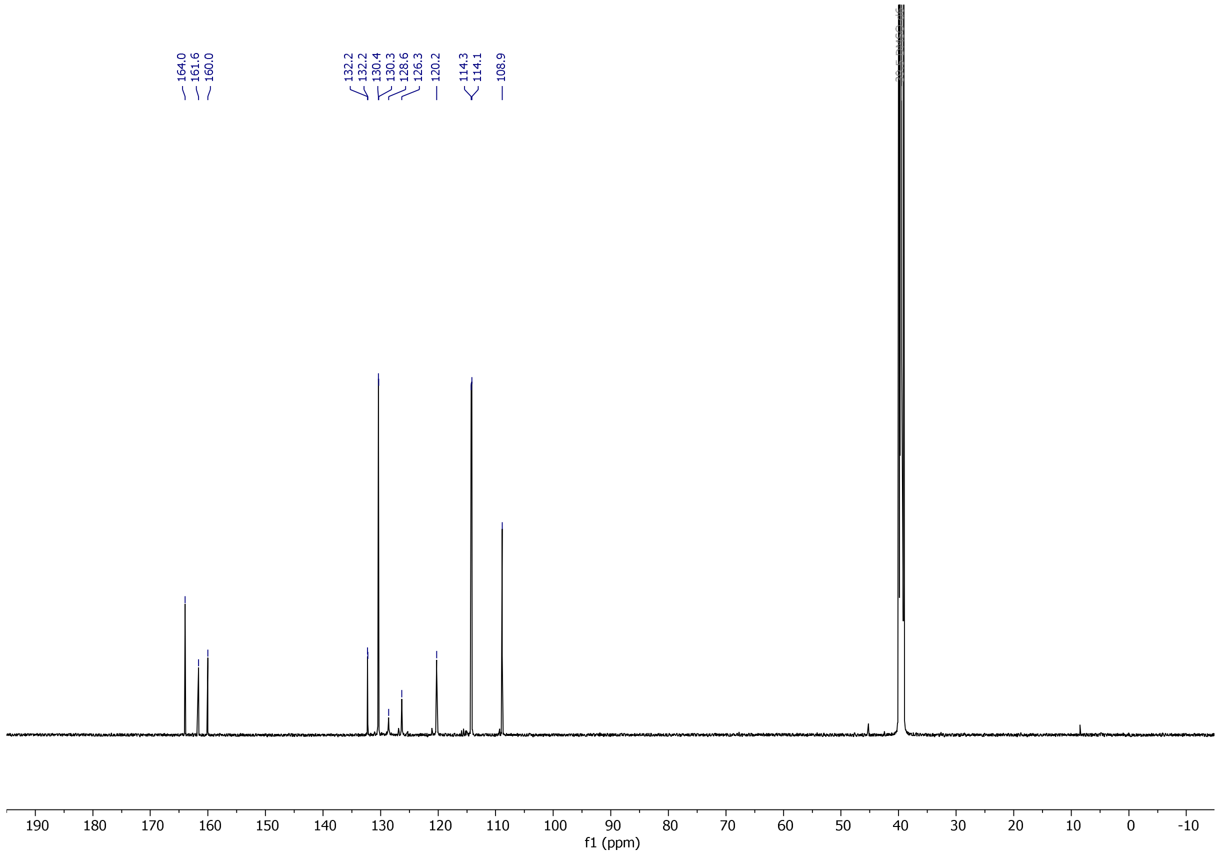

^{19}F NMR of **6b** (376 MHz, DMSO-d_6)

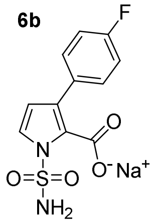

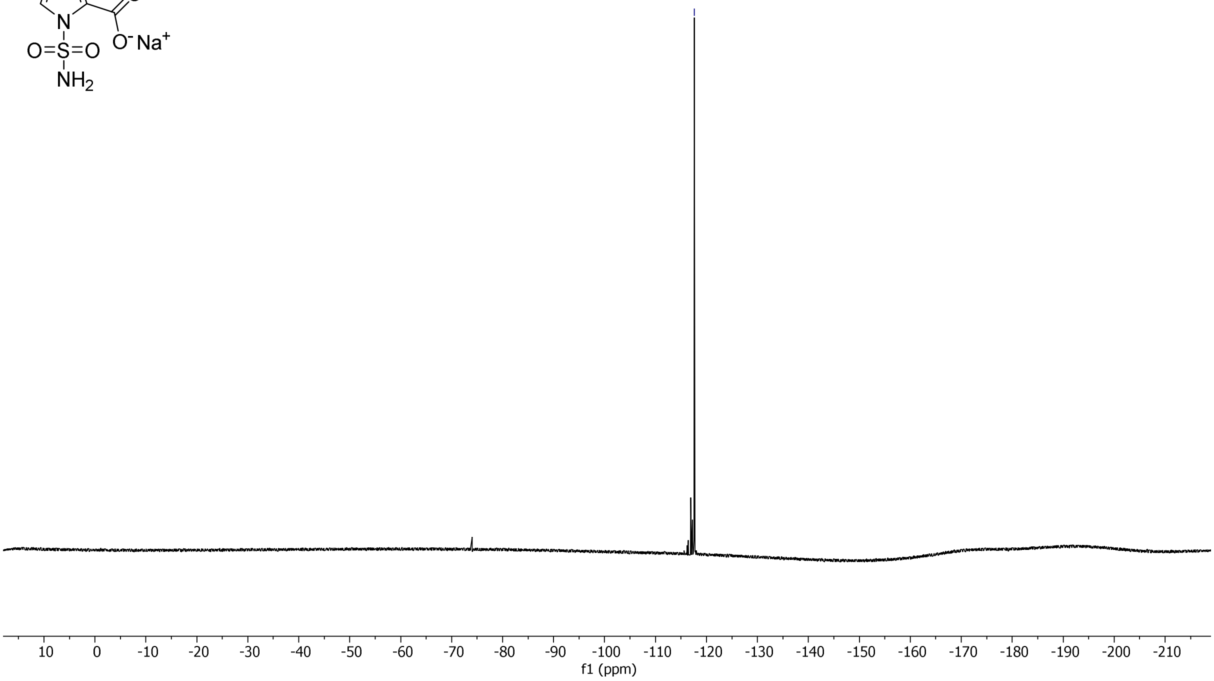

^{1}H NMR of **8** (600 MHz, DMSO-d_6)

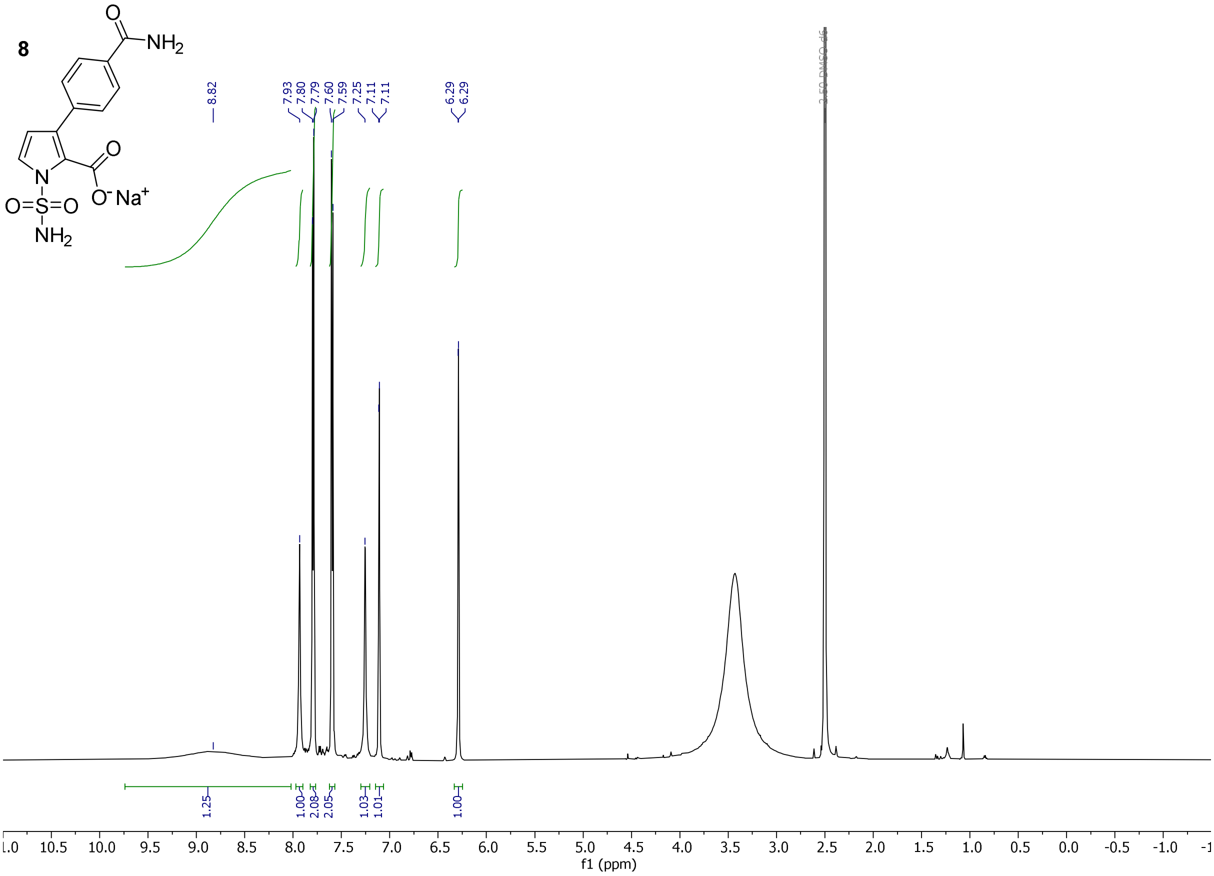

^{13}C NMR of **8** (151 MHz, DMSO-d_6)

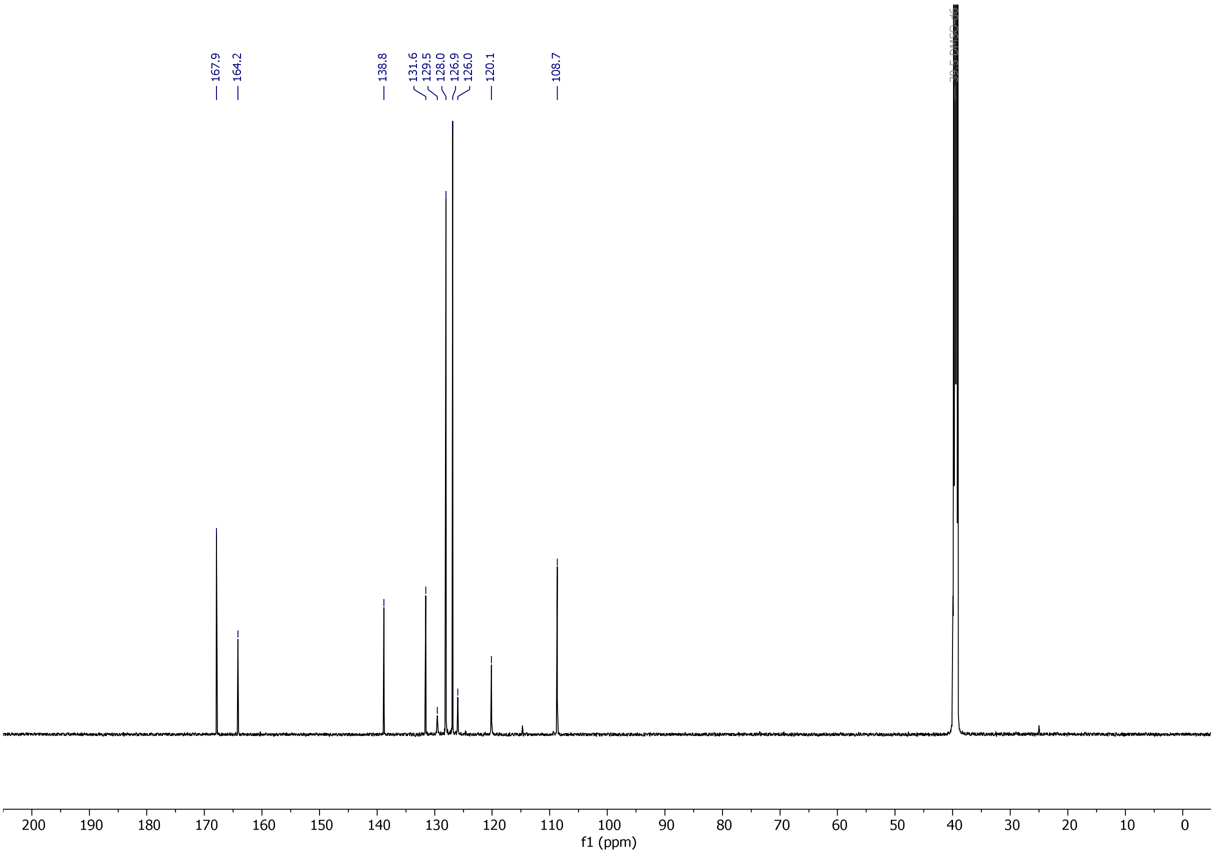

^{1}H NMR of **9** (600 MHz, DMSO-d_6)

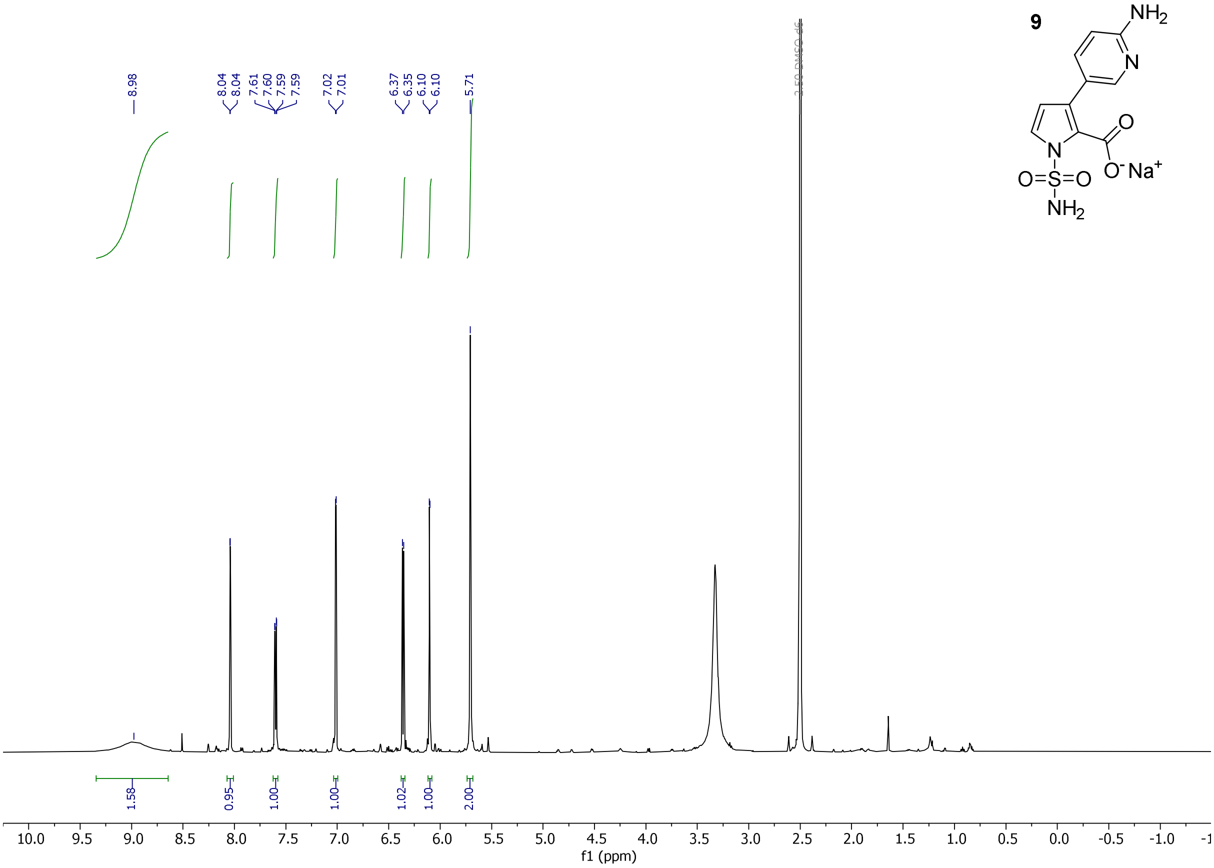

^{13}C NMR of **9** (151 MHz, DMSO-d_6)

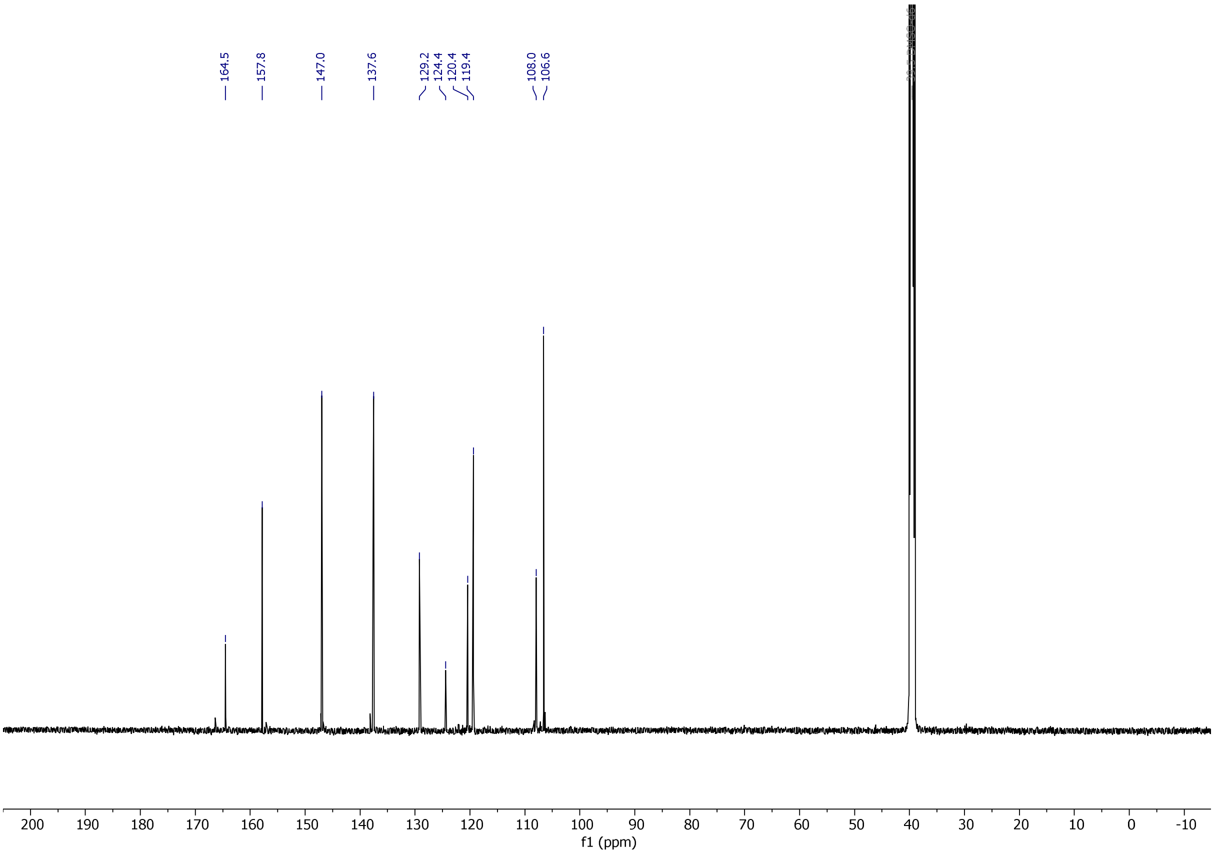

^{1}H NMR of **10** (600 MHz, DMSO-d_6)

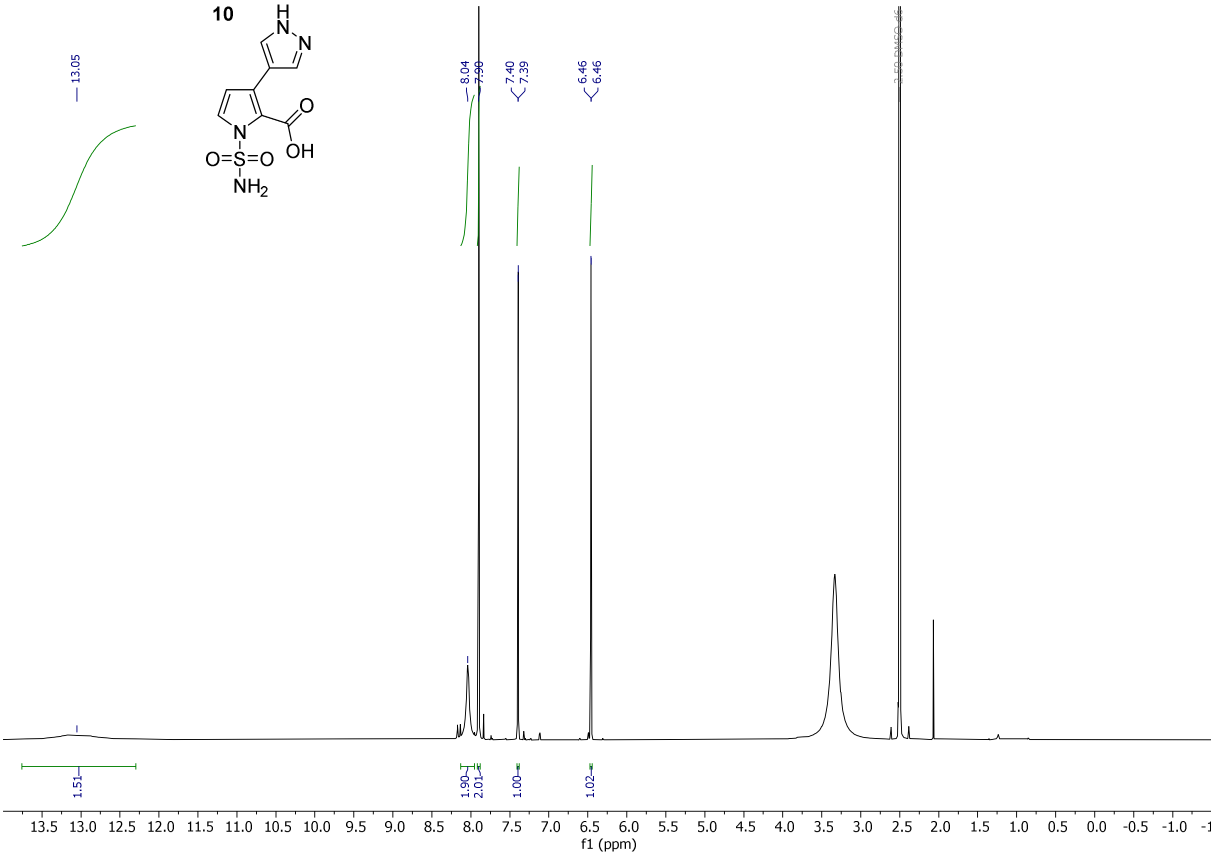

^{13}C NMR of **10** (151 MHz, DMSO-d_6)

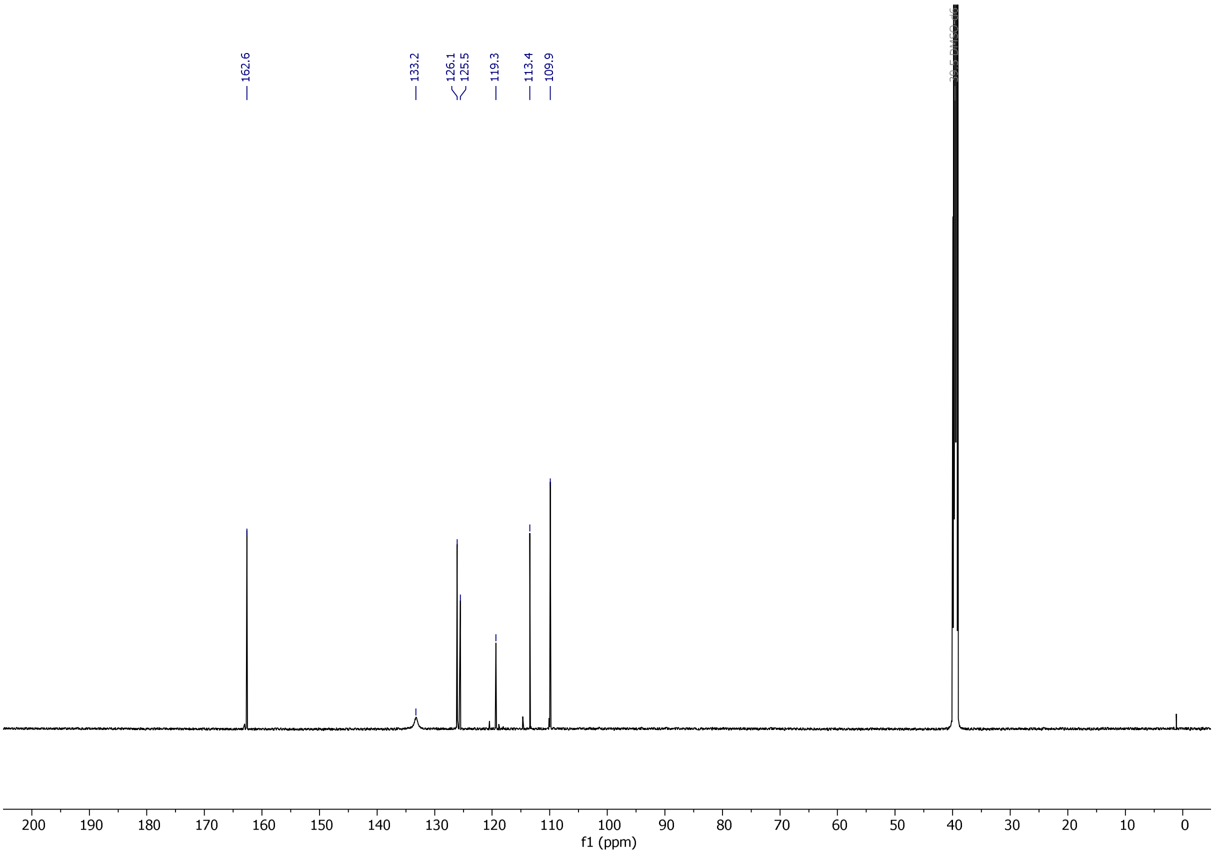

^{1}H NMR of **11** (600 MHz, DMSO-d_6)

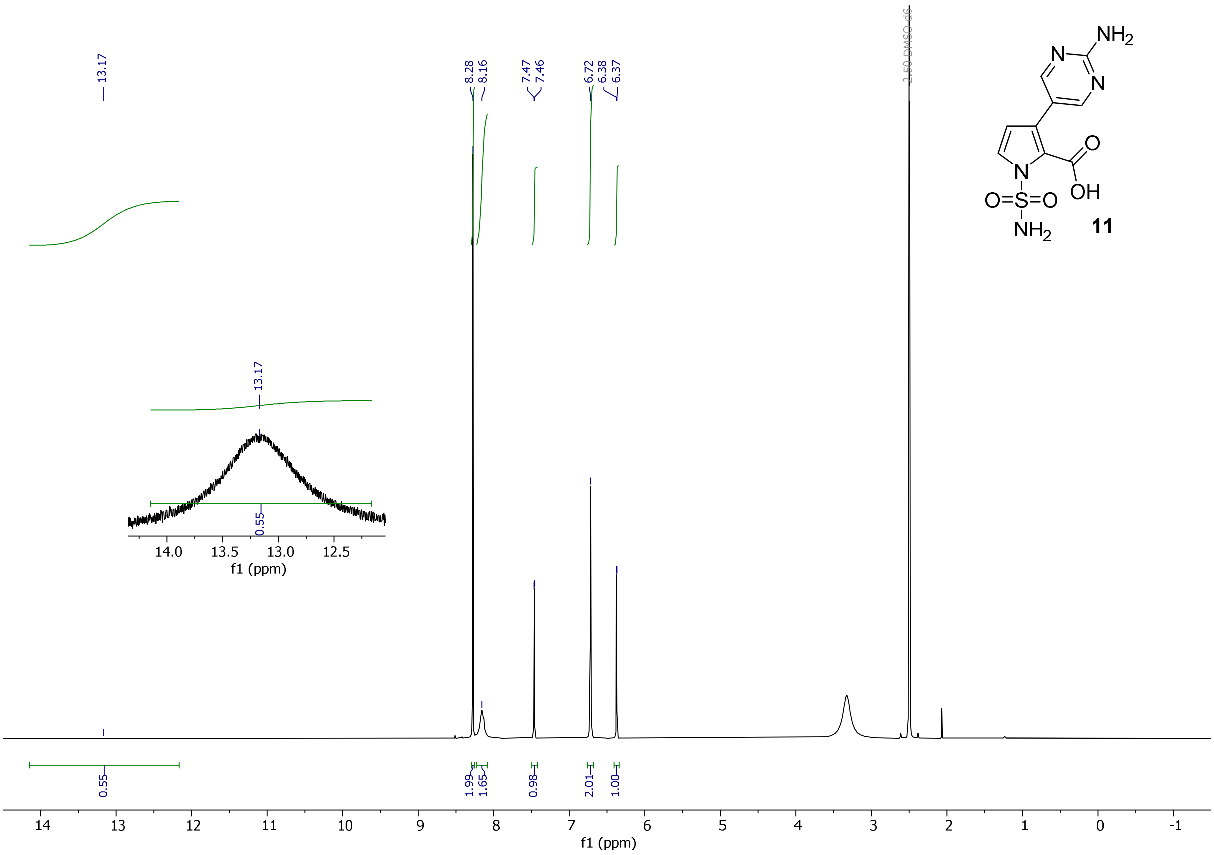

^{13}C NMR of **11** (151 MHz, DMSO-d_6)

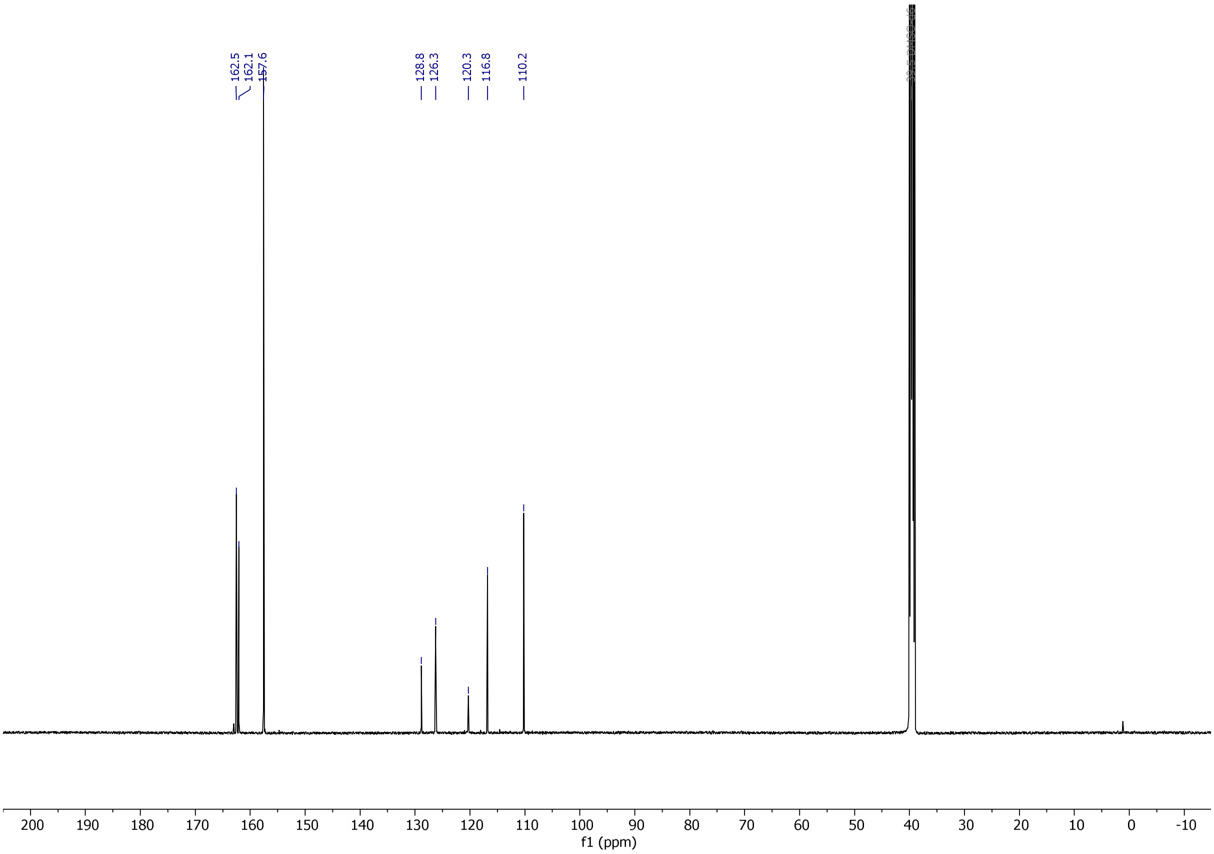

^{1}H NMR of **12** (600 MHz, DMSO-d_6)

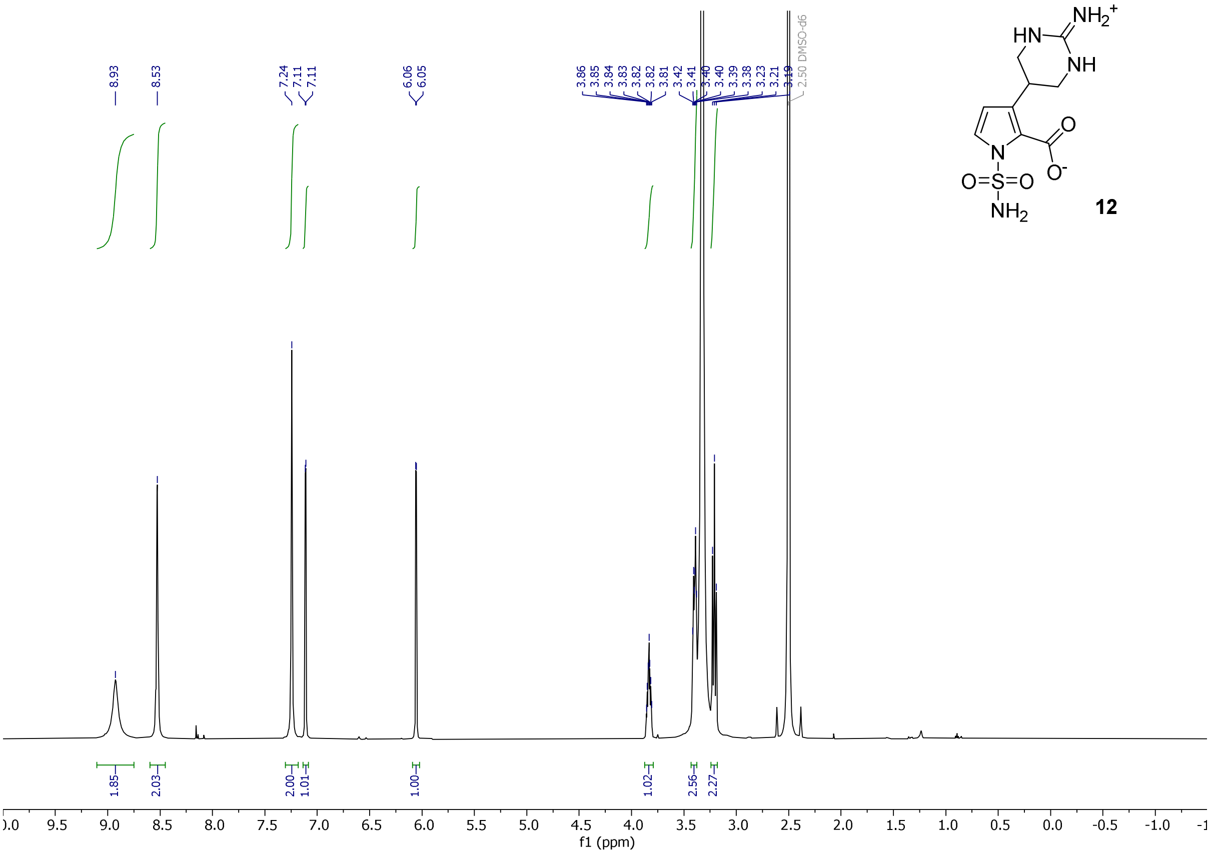

12

^{13}C NMR of **12** (151 MHz, DMSO-d_6)

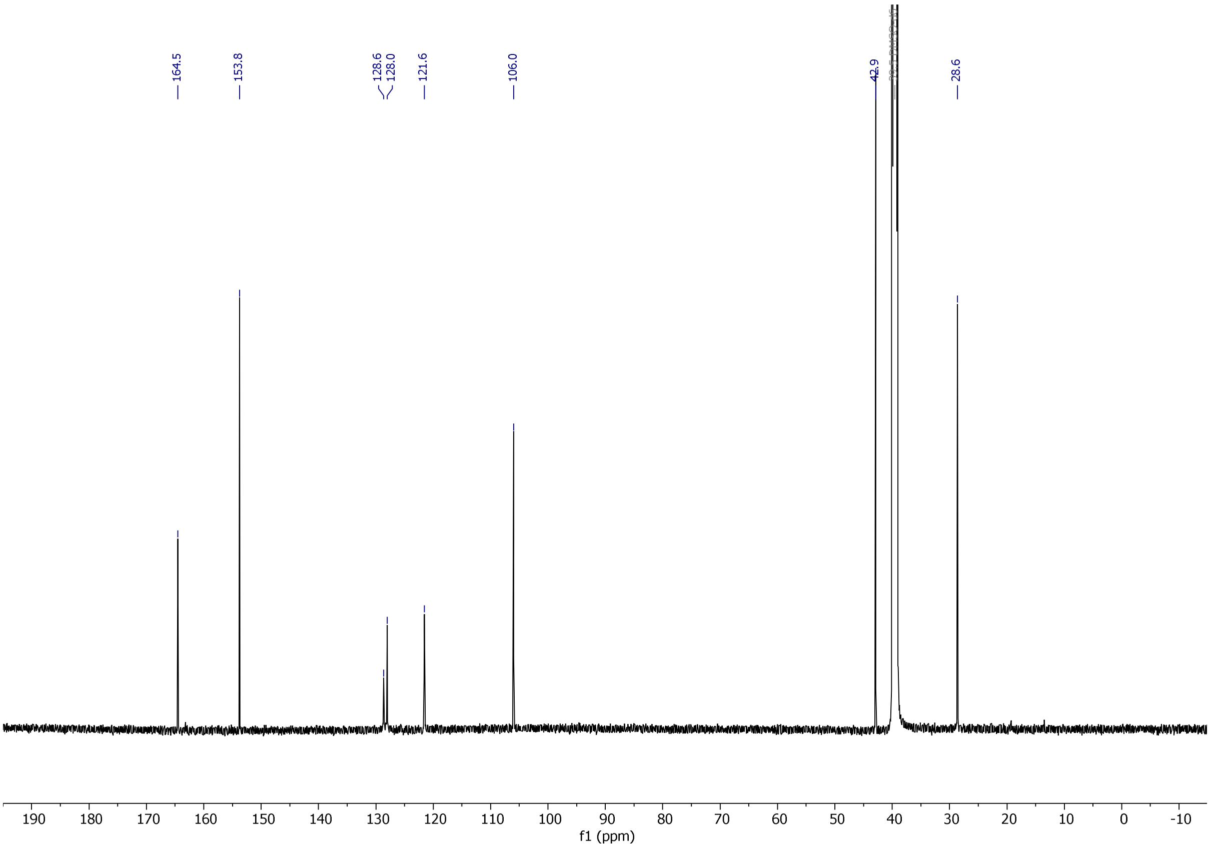

^{1}H NMR of **13** (600 MHz, DMSO-d_6)

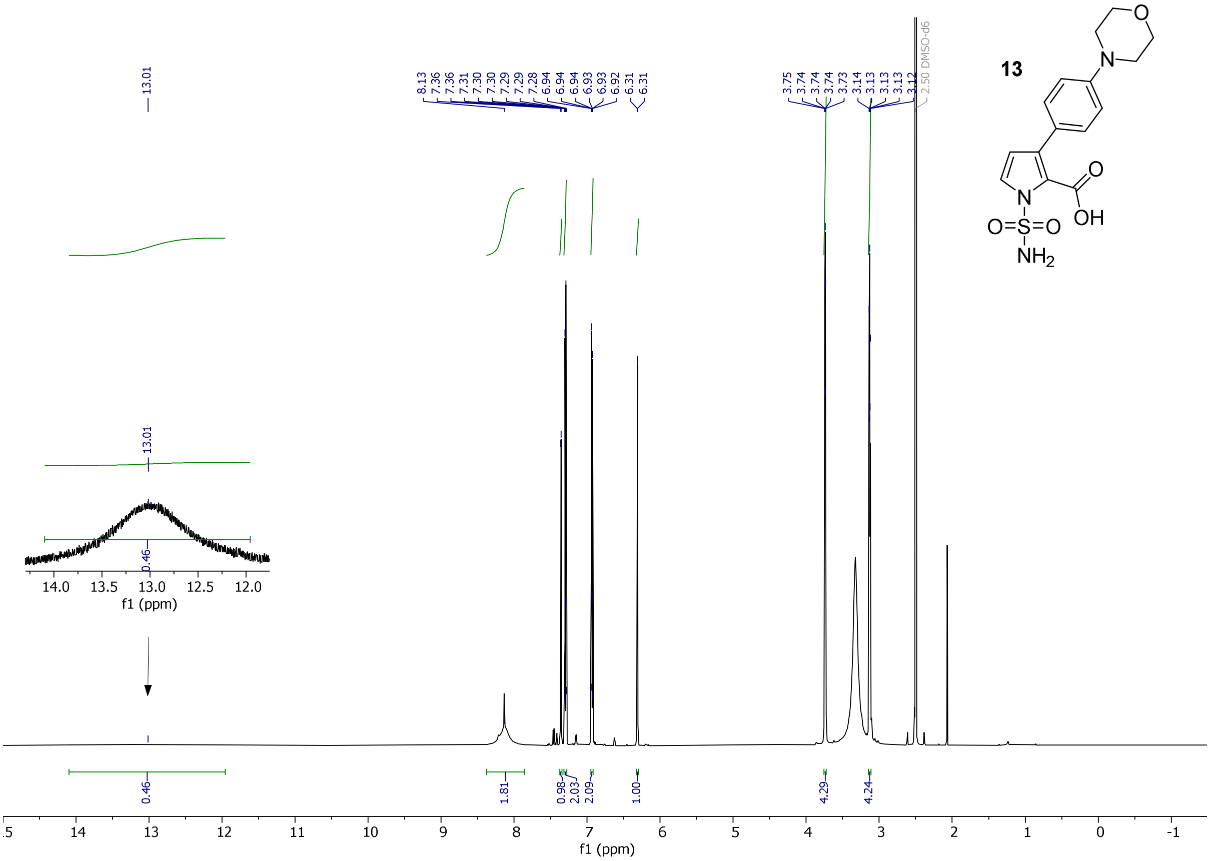

^{13}C NMR of **13** (151 MHz, DMSO-d_6)

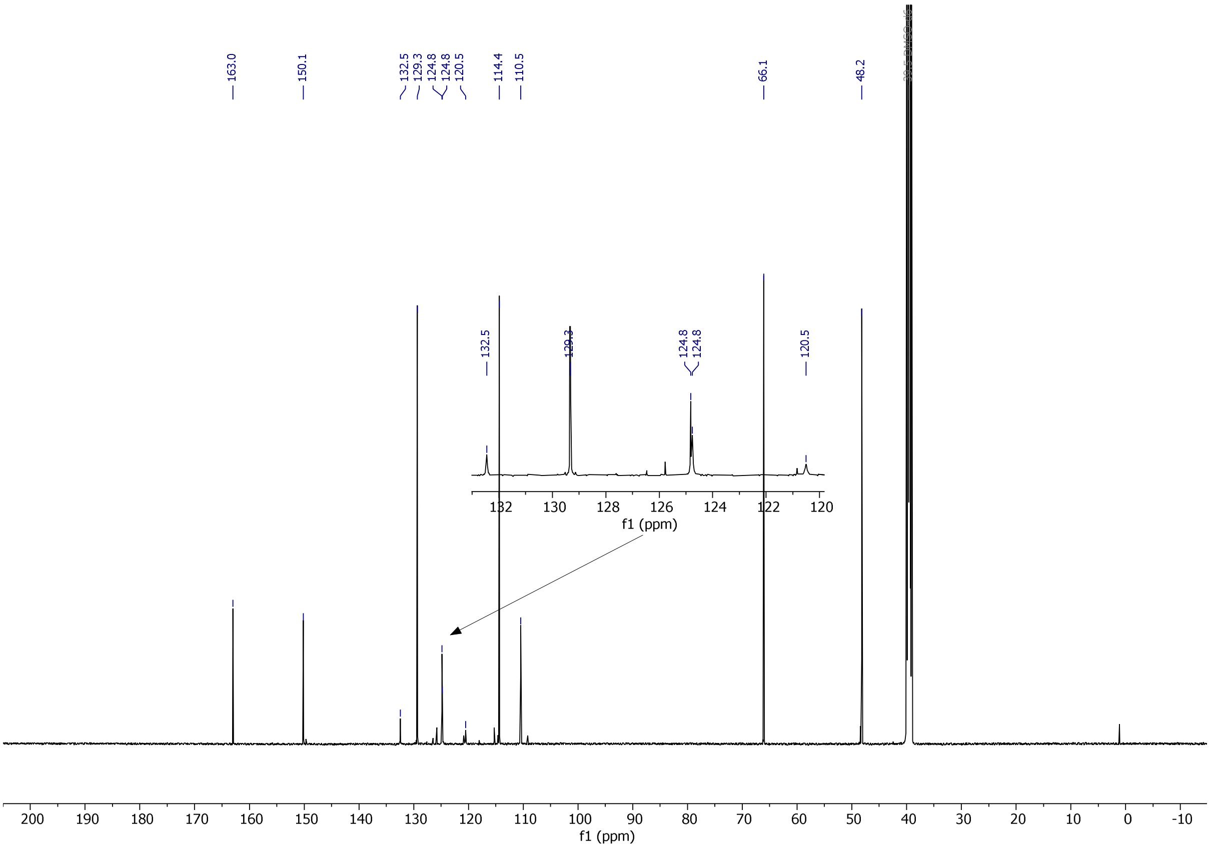

^{1}H NMR of **14** (600 MHz, DMSO-d_6)

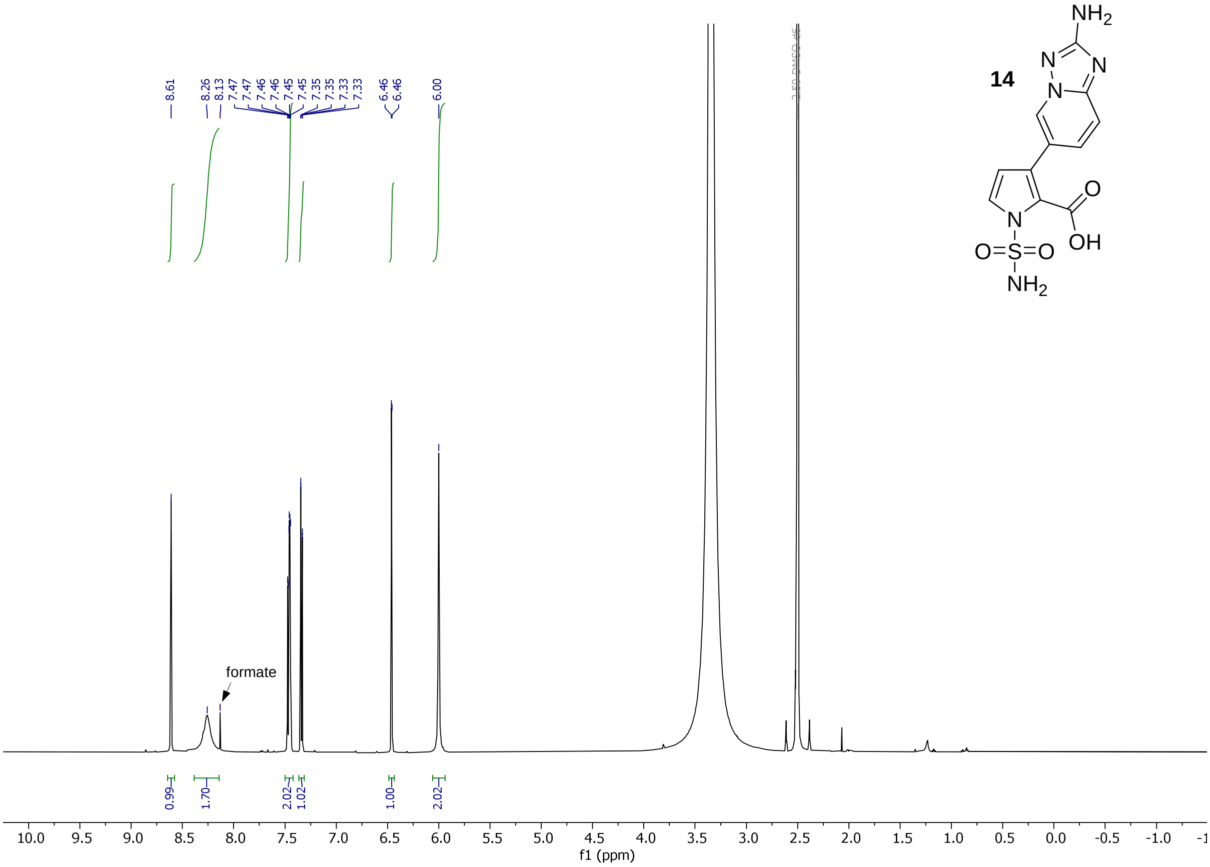

^{13}C NMR of **14** (151 MHz, DMSO-d_6)

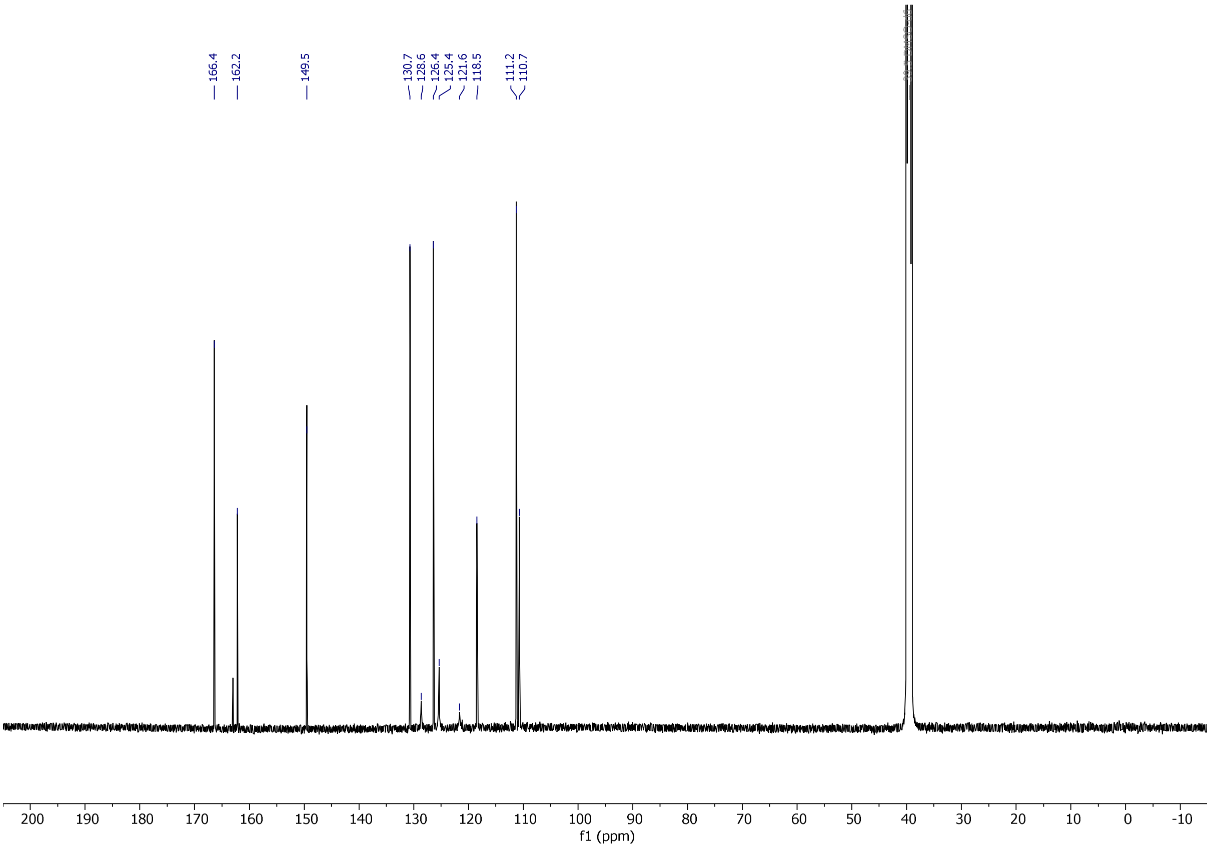

6 HPLC chromatograms of final compounds

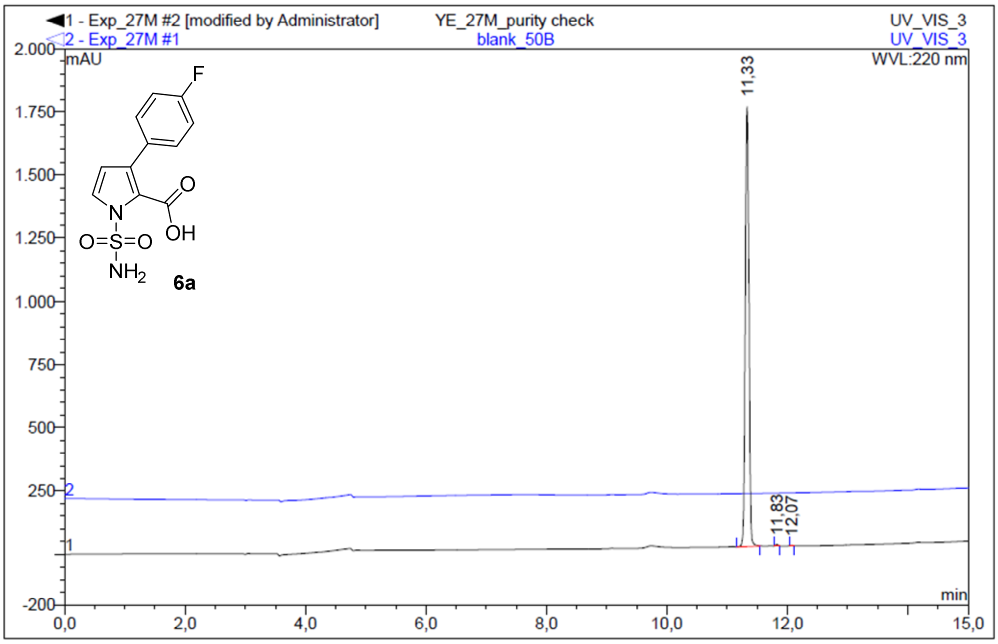

No.	Ret.Time min	Peak Name	Height mAU	Area mAU*min	Rel.Area %	Amount	Resolution(EP)
1	11,33	n.a.	1741,375	122,208	99,69	n.a.	5,14
2	11,83	n.a.	5,773	0,272	0,22	n.a.	3,18
3	12,07	n.a.	2,333	0,104	0,08	n.a.	n.a.
Total:			1749,481	122,583	100,00	0,000	

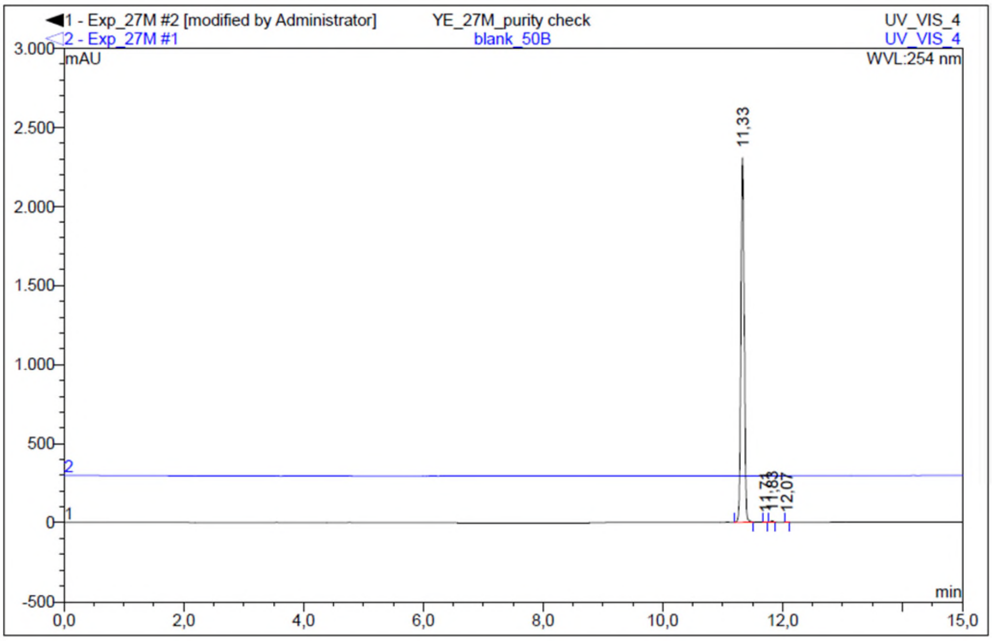

No.	Ret.Time min	Peak Name	Height mAU	Area mAU*min	Rel.Area %	Amount	Resolution(EP)
1	11,33	n.a.	2305,923	146,034	99,61	n.a.	4,50
2	11,71	n.a.	1,392	0,055	0,04	n.a.	1,47
3	11,83	n.a.	8,682	0,450	0,31	n.a.	3,26
4	12,07	n.a.	1,793	0,068	0,05	n.a.	n.a.
Total:			2317,789	146,606	100,00	0,000	

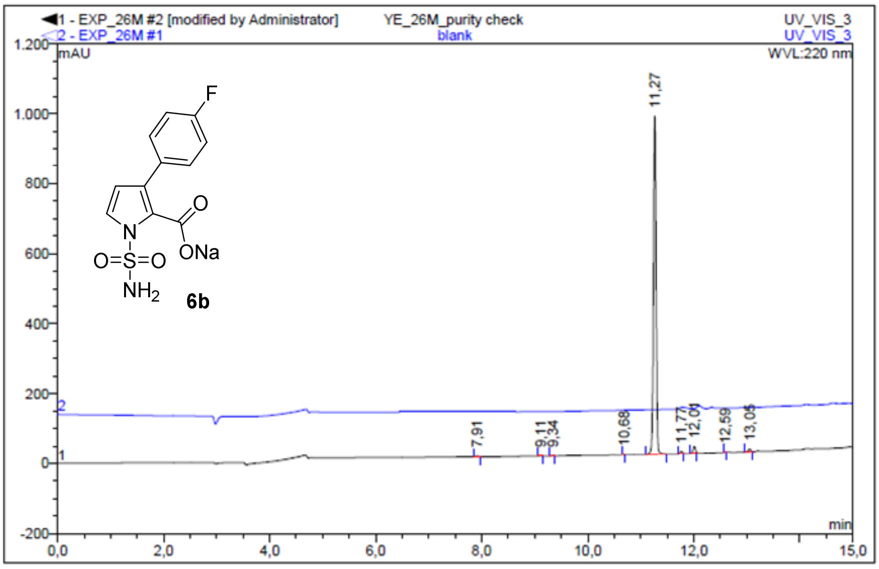

No.	Ret.Time min	Peak Name	Height mAU	Area mAU*min	Rel.Area %	Amount	Resolution(EP)
1	7,91	n.a.	2,277	0,136	0,23	n.a.	12,80
2	9,11	n.a.	0,618	0,033	0,06	n.a.	2,73
3	9,34	n.a.	0,831	0,040	0,07	n.a.	19,69
4	10,68	n.a.	0,255	0,008	0,01	n.a.	7,93
5	11,27	n.a.	967,257	57,061	96,54	n.a.	5,68
6	11,77	n.a.	6,710	0,341	0,58	n.a.	2,84
7	12,01	n.a.	19,521	1,047	1,77	n.a.	8,68
8	12,59	n.a.	0,488	0,013	0,02	n.a.	6,66
9	13,05	n.a.	7,623	0,429	0,73	n.a.	n.a.
Total:			1005,581	59,108	100,00	0,000	

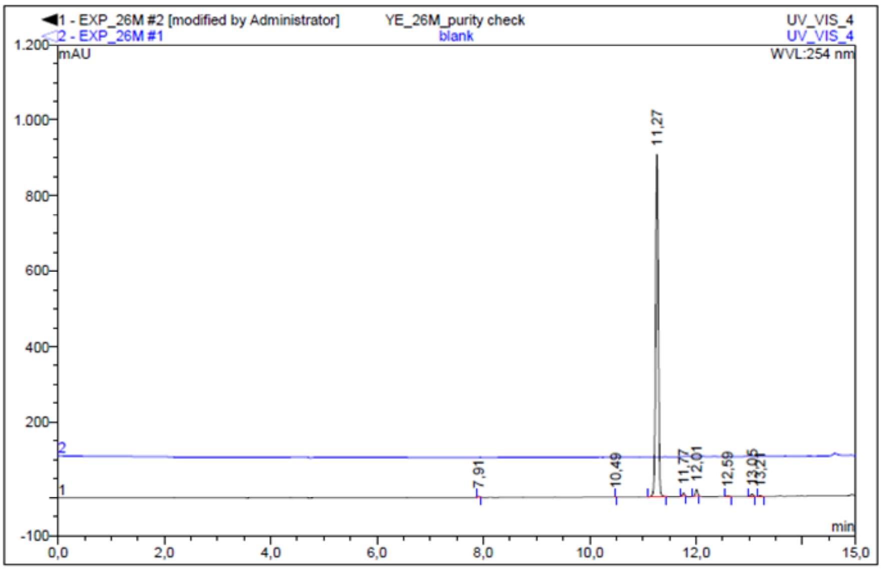

No.	Ret.Time min	Peak Name	Height mAU	Area mAU*min	Rel.Area %	Amount	Resolution(EP)
1	7,91	n.a.	2,950	0,148	0,27	n.a.	47,26
2	10,49	n.a.	0,011	0,000	0,00	n.a.	13,54
3	11,27	n.a.	907,329	53,327	96,36	n.a.	5,83
4	11,77	n.a.	8,363	0,398	0,72	n.a.	2,97
5	12,01	n.a.	17,878	0,924	1,67	n.a.	6,95
6	12,59	n.a.	1,756	0,089	0,16	n.a.	5,27
7	13,05	n.a.	6,006	0,339	0,61	n.a.	1,75
8	13,21	n.a.	2,204	0,118	0,21	n.a.	n.a.
Total:			946,497	55,343	100,00	0,000	

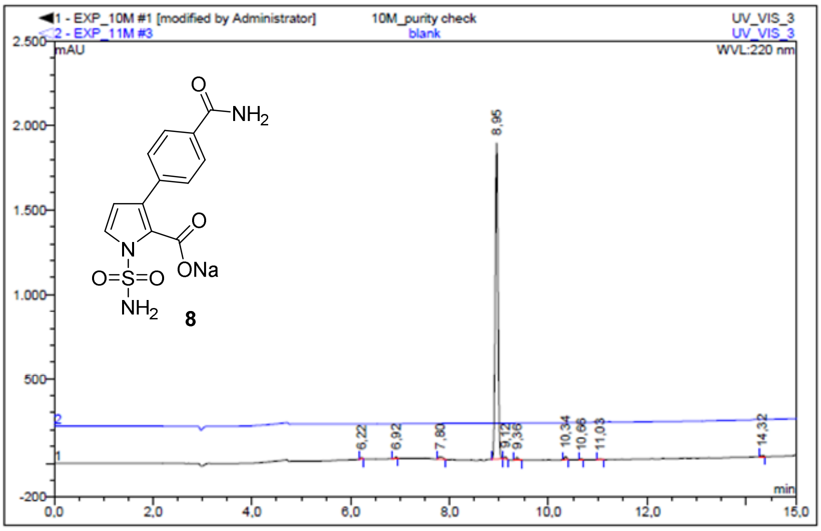

No.	Ret.Time min	Peak Name	Height mAU	Area mAU*min	Rel.Area %	Amount	Resolution(EP)
1	6,22	n.a.	7,494	0,342	0,27	n.a.	8,84
2	6,92	n.a.	8,305	0,390	0,31	n.a.	6,96
3	7,80	n.a.	13,967	1,342	1,06	n.a.	8,28
4	8,95	n.a.	1870,758	121,558	95,63	n.a.	1,89
5	9,12	n.a.	13,497	0,628	0,49	n.a.	2,72
6	9,36	n.a.	16,532	0,988	0,78	n.a.	10,63
7	10,34	n.a.	18,171	0,997	0,78	n.a.	3,56
8	10,66	n.a.	4,741	0,248	0,20	n.a.	3,86
9	11,03	n.a.	2,285	0,162	0,13	n.a.	32,05
10	14,32	n.a.	7,653	0,454	0,36	n.a.	n.a.
Total:			1963,403	127,108	100,00	0,000	

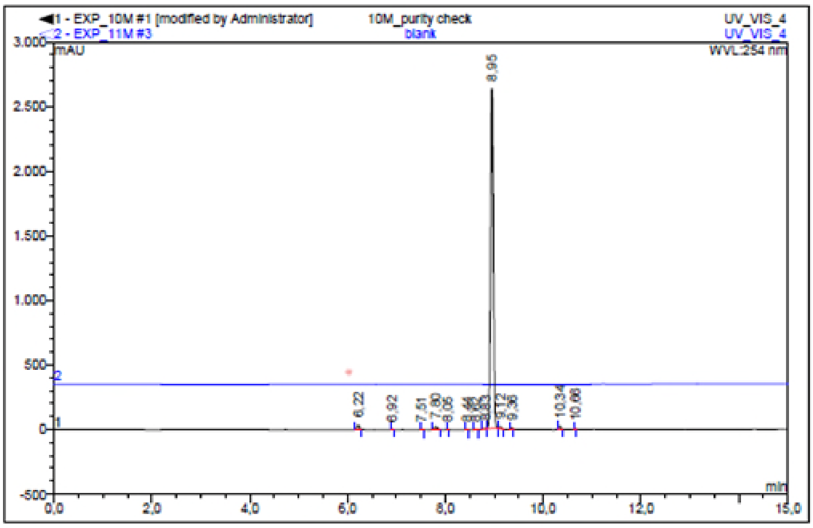

No.	Ret.Time min	Peak Name	Height mAU	Area mAU*min	Rel.Area %	Amount	Resolution(EP)
1	6,22	n.a.	35,015	1,913	1,07	n.a.	9,27
2	6,92	n.a.	2,698	0,094	0,05	n.a.	9,10
3	7,51	n.a.	2,349	0,093	0,05	n.a.	3,49
4	7,80	n.a.	24,152	1,593	0,89	n.a.	3,56
5	8,05	n.a.	0,635	0,014	0,01	n.a.	5,85
6	8,44	n.a.	1,797	0,097	0,05	n.a.	2,04
7	8,62	n.a.	3,545	0,173	0,10	n.a.	1,73
8	8,83	n.a.	1,526	0,130	0,07	n.a.	0,93
9	8,95	n.a.	2632,635	171,854	96,21	n.a.	1,83
10	9,12	n.a.	13,849	0,704	0,39	n.a.	2,83
11	9,36	n.a.	10,388	0,512	0,29	n.a.	11,41
12	10,34	n.a.	24,092	1,296	0,73	n.a.	4,09
13	10,66	n.a.	4,055	0,154	0,09	n.a.	n.a.
Total:			2756,736	178,627	100,00	0,000	

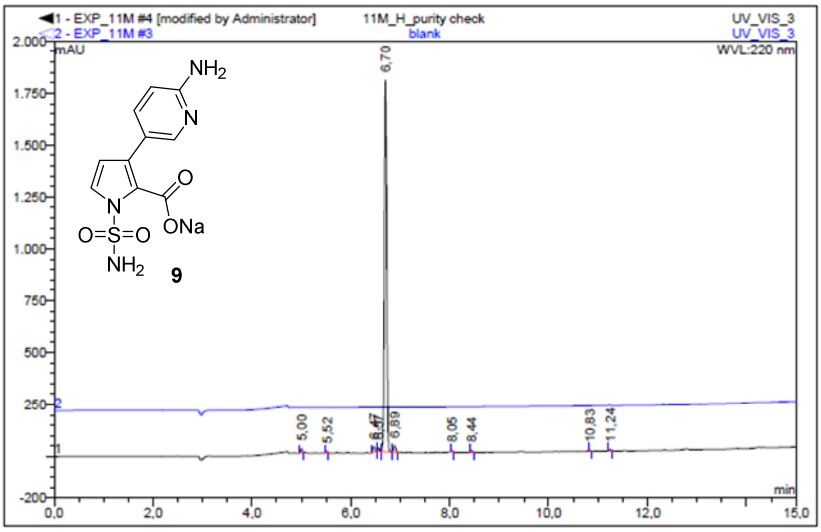

No.	Ret.Time min	Peak Name	Height mAU	Area mAU*min	Rel.Area %	Amount	Resolution(EP)
1	5,00	n.a.	18,879	0,865	0,81	n.a.	7,28
2	5,52	n.a.	6,686	0,254	0,24	n.a.	13,14
3	6,47	n.a.	24,905	1,221	1,15	n.a.	1,35
4	6,57	n.a.	14,985	0,608	0,57	n.a.	1,68
5	6,70	n.a.	1790,478	100,996	94,82	n.a.	2,12
6	6,89	n.a.	30,868	1,627	1,53	n.a.	14,64
7	8,05	n.a.	5,916	0,251	0,24	n.a.	5,20
8	8,44	n.a.	8,047	0,366	0,34	n.a.	35,23
9	10,83	n.a.	2,013	0,065	0,06	n.a.	5,81
10	11,24	n.a.	5,394	0,264	0,25	n.a.	n.a.
Total:			1908,171	106,517	100,00	0,000	

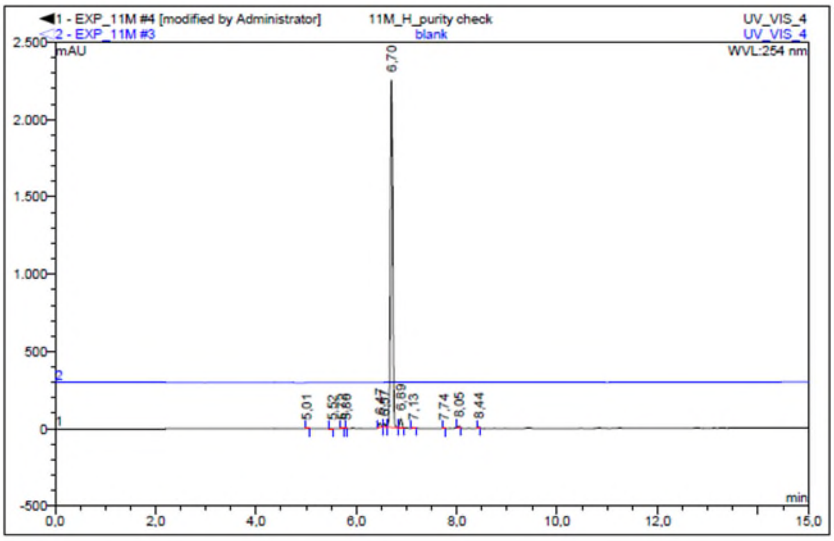

No.	Ret.Time min	Peak Name	Height mAU	Area mAU*min	Rel.Area %	Amount	Resolution(EP)
1	5,01	n.a.	2,165	0,085	0,07	n.a.	8,52
2	5,52	n.a.	0,253	0,009	0,01	n.a.	3,53
3	5,72	n.a.	1,623	0,057	0,04	n.a.	1,75
4	5,80	n.a.	0,787	0,017	0,01	n.a.	11,40
5	6,47	n.a.	29,052	1,390	1,08	n.a.	1,33
6	6,57	n.a.	20,629	0,882	0,68	n.a.	1,68
7	6,70	n.a.	2247,008	122,609	95,01	n.a.	2,16
8	6,89	n.a.	58,729	3,119	2,42	n.a.	2,83
9	7,13	n.a.	2,357	0,119	0,09	n.a.	8,01
10	7,74	n.a.	1,631	0,059	0,05	n.a.	4,42
11	8,05	n.a.	9,987	0,446	0,35	n.a.	5,58
12	8,44	n.a.	6,864	0,254	0,20	n.a.	n.a.
Total:			2381,084	129,047	100,00	0,000	

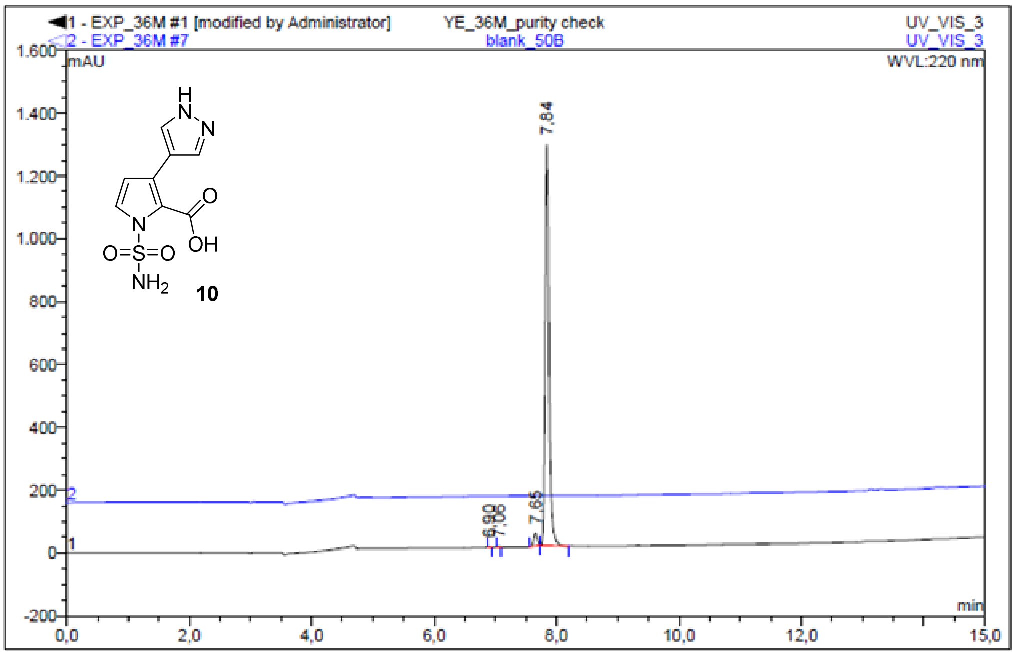

No.	Ret.Time min	Peak Name	Height mAU	Area mAU*min	Rel.Area %	Amount	Resolution(EP)
1	6,90	n.a.	0,978	0,040	0,04	n.a.	2,06
2	7,06	n.a.	1,909	0,091	0,09	n.a.	6,10
3	7,65	n.a.	41,264	2,843	2,83	n.a.	1,65
4	7,84	n.a.	1278,110	97,601	97,04	n.a.	n.a.
Total:			1322,261	100,576	100,00	0,000	

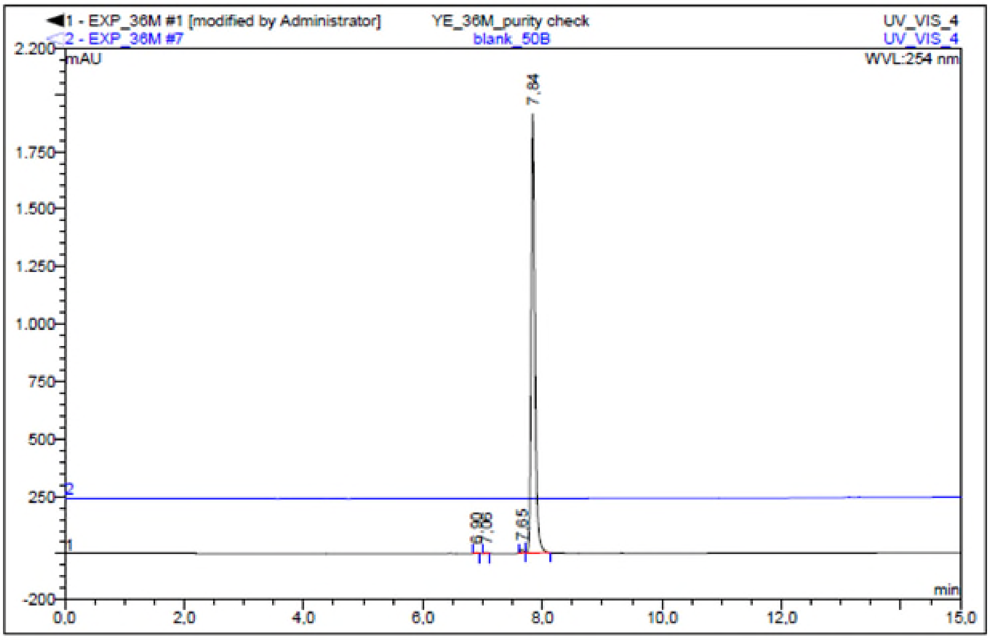

No.	Ret.Time min	Peak Name	Height mAU	Area mAU*min	Rel.Area %	Amount	Resolution(EP)
1	6,90	n.a.	2,716	0,162	0,11	n.a.	1,61
2	7,06	n.a.	3,830	0,222	0,15	n.a.	5,90
3	7,65	n.a.	13,640	0,840	0,57	n.a.	1,71
4	7,84	n.a.	1910,298	145,615	99,17	n.a.	n.a.
Total:			1930,484	146,838	100,00	0,000	

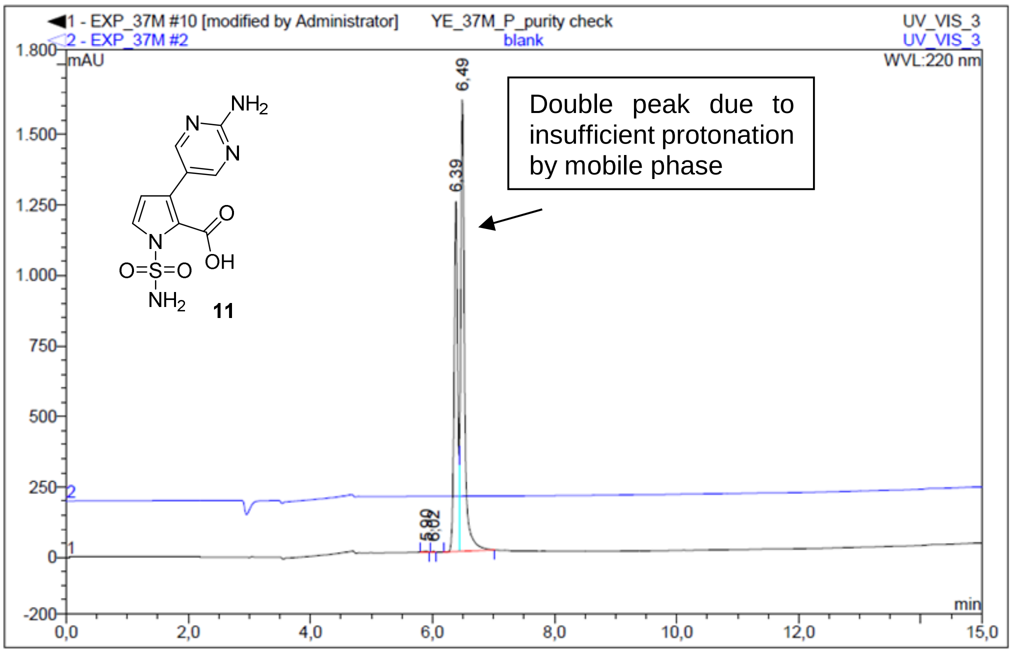

No.	Ret.Time min	Peak Name	Height mAU	Area mAU*min	Rel.Area %	Amount	Resolution(EP)
1	5,90	n.a.	3,347	0,285	0,14	n.a.	1,03
2	6,02	n.a.	2,527	0,108	0,05	n.a.	4,16
3	6,39	n.a.	1241,667	83,203	40,81	n.a.	0,95
4	6,49	n.a.	1601,166	120,299	59,00	n.a.	n.a.
Total:			2848,708	203,895	100,00	0,000	

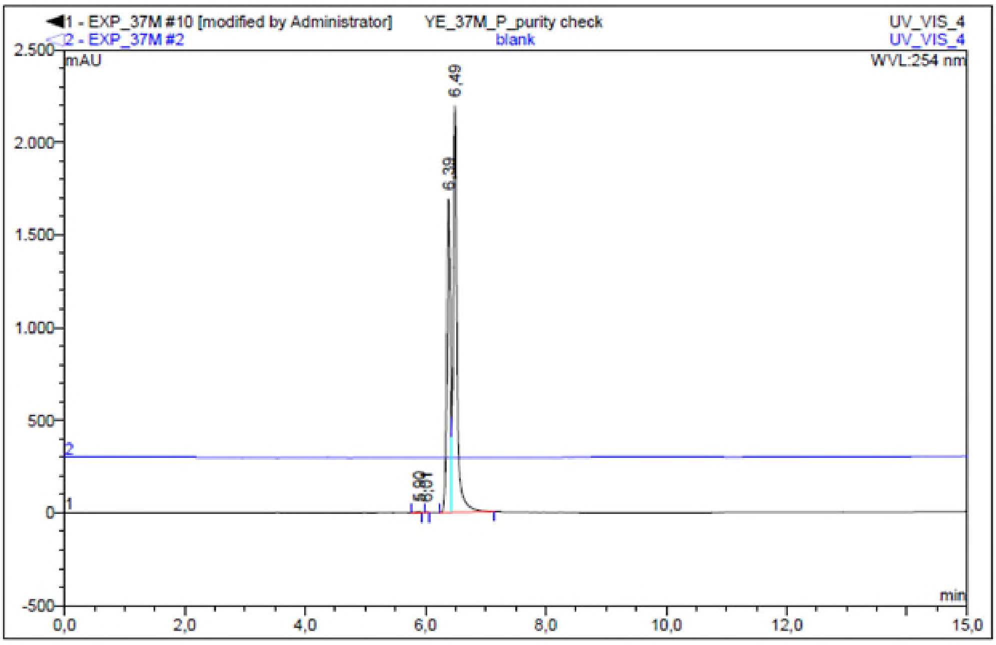

No.	Ret.Time min	Peak Name	Height mAU	Area mAU*min	Rel.Area %	Amount	Resolution(EP)
1	5,90	n.a.	4,062	0,377	0,14	n.a.	0,98
2	6,01	n.a.	2,993	0,124	0,04	n.a.	4,25
3	6,39	n.a.	1691,550	112,846	40,56	n.a.	0,96
4	6,49	n.a.	2194,675	164,863	59,26	n.a.	n.a.
Total:			3893,280	278,209	100,00	0,000	

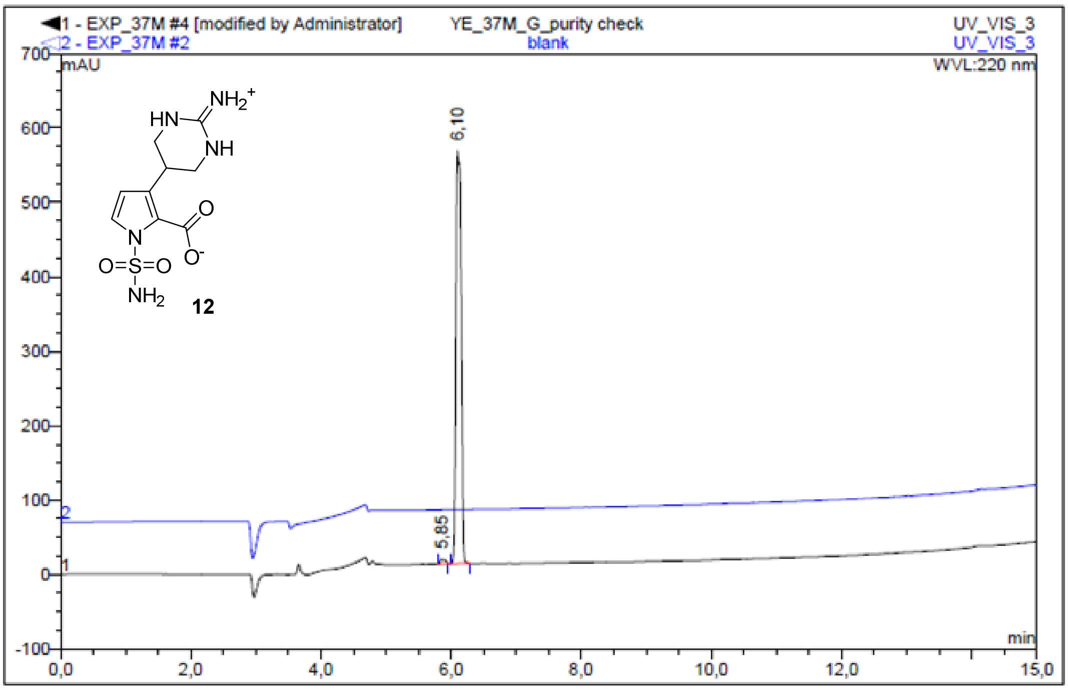

No.	Ret.Time min	Peak Name	Height mAU	Area mAU*min	Rel.Area %	Amount	Resolution(EP)
1	5,85	n.a.	5,811	0,559	1,07	n.a.	1,50
2	6,10	n.a.	554,185	51,561	98,93	n.a.	n.a.
Total:			559,995	52,120	100,00	0,000	

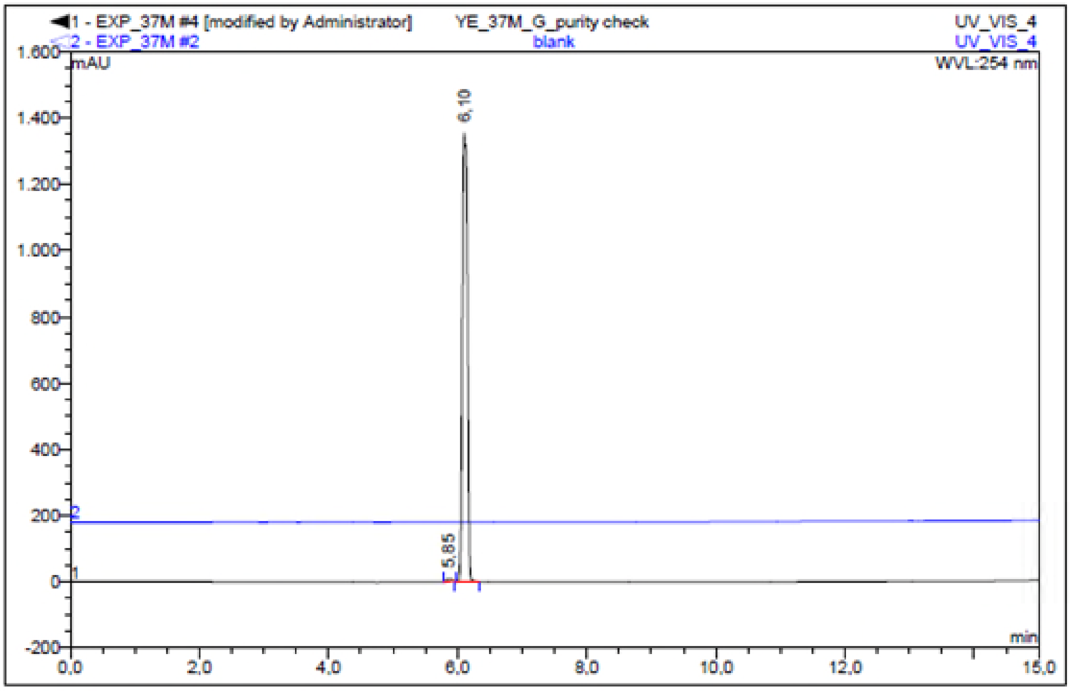

No.	Ret.Time min	Peak Name	Height mAU	Area mAU*min	Rel.Area %	Amount	Resolution(EP)
1	5,85	n.a.	8,481	0,732	0,58	n.a.	1,55
2	6,10	n.a.	1353,873	125,851	99,42	n.a.	n.a.
Total:			1362,354	126,582	100,00	0,000	

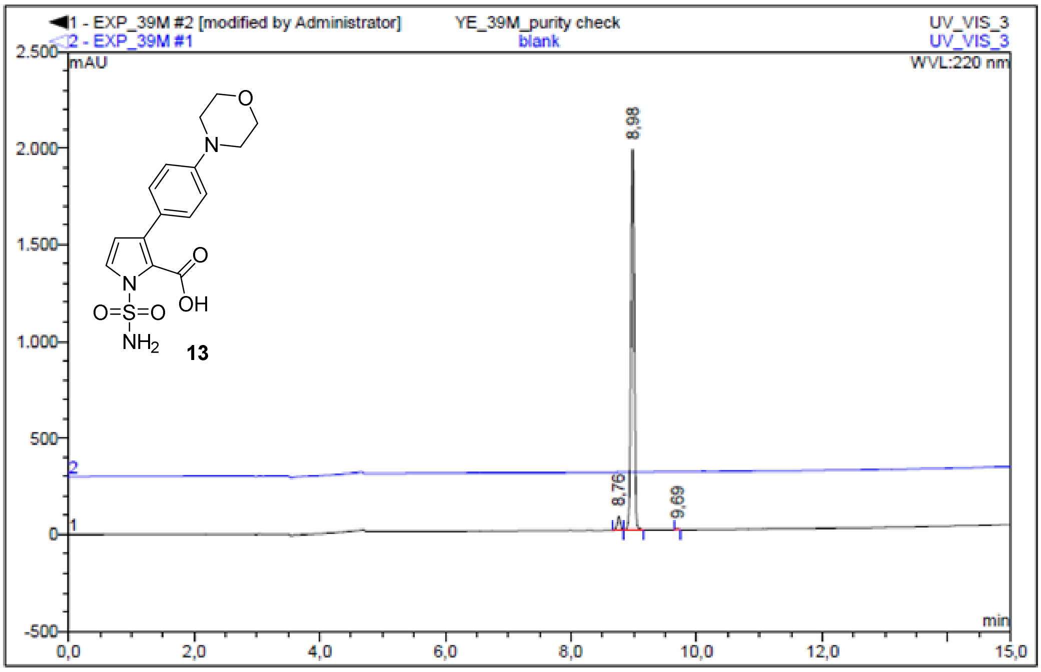

No.	Ret.Time min	Peak Name	Height mAU	Area mAU*min	Rel.Area %	Amount	Resolution(EP)
1	8,76	n.a.	72,876	4,209	3,45	n.a.	2,41
2	8,98	n.a.	1969,376	117,593	96,36	n.a.	8,15
3	9,69	n.a.	4,863	0,234	0,19	n.a.	n.a.
Total:			2047,114	122,036	100,00	0,000	

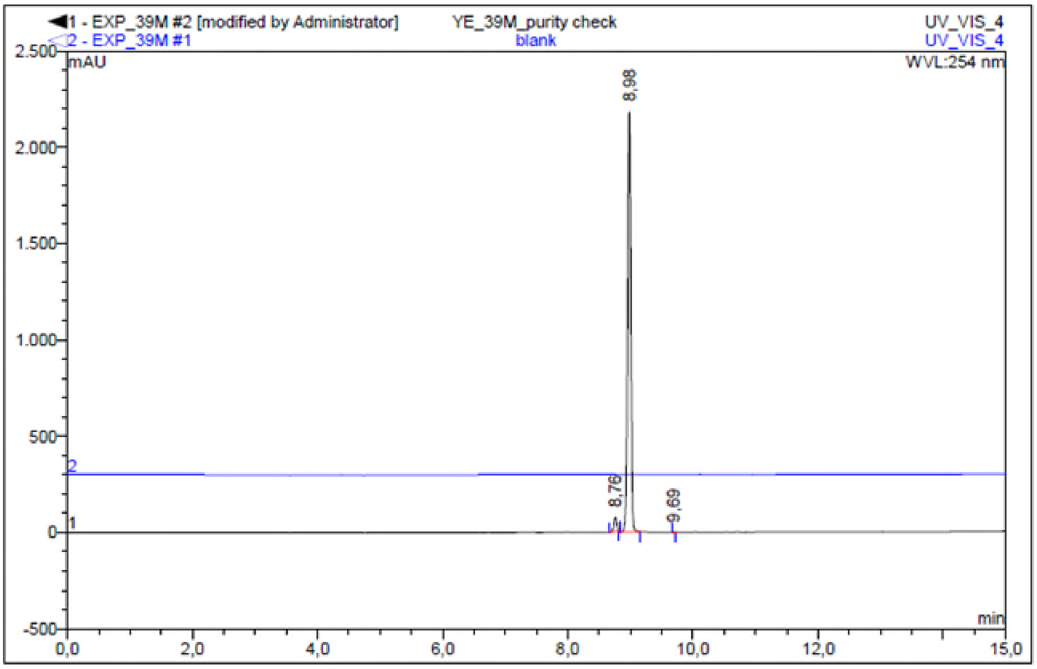

No.	Ret.Time min	Peak Name	Height mAU	Area mAU*min	Rel.Area %	Amount	Resolution(EP)
1	8,76	n.a.	74,796	4,178	3,10	n.a.	2,43
2	8,98	n.a.	2179,941	130,662	96,88	n.a.	9,56
3	9,69	n.a.	0,818	0,026	0,02	n.a.	n.a.
Total:			2255,554	134,865	100,00	0,000	

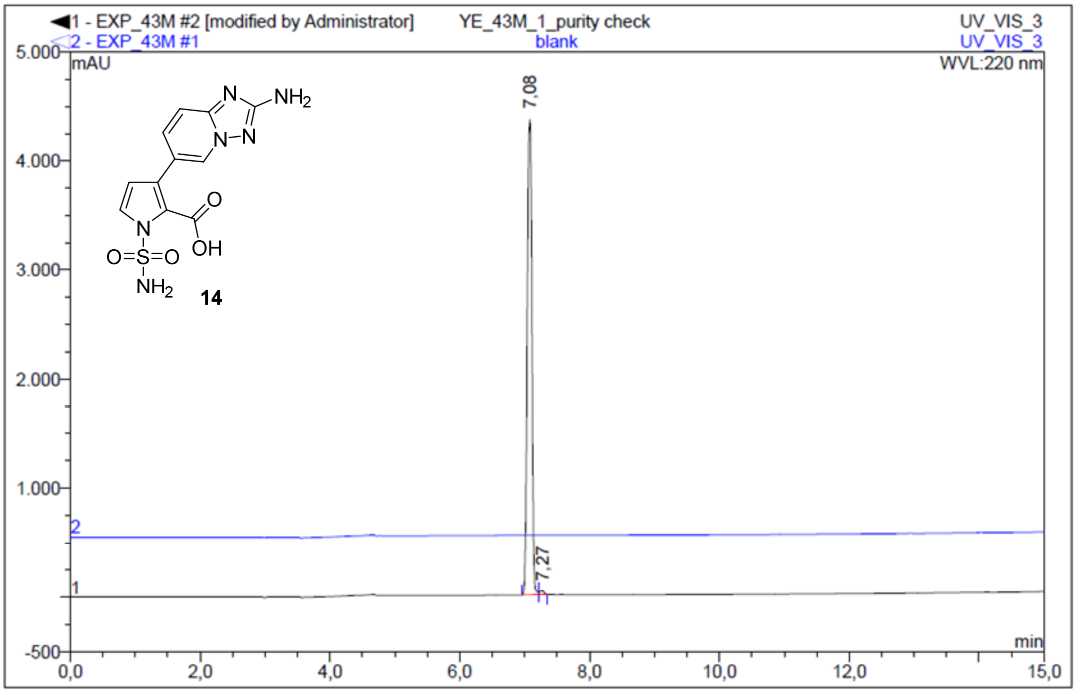

No.	Ret.Time min	Peak Name	Height mAU	Area mAU*min	Rel.Area %	Amount	Resolution(EP)
1	7,08	n.a.	4360,278	366,635	99,33	n.a.	n.a.
2	7,27	n.a.	33,851	2,490	0,67	n.a.	n.a.
Total:			4394,130	369,125	100,00	0,000	

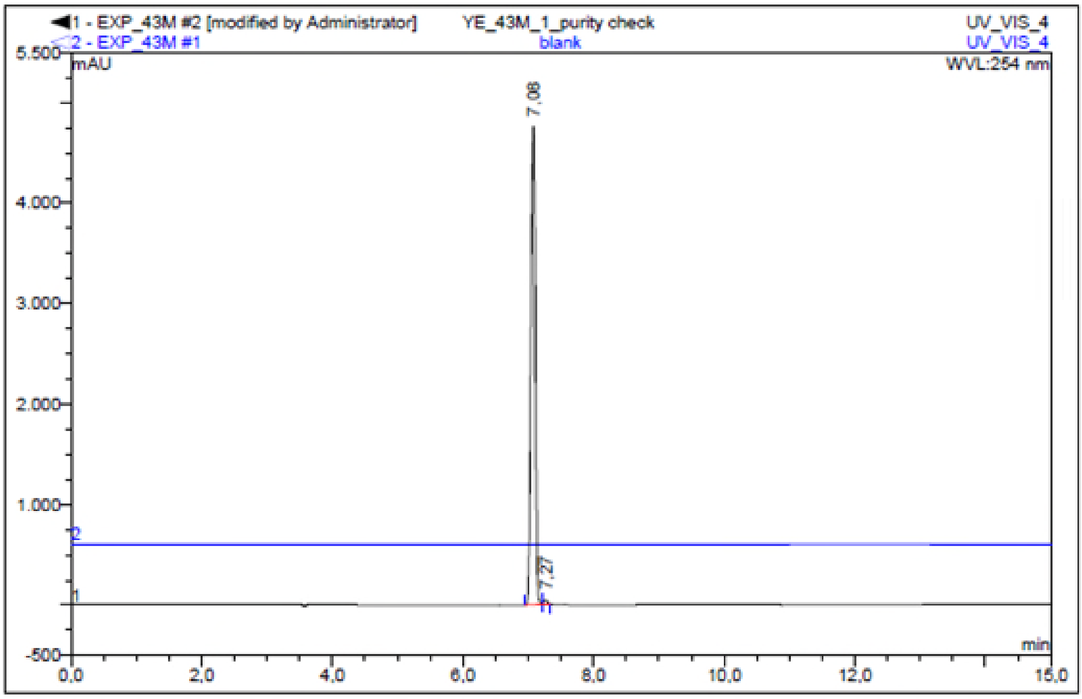

No.	Ret.Time min	Peak Name	Height mAU	Area mAU*min	Rel.Area %	Amount	Resolution(EP)
1	7,08	n.a.	4759,961	365,194	99,14	n.a.	1,53
2	7,27	n.a.	44,875	3,155	0,86	n.a.	n.a.
Total:			4804,837	368,349	100,00	0,000	
